# Human Mast Cells Upregulate Cathepsin B, a Novel Marker of Itch in Psoriasis

**DOI:** 10.3390/cells12172177

**Published:** 2023-08-30

**Authors:** Peter W. West, Chiara Tontini, Haris Atmoko, Orsolya Kiss, Terence Garner, Rajia Bahri, Richard B. Warren, Christopher E. M. Griffiths, Adam Stevens, Silvia Bulfone-Paus

**Affiliations:** 1 Lydia Becker Institute of Immunology and Inflammation, Division of Musculoskeletal and Dermatological Sciences, School of Biological Sciences, Faculty of Biology, Medicine and Health, University of Manchester, Manchester Academic Health Science Centre, Manchester M13 9PT, UK; peter.west@manchester.ac.uk (P.W.W.); chiara.tontini@postgrad.manchester.ac.uk (C.T.); orsolya.kiss@manchester.ac.uk (O.K.); rajia.bahri-2@manchester.ac.uk (R.B.); richard.warren@manchester.ac.uk (R.B.W.); christopher.griffiths@manchester.ac.uk (C.E.M.G.); 2 Division of Developmental Biology and Medicine, Manchester Institute for Collaborative Research on Ageing, School of Biological Sciences, Faculty of Biology, Medicine and Health, University of Manchester, Manchester Academic Health Science Centre, Manchester M23 9LT, UK; terence.garner@manchester.ac.uk (T.G.); adam.stevens@manchester.ac.uk (A.S.); 3 Centre for Dermatology Research, The University of Manchester & Salford Royal NHS Foundation Trust, Manchester Academic Health Science Centre, Manchester M23 9LT, UK; 4 NIHR Manchester Biomedical Research Centre, Manchester University NHS Foundation Trust, Manchester Academic Health Science Centre, Manchester M23 9LT, UK

**Keywords:** mast cells, cathepsin B, psoriasis, itch

## Abstract

Mast cells (MCs) contribute to skin inflammation. In psoriasis, the activation of cutaneous neuroimmune networks commonly leads to itch. To dissect the unique contribution of MCs to the cutaneous neuroinflammatory response in psoriasis, we examined their density, distribution, relation to nerve fibres and disease severity, and molecular signature by comparing RNA-seq analysis of MCs isolated from the skin of psoriasis patients and healthy volunteers. In involved psoriasis skin, MCs and Calcitonin Gene-Related Peptide (CGRP)-positive nerve fibres were spatially associated, and the increase of both MC and nerve fibre density correlated with disease severity. Gene set enrichment analysis of differentially expressed genes in involved psoriasis skin showed significant representation of neuron-related pathways (i.e., regulation of neuron projection along with dendrite and dendritic spine morphogenesis), indicating MC engagement in neuronal development and supporting the evidence of close MC–nerve fibre interaction. Furthermore, the analysis of 208 identified itch-associated genes revealed that *CTSB*, *TLR4*, and *TACR1* were upregulated in MCs in involved skin. In both whole-skin published datasets and isolated MCs, *CTSB* was found to be a reliable indicator of the psoriasis condition. Furthermore, cathepsin B+ cells were increased in psoriasis skin and cathepsin B+ MC density correlated with disease severity. Therefore, our study provides evidence that cathepsin B could serve as a common indicator of the MC-dependent itch signature in psoriasis.

## 1. Introduction

Mast cells (MCs) are uniquely seeded into the skin in early life, occupying stable clonal territories unless or until inflammation occurs [1]. They are commonly found in close proximity to nerve fibres [2,3], especially those expressing substance P (SP) and calcitonin gene-related peptide (CGRP) [4,5,6,7]. Mast cells make direct membrane–membrane contact with nerve fibres [8,9]. Cytokines and granule-associated mediators released by MCs, such as TNF-α, interleukin (IL)-1b, IL-6, and IL-17A, have been shown to act on the nervous system and promote pain. Furthermore, MCs synthesize and release nerve growth factor (NGF), known to induce inflammatory hyperalgesia [10] and neurite outgrowth and synaptogenesis in the brain as well as to enhance CGRP expression in dorsal root ganglia sensory neurons [11].

Mast cells are known to regulate the extent of skin inflammation [12,13], and MC activation via the Mas-Related G Protein-Coupled Receptor-X2 (MRGPRX2) may be required for inflammatory neuronal signalling [14,15] and PAMP1-20 induced itch [16]. Moreover, in certain inflammatory situations such as psoriasis, activation of MCs by IL-33 serves as a primary source of TNF-α [17,18] while regulating type two inflammation [19,20], influencing neurite outgrowth [21].

Studies have established the potential for MC activity in psoriasis. Degranulated MCs are consistently found to be increased in involved psoriasis skin [22,23] and during the occurrence of new lesions [24]. Serum total IgE and tryptase are raised in psoriasis [25,26], the latter implicated in the generation of the autoantigen Pso p27, found to be increased in lesional skin and possibly involved in disease chronicity [27]. In addition, MCs can capture, store, and release exogenous IL-17A [28], and can release extracellular traps and degranulate upon stimulation of IL-23 and IL-1β [29], which are major cytokines involved in psoriasis pathogenesis [30]. However, despite the indirect evidence, the individual contribution of MCs in psoriasis remains to be fully elucidated. In psoriatic lesions, a number of inflammatory markers such as kallikreins (KLKs) [31], alarmins such as IL-33 [17], and TNF-α are predictably augmented [32,33]. Furthermore, an increase in MC-activating neuropeptides such as SP and vasoactive intestinal peptide (VIP) [34], along with SP-, Transient Receptor Potential Vallinoid 1 (TRPV1)-, and Neurokinin-1 Receptor (NK1R)-positive nerve fibres [35,36,37]. The neuropeptides SP, CGRP, and VIP induce skin MCs to degranulate and promote the release of pro-inflammatory mediators, including TNF-α and IL-3 [38,39]. In human skin, MCs express SP [40] and VIP [41], suggestive of autocrine or paracrine activity. Therefore, during disease the MC–nerve axis may drive feed-forward neurogenic inflammation and regulate allo- and hypersensitivity through as yet undefined neuroplastic mechanisms [42].

Pruritus is a frequent symptom in psoriasis, and affects 70% to 90% of patients [43,44,45,46]. Although its pathophysiology is not fully understood, the contribution of MCs to acute itch through the release of histamine is well-defined [47,48,49,50]. Therefore, histamine-mediated MC–nerve communication is thought of as the archetypal paradigm of the neuroimmune axis [50,51]. While antagonists of the histamine H_1_, H_2_, and H_3_ receptors have not been found to inhibit pruritus in chronic inflammatory skin diseases, including psoriasis and atopic dermatitis [52], it is now appreciated that a plethora of non-histaminergic factors, both directly and indirectly dependent on MC degranulation, act to selectively induce action potential discharge from pruritic neurons [48,50]. Highly selective activation of MCs induces itch in mice via LTC_4_, serotonin, and S1p [53]. Additionally, MCs are known to release other pro-pruritic substances such as tryptase [54], lipid mediators (prostaglandins, phospholipase A2, LTB_4_) [55,56,57], and cytokines (IL-4, IL-13, IL-25 and IL-31) [58,59,60,61], though the specific contribution of MCs in the overall pruritic context is not well defined.

Through this study, we sought to better define the involvement of MCs in psoriasis and the potential role of neuroimmune interactions in the pathogenesis of psoriatic itch.

## 2. Materials and Methods

### 2.1. Human Skin Samples

Normal and involved psoriasis skin punch biopsies were obtained from the Dermatopharmacology Department in Salford Royal Hospital through clinical studies according to protocols approved by the local NHS research ethics committee (13/NW/0867 and 10/H1005/77). Normal human skin punch biopsies were obtained through the Manchester skin health biobank (14/NW/0185). All subjects provided written informed consent in accord with the declaration of Helsinki. Study subject characteristics are shown in Supplementary Tables S2 and S3. Three of the healthy volunteer samples had previously been analysed as controls in another study [62].

### 2.2. Acquisition of Human Skin Biopsies

Punch biopsy specimens 6 mm in diameter were obtained under local anaesthesia from sun-protected normal and involved psoriasis buttock skin, and the psoriasis area severity index (PASI) scores for psoriasis patients were calculated by a trained specialist nurse. Patients were asked to score how intensely they felt their skin itching before the biopsy was taken on a scale from 1 to 10, with 1 indicating no itch and 10 indicating the worst itch imaginable. Additionally, a worst itch numerical scale was used whereby each psoriasis patient was asked for the intensity of the most intense itch they had felt in the last 24 h before the biopsy was taken on a scale from 1 to 10, with 1 indicating no itch and 10 indicating the worst itch imaginable.

### 2.3. Immunostaining

Paraffin-embedded skin sections (5 μm) were de-paraffinized in xylene and rehydrated in descending grades of alcohol. Rehydrated slides were boiled in sodium citrate buffer (10 mM, pH6) or Tris-EDTA buffer (10 mM Tris Base, 1 mM EDTA) for 20 min for antigen retrieval and blocked in 10% normal goat serum PBS-Tween 20 (0.1% *v*/*v*) buffer for 30 min at room temperature. Mast cell and nerve fibre or MC and cathepsin B cell density was determined using dual immunofluorescent staining. This is an established technique used to identify MCs and neuropeptides in psoriasis and other tissues [63,64,65]. Blocked sections were incubated with anti-tryptase clone AA1 (Abcam, ab2378, 1:1000, Cambridge, UK) for an hour at room temperature, then incubated with anti-PGP 9.5 (Abcam, ab108986, 1:100) or anti-cathepsin B recognising pro- and heavy chain-cathepsin B (#31718 Cell Signaling Technology, Leiden, The Netherlands) overnight at 4 °C. The next day, slides were incubated with secondary antibodies (anti-mouse Alexa Fluor 488, Abcam ab150117; anti-rabbit Alexa Fluor 555, Abcam, ab150086, 1:200) or (anti-rabbit Alexa Fluor 488, ThermoFisher Scientific, Paisley, UK; anti-mouse Alexa Fluor 647, Abcam ab150119) for 30 min. Substance P and VIP density and their spatial association with MCs were quantified by triple immunostaining. Following the serum block, slides were incubated with anti-tryptase (Abcam, ab2378, 1:1000) for an hour at room temperature, then incubated with anti-PGP 9.5 (Abcam, ab10410, 1:400) and anti-SP (Millipore, AB1566, 1:100, Livingston, UK) or with anti-PGP 9.5 (Abcam, ab10410, 1:400) and anti-VIP (Abcam, ab8556, 1:25) overnight at 4 °C. The following day, sections were labelled with secondary antibodies (anti-Guinea pig Alexa Fluor 488, ThermoFisher Scientific, A11073; anti-rabbit Alexa Fluor 555, Abcam, ab150086; anti-mouse Alexa Fluor 647, Abcam ab150119, 1:200) for 30 min at room temperature. Sections were triple stained for tryptase (Abcam, ab134932, 1:50), CGRP (Abcam, ab81887, 1:50) and PGP 9.5 (Abcam, ab10410, 1:400). Primary antibodies were incubated overnight at 4 °C, then incubated with the secondary antibodies (anti-rabbit Alexa Fluor 647, Abcam, ab150083; anti-mouse Alexa Fluor 555, Abcam ab150118, anti-Guinea pig Alexa Fluor 488, Invitrogen, A11073, 1:200) for 30 min at room temperature on the following day. After labelling steps, sections were mounted using Fluoroshield mountant containing 4′,6-diamidino-2-phenylindole (DAPI) (Abcam, UK) prior to image analysis.

Images were collected on a Zeiss Axioimager.D2 upright microscope using a 20×/0.5 EC Plan-neofluar objective and captured using a Coolsnap HQ2 camera (Photometrics) through Micromanager software v1.4.23. Images were captured using an Olympus BX53 upright microscope with a 20×/0.75 UPlanS Apo objective and an Olympus DP73 camera with Olympus cellSens v1.18 software. Both microscopes utilised specific bandpass filter sets for DAPI, FITC, Cy3, and Cy5 which were used to prevent bleedthrough between channels. Images were then processed and analysed using Image J (v1.53g) software (https://imagej.nih.gov/ij/download.html accessed on 7 January 2021). Images were false coloured with cyan, magenta, and yellow to enable greater colour accessibility. The distance between each mast cell and the closest staining for PGP or SP/CGRP and VIP was measured manually using calibrated images in ImageJ. The density of MCs was calculated as the number of MCs per measured area of dermis in mm^2^ taken from *n* = 10 nonoverlapping calibrated images for each subject, in Image J. Similarly, the density of neuropeptides was calculated from the stained area of nerves as a proportion of the total dermal area in the same number of non-overlapping images. Mean densities per subject were correlated with Psoriasis Area and Severity Index (PASI) using Pearson’s correlation statistic. Analysis of cathepsin B positive cells was restricted to the dermis only. Cellular cathepsin B counts were limited to colocalization with MC tryptase in the case of MCs or presence of nuclei in the case of other cellular stainings. Single colour channels of representative composite photomicrographs are shown in the supplementary data (Supplementary Figure S5).

### 2.4. Mast Cell Isolation from Skin

Mast cells were isolated from 6 mm biopsies obtained from 6 mm skin punch biopsies acquired from the buttocks of volunteers with and without psoriasis under local anaesthesia in the Salford Royal Foundation Trust Hospital Dermatopharmacology Unit. Biopsies were stored in ice-cold PBS. Skin biopsies were cut into small pieces in digestion solution containing RPMI 1640 medium (Sigma-Aldrich, Gillingham, UK), 0.5% BSA (ThermoFisher Scientific), 100 U.mL^−1^ penicillin and 100 µg.mL^−1^ streptomycin, 0.5 Wunch units.mL^−1^ of Liberase TM (Roche, Basel, Switzerland), and 100 ng.mL^−1^ of SCF (Genscript, Piscataway, NJ, USA), then incubated overnight at 37 °C, 5% CO_2_.

Digested skin was passed through a 40 μm mesh cell strainer and cell concentration was adjusted to 50 × 10^6^ cells.mL^−1^. Cell suspension was stained for 15 min in PBS 0.5% BSA, 2 mM EDTA with CD45 (2D1), Human Lineage cocktail (CD3 (UCHT1), CD14 (HCD14), CD19 (HIB19), CD20 (2H7) and CD56 (HCD56)), CD117 (104D2), FcɛRIα (AER-37) antibodies (all from BioLegend, San Diego, CA, USA), and a viability dye (Fixable Blue Dead Cell Stain Kit, ThermoFisher Scientific). CD45+, Lin-, CD117+, FcɛRIα+, and MCs were sorted using a BD Influx (BD Biosciences) (Supplementary Figure S3), collected in 1000 cell aliquots and stored at −80 °C until required.

### 2.5. Sample Preparation for RNAseq

A SMART-Seq^®^ Ultra^®^ Low Input RNA Kit was used to synthesize the full cDNA from 1000 MCs according to the manufacturer’s instructions (Takara Bio, London, UK). The analysis was performed on the next-generation sequencing Illumina HiSeq platform (Illumina, Cambridge, UK).

### 2.6. RNAseq Data Preprocessing

Obtained raw files were trimmed with Trimomatic (v0.36) using default settings and aligned to the human genome assembly hg19 using STAR (v2.5.3) and Gencode annotation v16. Aligned reads were sorted and filtered using Samtools (v1.3). Ordered BAM files were uploaded to Qlucore Omics Explorer (version 3.3, Lund, Sweden) for gene expression analysis.

### 2.7. Gene Expression Analysis

Microarray datasets of published studies comparing the expression of genes in involved psoriatic skin with normal skin were acquired from the National Centre for Biotechnology Information GEO Datasets (https://www.ncbi.nlm.nih.gov/gds/).

The microarray dataset GDS4602 reference series GSE13355 (https://www.ncbi.nlm.nih.gov/sites/GDSbrowser?acc=GDS4602, last accessed 4 March 2021) [66] contained data for 64 normal and 58 involved psoriatic skin samples. The microarray dataset GSE80047 (https://www.ncbi.nlm.nih.gov/geo/query/acc.cgi?acc=GSE80047, last accessed 4 March 2021) contained data for 24 normal and 10 involved psoriatic skin samples [67]. The microarray dataset GSE78097 (https://www.ncbi.nlm.nih.gov/geo/query/acc.cgi?acc=GSE78097, last accessed 4 March 2021) contained data for 6 normal samples and 13 involved psoriasis skin samples [68].

Two-group comparison analysis comparing involved psoriatic versus normal skin samples was conducted using Qlucore software (v3.6) (Qlucore AB, Lund, Sweden). Log-normalized counts obtained from normal and involved psoriatic skin samples from isolated MCs, as well as the whole skin datasets GDS4602, GSE80047, and GSE78097 were extracted from Qlucore and used for principal component analysis, which was conducted in R using the mixOmics package [69]. Three-dimensional PCA plots were generated from the log-transformed normalized matrix from Qlucore using the pca and plotIndiv functions of the mixOmics R package. Heatmaps and volcano plots were generated using the R packages pheatmap [70] and EnhancedVolcano [71], respectively.

Genes with a *p*-value threshold of 0.05 after false discovery rate adjustment (q) were deemed significant, and log2 fold change values were used to determine upregulation (log2 fold change < 0) or downregulation (log2 fold change > 0) relative to normal skin controls. Venn diagrams were created using the R package ggvenn [72].

### 2.8. Gene Set Enrichment Analysis

Gene set enrichment analysis (GSEA) was carried out on a list of ranked genes based on *p*-values and the sign of the log2 fold changes for isolated MCs and the whole skin datasets. GSEA was computed using the R packages clusterProfiler [73] and ReactomePA [74]. Three ontology databases were used, namely Gene Ontology (GO), the Kyoto Encyclopedia of Genes and Genomes (KEGG), and REACTOME. Significance was set at *p*-value of < 0.05 adjusted for false discovery rate. Scatterplots were created using the ggplot2 R package. Plots of enrichment scores were created using the R package enrichplot.

### 2.9. Ingenuity Pathway Analysis

Datasets of pre-filtered significant genes (q < 0.05) of isolated MCs and the whole skin datasets GDS4602, GSE80047, and GSE78097 were uploaded to Ingenuity Pathway Analysis (IPA) software v01.20.04 (Qiagen). Core analysis was run individually on each dataset, and analysis of canonical pathways, upstream regulators, and causal networks was conducted. Comparison analysis was then conducted between different datasets to assess the differences in predicted activation z-scores and the significance between isolated MCs and whole skin datasets. Significance was set at *p* < 0.05 for the Fisher exact test in each analysis.

### 2.10. xCell Analysis

Cell type enrichment analysis of publicly available datasets and isolated MCs from normal skin and psoriasis lesions was performed using the xCell webtool [75] for microarray data and RNA-seq data. xCell scores and significance levels were obtained for each donor/condition. The proportion of samples in which significant (*p* < 0.05) MC and neuron enrichment was detected was displayed per study. Isolated MCs were added as internal control to assess the efficacy of the algorithm in picking up MC-specific signatures.

### 2.11. Random Forest Analysis

Genes were assessed for importance in predicting psoriatic from control tissue using Boruta (v.7.0.0) [76]. Those that were initially determined to be important were assessed again using Boruta to refine the number of genes and avoid overfitting the random forest model. Random forest analysis was conducted on pre-selected itch-associated genes (*n* = 208) and run using 500 trees with the Rattle v.5.4.0 R package [77]. The estimation of the area under the curve (AUC) for sensitivity and specificity analysis of the random forest model and out of bag (OOB) AUC for estimation of the prediction and error rate of the model applied to the validation set were calculated using Rattle.

### 2.12. Peripheral Blood-Derived Human Mast Cell Culture and Cathepsin B ELISA

Human peripheral blood NC24 leukocyte cones obtained from anonymous healthy donors were obtained from NHS blood and transplant (Manchester, UK) and used in accordance with a protocol approved by the University of Manchester Research Ethics Committee (UREC ref 2018-2696-5711). Progenitor cells were isolated and cultured as previously described [78]. Mature MCs were activated by SP (Genscript, Oxford, UK) at the indicated concentrations for 1 h. Cell-free supernatants were collected, and human total cathepsin B content was analysed by ELISA (R&D Systems, Abingdon, UK) according to the manufacturer’s instructions.

### 2.13. Statistical Analysis

Graphing and analyses were performed using Prism v9.1.2 (GraphPad, San Diego, CA, USA). Data were subject to normality tests, and inter-group comparisons were made using appropriate parametric (unpaired *t*-test) or non-parametric (Mann–Whitney test) methods. Correlation statistics were generated from a simple linear regression model. Concentration response curves were generated from the four-parameter logistic regression least squares fit to the raw data.

## 3. Results

### 3.1. In Psoriasis Skin, Mast Cells Are Spatially Associated with Neuropeptide Positive Nerve Fibres and Their Density Correlates with Disease Severity

While MCs are consistently increased [23,79], nerve fibres have been found to be both heightened [36] as well as diminished [80] in psoriasis patients’ skin. Colocalisation of MCs with nerve fibres suggests increased cross-talk, indicating exposure of the latter to neurotrophic and itch mediators. However, both small-diameter DRG afferents and cholinergic sympathetic nerve fibres can express neuropeptides [81,82] that activate human MCs [83,84,85]. In psoriasis, the role of neuropeptides is unclear, with conflicting findings having been published [34,79,86,87,88]. Therefore, the association between MCs and nerve fibre markers in skin from psoriasis lesions and healthy volunteers was investigated.

**Figure 1:**
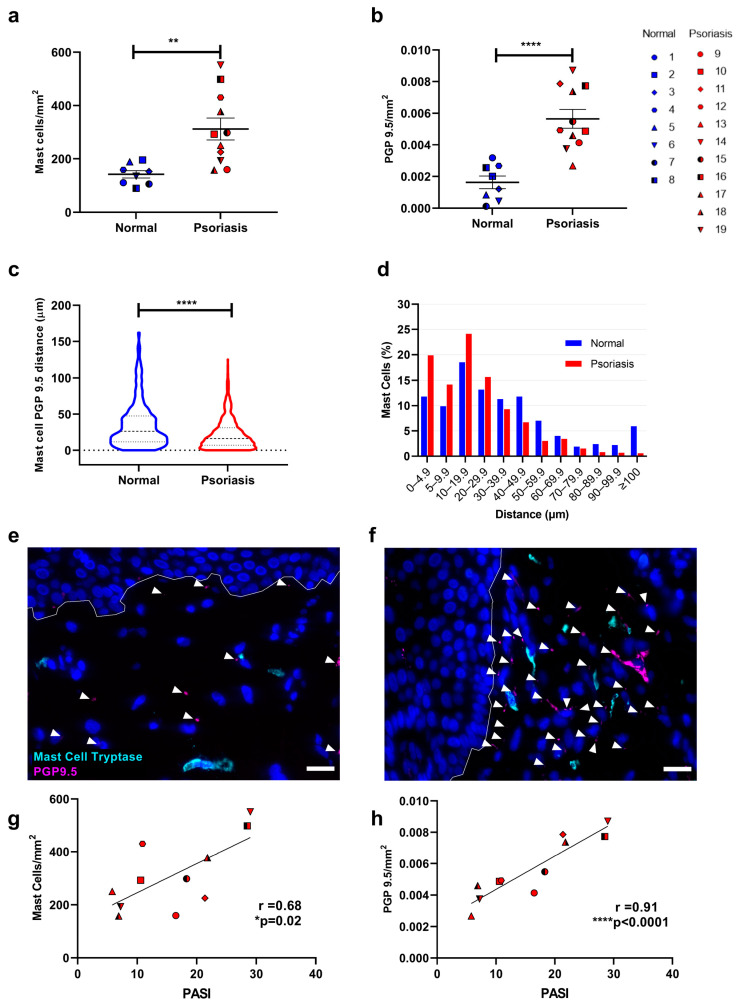
Mast cell and nerve fiber density, spatial association, and correlation with psoriasis severity. Immunofluorescence microscopy was used to identify and measure the density of (**a**) dermal MC tryptase and (**b**) PGP 9.5+ nerve fibres in normal (*n* = 8) and involved psoriasis skin (*n* = 11). Each subject is represented by a different symbol. (**c**,**d**) The distance in µm between MCs and PGP9.5+ nerves and the normalised frequency distribution of MCs within a given distance of a PGP9.5+ nerve fibre. (**e**,**f**) Representative photomicrographs of normal (**e**) and involved (**f**) skin show MC tryptase (cyan) and PGP9.5 (magenta). Discrete areas of PGP9.5 immunofluorescence in the dermis are identified (arrowheads). Scale bar = 20 µm. The tissue density of tryptase-positive MCs (**g**) and PGP9.5-positive nerve fibres (**h**) was correlated with the psoriasis severity index (PASI). * *p* < 0.05, ** *p* < 0.01, **** *p* < 0.0001, (unpaired *t*-test (**a**,**b**) Mann–Whitney U test (**c**), Pearson correlation (**g**,**h**)).

Confirming previous studies [89], the frequency of MCs in sections from psoriasis lesions (mean frequency of 312.3 ± 40.8/mm^2^ (mean ± SEM ** *p* < 0.0032) was significantly increased compared to normal skin (142.1 ± 13.7/mm^2^) (Figure 1a and Supplementary Table S1a). The PGP9.5 immunoreactive nerve fibre density was significantly higher in psoriasis lesions compared to normal skin (mean frequency of 0.0057 ± 0.0006/mm^2^ (mean ± SEM **** *p* < 0.0001) versus 0.0016 ± 0.0004/mm^2^) (Figure 1b). MCs were significantly closer to PGP9.5+ nerve fibres in the dermis of involved psoriasis lesions (16.23 (6.99–30.99) µm) compared to normal skin (26.37 (11.66–47.36) µm; Median (IQR) **** *p* < 0.0001) (Figure 1c). Furthermore, in involved skin, the normalized percentage of MCs within 5µm of a nerve fibre was nearly double (19.9%) that of normal skin (11.8%) (Figure 1d and Supplementary Table S1b–d). Representative photomicrographs of normal and involved skin are shown (Figure 1e,f). Additionally, both the MCs and nerve fibre density significantly correlate with psoriasis severity as measured by PASI (r = 0.69, *p* = 0.02; r = 0.91, *p* < 0.0001).

**Figure 2:**
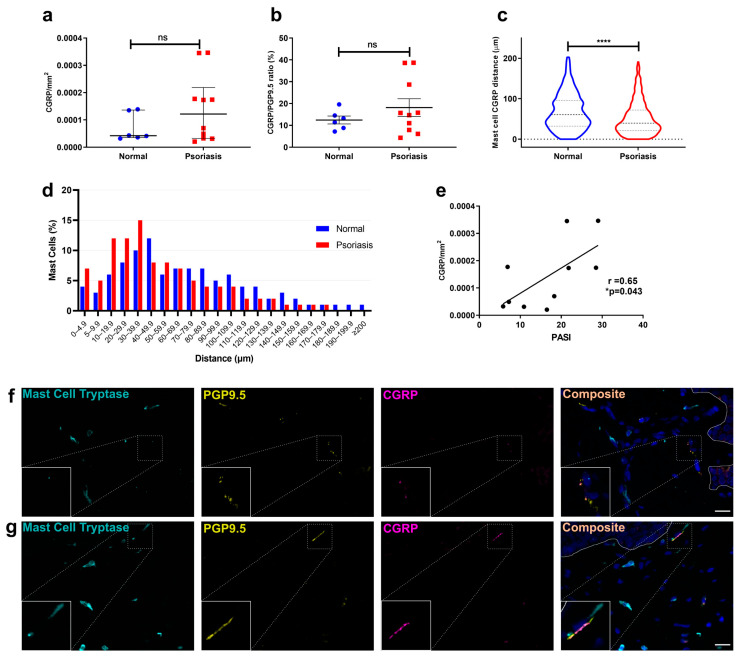
Calcitonin gene-related peptide density, spatial association with MCs, and correlation with severity of psoriasis. Immunofluorescence microscopy was used to identify and measure the density of MCs (tryptase), nerve fibres (PGP 9.5), and CGRP in normal (*n* = 6) and involved psoriasis skin (*n* = 10). (**a**) CGRP nerve fibre density and (**b**) proportion of CGRP+ nerve fibres in skin sections. (**c**) Distance between MC and CGRP+ nerve fibres and (**d**) normalised frequency distribution of MCs within a given distance of a CGRP+ nerve fibres in skin. (**e**) Correlation between CGRP density and severity index (PASI). (**f**,**g**) Representative photomicrographs of normal (**f**) and involved (**g**) skin show MC tryptase (cyan), PGP9.5 (yellow), and CGRP (magenta). The inset panel shows a magnified area with colocalized fluorescence. Scale bar = 20µm. Data are median ± IQR of *n* = 6 and *n* = 10 donors. * *p* < 0.05, **** *p* < 0.0001, ns: not significant (Mann–Whitney U test (**a,c**), unpaired *t*-test (**b**), Pearson correlation (**e**)).

While CGRP density and CGRP/PGP 9.5 ratio (Figure 2a,b) were not significantly altered in psoriatic lesions compared to normal skin (*p* = 0.37 & *p* = 0.31 respectively), MCs showed significantly increased proximity to CGRP+ nerve fibres 60.35 (32.16–96.03) μm in normal vs. 39.56 (21.2–71.95) μm (Median (IQR) **** *p* < 0.0001) in involved skin, with the percentage of MCs within 40 µm of a CGRP+ nerve fibre increasing (Figure 1c,d). CGRP density was found to correlate significantly with psoriasis disease severity, (r = 0.65, *p* = 0.043) (Figure 2e). Representative photomicrographs are shown in Figure 2f,g.

This was not the case for the neuropeptides SP or VIP, for which no significant increases in density, MC association, or significant correlation with PASI was observed (Supplementary Figures S1 and S2).

Thus, our findings indicate that in psoriasis, MCs and nerve fibres both increase in number and correlate with disease, while their proximity is increased. MCs were predominantly and uniquely associated with CGRP+ nerve fibres, suggesting a role in disease. Therefore, we proceeded to investigate the potential impact of this association on MCs and itch-related gene expression.

### 3.2. Mast Cells from Psoriasis Skin Exhibit Enriched Expression of Neuronal Development-Associated Genes

To explore the relative contribution of MCs to psoriasis pathogenesis, we analysed the transcriptional signature of MCs isolated from skin by cell sorting (Supplementary Figure S3, Supplementary Table S2).

**Figure 3:**
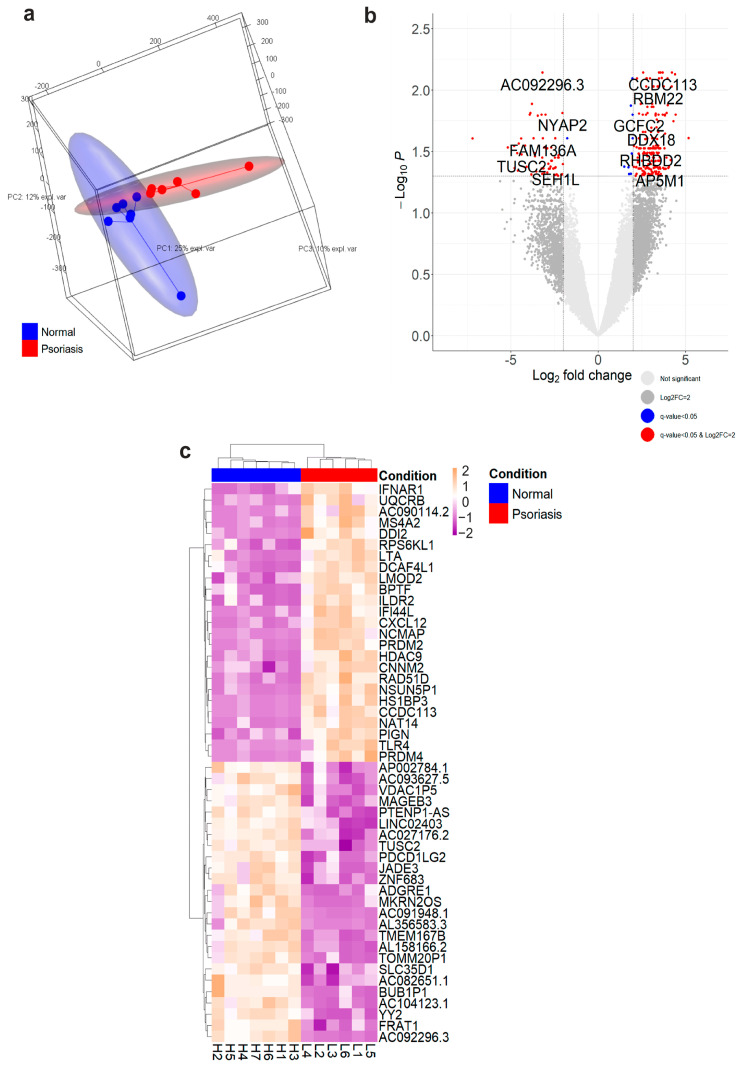
RNA-seq analysis of mast cells isolated from normal and psoriasis skin. Mast cells (MCs) were isolated from normal (*n* = 7) and psoriasis-involved skin (*n* = 6, 2 donors pooled): (**a**) 3D PCA plot of log-transformed normalized matrix showing confidence ellipses (PCA1: 25.5%, PCA2: 12.42%, PCA3: 9.5% of explained variance) and (**b**) volcano plot of DEGs. Significantly different genes based on FDR-corrected *p* values and log2 fold change ± 2 are highlighted in red. (**c**) Z-score heatmap of the top 50 DEGs (q < 0.05) ordered by log2 fold change.

Differential gene expression analysis of MCs isolated from involved psoriasis and normal skin revealed a total of 249 differentially expressed genes (Supplementary Table S4). Principal Component Analysis showed separation between normal and involved skin MCs, with 25% and 12% of variation between subjects explained by components 1 and 2, respectively (Figure 3a). The volcano plot and heatmap of the top 50 significantly differentially expressed genes are displayed in Figure 3b,c.

**Figure 4:**
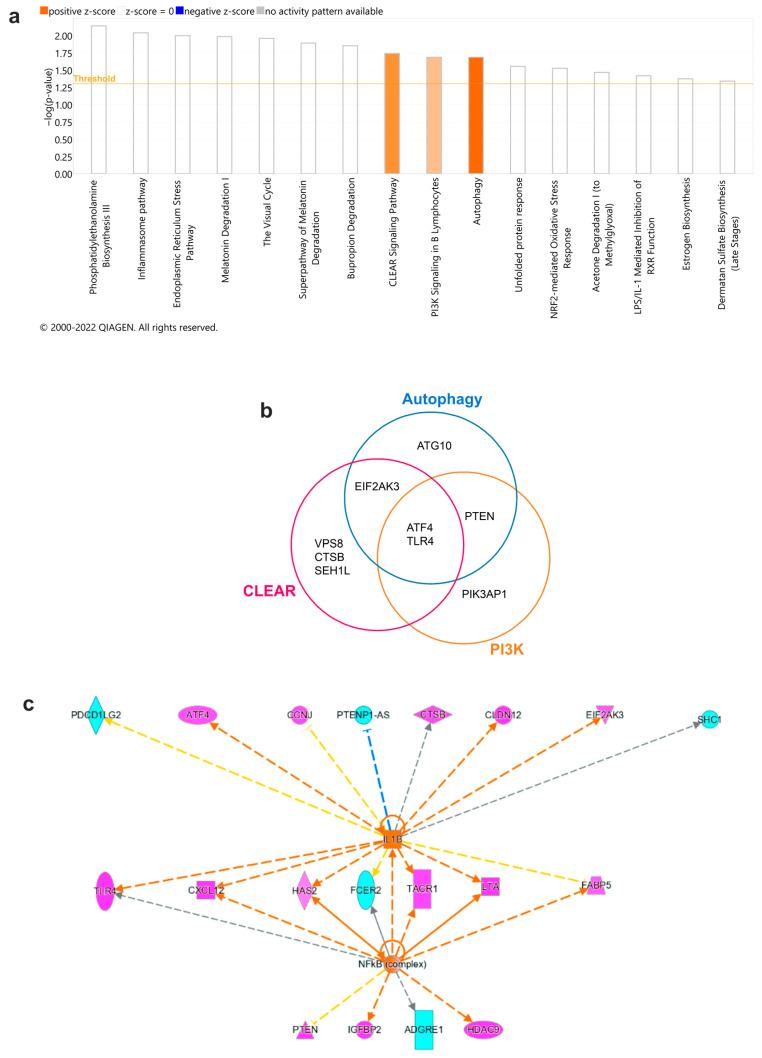
Canonical pathway (CP) analysis revealed functional enrichment of the CLEAR and Autophagy pathways in mast cells, with an upstream role for IL1B. Canonical pathway analysis was carried out on 203/249 differentially expressed genes (q < 0.05) using IPA. (**a**) CP analysis of DEGs, showing a Fisher exact test *p*-value < 0.05. Activation z-scores are displayed as orange for predicted activation. (**b**) Venn diagram of shared differentially-expressed genes identified in the CLEAR, Autophagy, and PI3K Signaling in B Lymphocytes (PI3K) canonical pathways. (**c**) Upstream regulator analysis of isolated MCs based on gene expression. Upregulated/downregulated genes (log2 fold change > 0/< 0) are respectively marked in magenta/cyan. Activation z-scores are displayed as orange arrows for predicted activation, blue for predicted inhibition, and grey for no prediction. Inconsistent findings are highlighted in yellow.

To interpret the transcriptomic analysis in the context of curated pathways and predict the underlying mechanisms, we performed canonical pathway and upstream regulator analyses using Ingenuity Pathway Analysis (IPA). Canonical pathway analysis of differentially expressed genes revealed enrichment in sixteen canonical pathways, among which three showed predicted activation, namely, Coordinated Lysosomal Expression and Regulation (CLEAR) Signaling Pathway, Autophagy, and PI3K Signaling in B Lymphocytes (Figure 4a). Common shared genes across the three identified pathways were *ATF4* and *TLR4* (Figure 4b). Upstream regulators analysis revealed a possible role of *IL1B* and *NFkB* in the modulation of differentially expressed genes (Figure 4c), including *ATF4*, *CTSB*, *CLD12*, *EIF2AK3*, *CXCL12*, *FABP5*, *LTA*, *TLR4*, and *TACR1*. Causal network analysis predicted the involvement of the IFN and IL-12 cytokine families, which promote downstream gene activation. These results suggest an influence of the surrounding pro-inflammatory cytokine milieu in the observed MC signature in psoriasis skin, in particular mediated by IL-1β.

**Figure 5:**
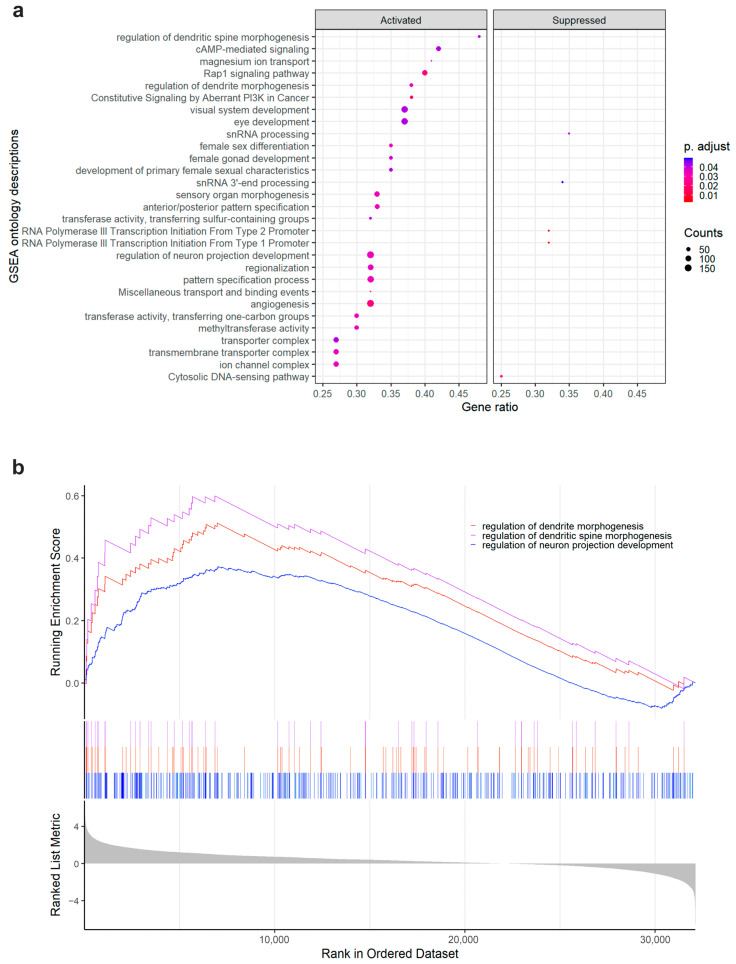
Neuron-associated ontology terms in psoriasis mast cells in gene set enrichment analysis. Gene set enrichment analysis of RNA isolated from MCs from healthy (*n* = 7) and psoriasis lesional skin (*n* = 6, 2 donors pooled). (**a**) Scatterplot of significantly enriched ontology terms obtained through the gseGO, gseKEGG, and gsePathway functions of ClusterProfiler/ReactomePA R tools. The cut-off for significance was set to the false discovery rate-corrected *p* value of < 0.05, and the analyses returned a total of 29 enriched ontology descriptions (23 GO, 2 KEGG, 4 REACTOME), as shown on the left of the plot. The colour of the dots represents the adjusted *p*-value, while the size of the dots represents the gene counts for each ontology term. The gene ratio is a measure of the total enrichment based on positive hits out of the total number of genes in that pathway. (**b**) Plot of enrichment scores and rank in the ordered dataset of the neuron-associated GO ontology terms GO:0048814, GO:0061001 and GO:0010975, with the highest-ranked genes showing the most enrichment.

Gene set enrichment analysis (GSEA) of ranked differentially expressed genes revealed significantly enriched genes (FDR *p* adjusted value < 0.05) in 29 pathway terms across three ontology databases (Gene Ontology, GO; Kyoto Encyclopedia of Genes and Genomes, KEGG; and REACTOME) in MCs from psoriasis compared to normal skin (Supplementary Table S5, Figure S5a). Among these, three (10.34%) were neuron-related pathways involved in the regulation of neuron projection development (GO:0010975, *p* = 2.53 × 10^−5^, FDR-padj = 0.0337), regulation of dendrite morphogenesis (GO:0048814, *p* = 5.08 × 10^−5^, FDR-padj = 0.0347), and regulation of dendritic spine morphogenesis (GO:0061001, *p* = 9.50 × 10^−5^, FDR-padj = 0.0422), respectively (Figure 5e). These results hint at MC engagement in neuronal development in psoriasis skin, supporting the evidence of close interaction between MCs and nerve fibres in lesions.

### 3.3. Mast Cell Signatures Are Unequally Represented in Psoriasis Whole Skin Studies

To assess the contribution of observed signatures in MCs isolated from psoriasis lesions, we sought to compare the differential gene expression in isolated MCs to the published whole-tissue microarray datasets GDS4602, GSE80047, and GSE78097 from psoriasis patients (Table 1).

**Figure 6:**
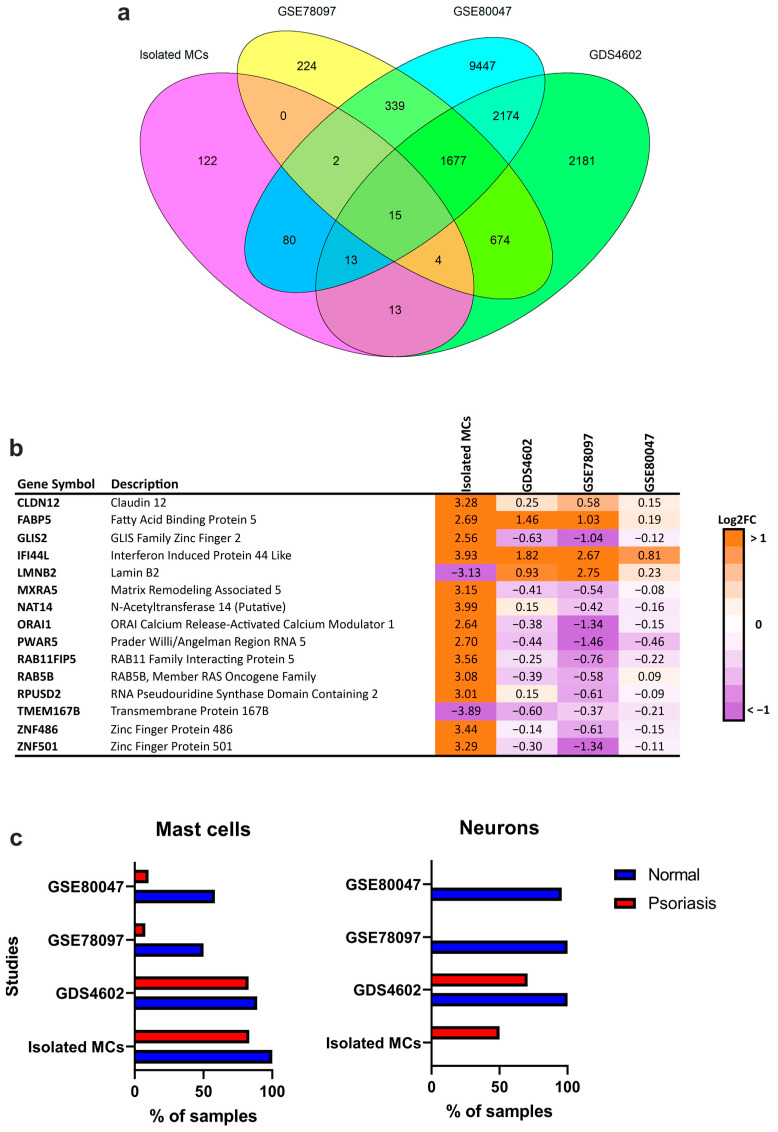
Psoriasis mast cells share half of their differentially expressed genes with whole skin, while mast cells and neuronal signatures are unequally represented across studies and conditions. (**a**) Venn diagram of the number of overlapping differentially expressed genes between healthy donors and psoriasis patients in isolated MCs and microarray datasets. (**b**) Log2 fold change compared to healthy controls of 15 overlapping genes across datasets. (**c**) Cell type enrichment analysis of publicly available datasets and of MCs isolated from normal skin and psoriasis lesions, performed using xCell. The proportion of samples in which significant (*p* < 0.05) MC and neuron enrichment was detected is displayed per study. Isolated MCs were added as internal control for MC-specific signatures.

Among the 249 genes identified in isolated MCs, 127 genes were shared with at least one of the selected microarray datasets (Figure 6a, Supplementary Table S6), with 15 genes shared by all datasets (Figure 6b). In particular, *CLDN12*, *FABP5*, *IFI44L*, and *TMEM167B* showed similar regulation patterns across datasets. Of note, *CLDN12* and *FABP5* were among the genes identified in IPA as being modulated by upstream *IL1B* and *NFkB* complex activity (Figure 4c).

We then performed canonical pathway and upstream regulator comparison analysis between the isolated MCs and whole-tissue datasets using IPA, and analysed the similarities in the enriched canonical pathways and upstream regulators found in the isolated MCs. We observed similar positive activation z-scores in the Autophagy and CLEAR pathways, as well as a downstream effect of *IL1B* (Supplementary Tables S7 and S8).

Comparison of causal network analysis showed shared predicted activation mediated by the IL-12 cytokine family only in GSE78097, while no predicted shared effect of the IFN cytokine family were observed (Supplementary Table S8).

In addition, GSEA analysis of whole-skin microarray studies was performed to assess any similarities in enrichment with isolated MCs. Among significantly enriched ontology terms, only four were shared with at least one dataset, namely, cytosolic DNA-sensing pathway, pattern specification process, anterior/posterior pattern specification, and regionalization (Supplementary Table S8).

Hence, both our IPA and GSEA analyses indicate that isolated MCs share marginal overlap of the pathways and gene set enrichment characteristics of the whole skin of psoriasis subjects.

To assess whether the discrepancies observed in IPA and GSEA could be the result of uneven representation of MCs in whole skin samples, we performed xCell enrichment, with a particular focus on the relative distribution of MC- and neuron-associated signatures in normal and involved skin samples. Isolated MCs were included as internal control.

xCell enrichment revealed that the representation of MCs in different datasets was unequal in normal skin samples of GDS4602 (89.1%), GSE80047 (58.3%), and GSE78097 (50%), and detected only in a small number of samples of involved psoriasis skin (10% and 7.7% of samples in GSE80047 and GSE78097, respectively) (Figure 6c). The same analysis showed that neuronal signatures in involved skin were undetectable in GSE80047 and GSE78097, while they were detected in 50% of isolated MC samples from involved psoriasis skin.

Hence, analysis of isolated MCs is necessary in order to define their specific signature in psoriasis, as it is underestimated in currently published whole skin studies. The reason for the low MC representation in involved psoriasis skin could be due to insufficient MCs in biopsy samples or to a change in the MC-specific signature defined by the xCell algorithm.

### 3.4. CTSB, TACR1, and TLR4 Are Itch-Associated Genes Differentially Expressed by Psoriasis Skin Mast Cells

To explore gene signatures contributing to itch in psoriasis, we compiled a curated list of 208 itch-associated genes after a systematic review of the available literature on genes known to be either positively correlated with perceived itch or scratching, to directly induce itch, or to induce sensitisation to pruritogens (Supplementary Table S9).

To investigate whether these genes were upregulated in psoriasis skin, and as such could contribute to psoriatic itch, their differential expression was investigated in published microarray datasets. Of itch-associated genes, 89 (42.7%), 135 (64.9%), and 42 (20.2%) were significantly differentially expressed in GDS4602, GSE80047, and GSE78097, respectively (Supplementary Table S10). However, in the isolated MCs only three itch-associated genes (1.4%) were statistically different from healthy controls, namely, *CTSB*, *TACR1*, and *TLR4*. These genes were only found to be differentially expressed in the GSE80047 dataset, with *CTSB* and *TLR4* expression significantly upregulated and *TACR1* significantly downregulated (Supplementary Table S10).

Taken together, these data imply that involved psoriasis skin MCs are primed to respond to selective immune and neurogenic factors as well as to release proteases that can activate itch responsive neurons but lack the effect mediated by the majority of literature-defined itch-associated genes and variably represented in whole skin analyses.

### 3.5. Cathepsin B Is a Predictive Indicator of Mast Cell-Dependent Itch in Psoriasis

To identify itch-associated genes that are predictive indicators of psoriasis in comparison with normal skin, random forest analysis was utilised.

**Figure 7:**
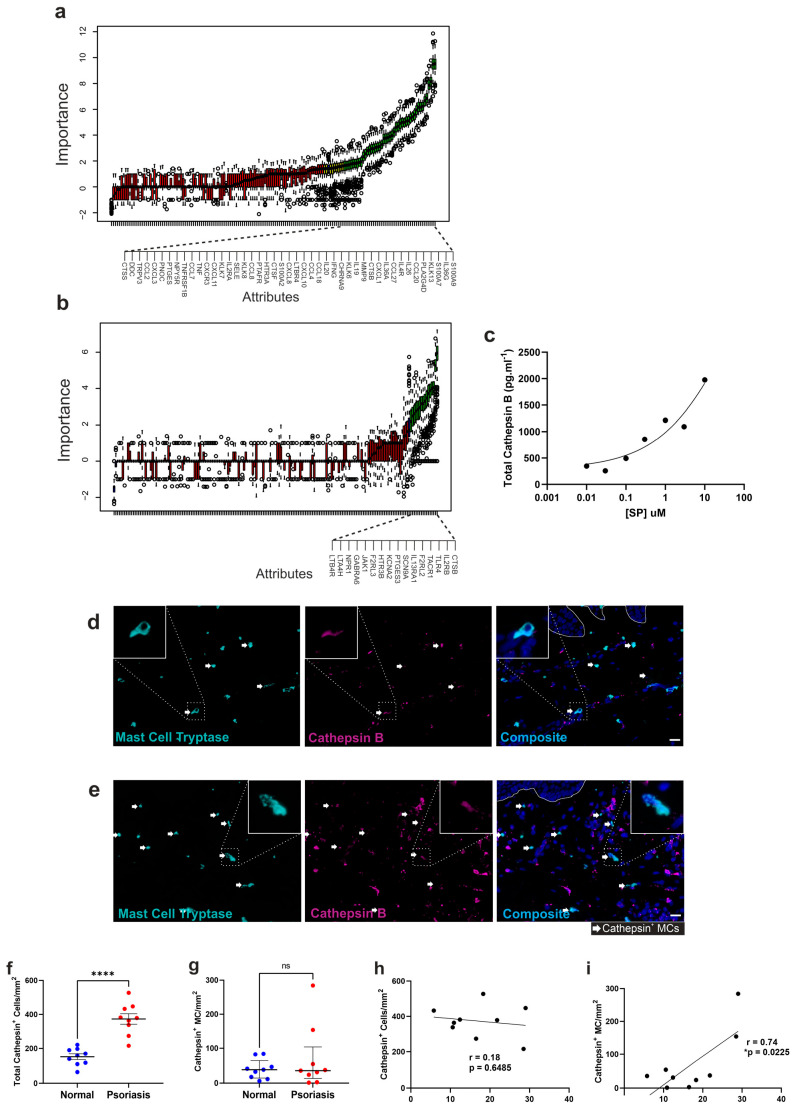
Random forest analysis of itch-associated genes and cathepsin B expression in mast cells. Random forest plot of differentially expressed genes in (**a**) psoriasis whole skin and (**b**) isolated MCs. Attributes that were significant (green bars), tentative, (yellow bars), or rejected (red bars) as indicative of the psoriasis condition are listed in order of importance. Worst, average and best shadow in each iteration are shown (blue bars). Gene names of significantly indicative attributes are enlarged below each plot. (**c**) Cathepsin B measured in the cell-free supernatant from human MCs stimulated with SP (concentrations shown) for 1 h. (**d**,**e**) Representative photomicrographs of the dermis of healthy (**d**) and psoriasis skin (**e**), showing cathepsin B (magenta) and MC tryptase (cyan) with nuclei stained with DAPI. Scale bar = 20 µm. Cathepsin B+ MCs are indicated by arrows. Examples of cathepsin-stained mast cells have been enlarged in each panel. (**f**,**g**) Number of total cathepsin B+ cells (**f**) and cathepsin B+ MCs (**g**) in normal skin (blue dots) and psoriasis skin (red dots). Data are mean± SEM (**f**) and median± IQR (**g**) of *n* = 9 donors. **** = *p* < 0.0001, ns: not significant (unpaired *t*-test). (**h**,**i**) total number of cathepsin B+ cells and cathepsin B+ MCs in psoriasis skin correlated with PASI. Significant positive correlation indicated by * *p* = 0.0225 (Pearson correlation).

Random forest analysis found 45 itch-associated genes differentially expressed in psoriasis whole skin to be significant indicators of the psoriasis condition (training set AUC OOB = 1, OOB Error rate = 0%, validation set AUC = 1, 95% CI = 0.934–1; Figure 7a). Meanwhile, this was the case for 16 of the itch-associated genes in MCs isolated from psoriasis skin, including *CTSB*, *TLR4*, and *TACR1* (training set AUC OOB = 1, OOB Error rate = 11.11%, validation set AUC = 0.875, 95% CI = 0.63–1; Figure 7b).

*CTSB*, *JAK1*, and *LTB4R* were the only genes found by random forest analysis to be significant indicators of the psoriasis condition in both the whole skin datasets and isolated MCs. *CTSB* was the only gene to be significantly upregulated in MCs as well, suggesting that Cathepsin B could serve as common indicator of MC-dependent itch signature in psoriasis.

Cathepsin B is upregulated in animal models of inflamed skin, where it contributes to aberrant keratinocyte proliferation [90,91]. We demonstrated in vitro that cathepsin B is released by human peripheral blood-derived MCs in response to stimulation by neuropeptide SP (Figure 7c). Therefore, we investigated changes in cathepsin B expression in psoriasis skin via immunofluorescence microscopy. Cathepsin B was widely expressed in the epidermis and dermis of normal and psoriasis skin, including in MCs (Figure 7d,e and Supplementary Figure S4a,b). Total cathepsin B+ cells (mean frequency of 151.4 ± 16.61/mm^2^ vs. 373.3 ± 30.93/mm^2^ (mean ± SEM) **** *p* < 0.0001) were found to be significantly increased in psoriasis skin. Although cathepsin B+ MC density was not significantly increased (37.85 (14.42–63.96) vs. 35.21 (13.20–103.8) (Median (IQR)) (Figure 7g), the density of cathepsin B+ MCs did significantly correlate with psoriasis severity (r = 0.74, * *p* = 0.0225), whereas the total cathepsin B+ cell number did not. Notably these analyses confirmed our earlier findings (Figure 1) in which the total MC number was increased in psoriasis skin and their density correlated with PASI (Supplementary Figure S4c,d). Our analyses did not include the measurement of acellular cathepsin B. However, we detected an increase of extracellular cathepsin B in the form of small spots, indicative of its induced release, which was present in increasing amounts in high-PASI subjects (Supplementary Figure S4e). These findings indicate that an increase in cathepsin B specific to MCs in psoriasis might provide functional cross-talk between peptidergic neurons and MCs.

## 4. Discussion

In this study, we sought to better define the involvement of MCs in psoriasis and the potential role of neuroimmune interactions in the pathogenesis of psoriatic itch. To elucidate the contribution of MCs in the generation of itch and explore the potential role of the cross-talk between MCs and neurons, we analysed the transcriptomic landscape of MCs isolated from psoriatic skin and assessed MC–neuron proximity through immunohistochemistry.

Here, we demonstrate increased MC and nerve fibre density which correlates with disease severity in psoriasis and that MCs and CGRP+ nerve fibres are spatially associated. Furthermore, we found that *CTSB* expression can serve as an indicator of MC involvement in the itch signature of psoriasis, and that MC cathepsin B expression correlates with psoriasis severity.

Mast cells were found to be significantly more frequent in psoriasis, which corroborates previous findings [23]. Further, we confirmed that an increased number of MCs and nerve fibres normalised to a specific area is associated with psoriasis severity. The association between the intensity of pruritus and density of MCs remains controversial [92,93], and it is possible that increased numbers alone could increase association between MC and nerves and increase the cutaneous innervation and neuropeptides content in lesions correlated with both disease and pruritus [92,94]. Interestingly, involved psoriasis skin from patients with pruritus shows increased MC activation, implying a mechanistic contribution of secreted MC mediators to itch [92].

Close proximity between mucosal MCs and intrinsic CGRP+ sensory neurons has been demonstrated in the mouse colon, where the interaction causes MC activation [95], as well as in psoriasis [6]. In human skin, CGRP is present in few sensory nerve fibres. However, CGRP+ nerve fibres are increased in conditions such as prurigo nodularis, and are thought to contribute to neurogenic inflammation and MC recruitment [96]. The pruriceptive neuronal subtype NP2 is known to express CGRP in mice [97]. Whether CGRP release in psoriasis is fundamental to MC activation and immune cell recruitment, and consequently the initiation of the acute inflammatory process [98], or whether tryptase release by MCs is key in deactivating CGRP [99], thereby promoting resolution of the lesion, is yet to be defined.

The classical MC mediator involved in itch in a variety of inflammatory diseases is histamine [100]. However, it seems to play a minor role in psoriatic pruritus [44]. According to our findings, MCs derived from psoriasis lesions express higher levels of genes involved in the regulation of neurons, and in particular, receptors that could facilitate communication with sensory nerves, such as the substance P receptor (*TACR1*). This indicates that the psoriatic microenvironment alters the functionality of skin MCs. Whether these changes lead to increased susceptibility of these cells to neuropeptides remains to be defined. Furthermore, the inflammatory indicators found to be upregulated in psoriasis MCs, such as *ATF4*, *CXCL12*, *LTA*, *TACR1*, *TLR4*, and *CTSB*, interestingly share IL-1β as a common modulator. This cytokine has a key role in psoriasis [101,102], and its abnormal regulation has been suggested to represent one of the early steps in the pathogenesis of the disease [103,104]. Thus, our findings suggest that MCs are challenged early on by the cytokine milieu of psoriatic epidermal keratinocytes.

Substance P is known to ligate both the MC Tachykinin receptor 1 (TACR1), sometimes called neurokinin 1 receptor (NK1R), and MRGPRX2 [105,106,107]. In our study, *TACR1* was upregulated in psoriasis MCs, while *MRGPRX2* displayed no significant changes in its expression. While these findings could indicate a prominent role of TACR1 in MC-dependent itch in psoriasis, we cannot exclude the contribution of the MRGPRX2-induced signalling pathway, as this receptor is known to be constitutively and preferentially highly expressed in skin MCs [108,109].

Activation of MCs via TLR4 engagement induces the release of pro-inflammatory cytokines [110]. Furthermore, TLR4 antagonists administered in a rat model of chronic relapsing itch alleviated both itch and chronic dermatitis [111]. Thus, the increased expression of TLR4 could indicate an altered susceptibility of MCs to endogenous ligands such as human beta-defensin 2, which has been shown to not only promote itch through TLR4 signalling in mice [112], but to be overexpressed in psoriasis skin [113].

Interestingly, Th2 cytokines such as IL-4 and IL-13, expressed by MCs, and suggested as signalling bridges between immune sense and nerve fibres [114,115], were not modulated in MCs in psoriasis. Indeed, our analysis of itch-associated genes indicated a specific MC signature in psoriasis and a lack of regulation in the majority of the literature-defined itch-associated genes. Whether additional MC-specific signalling pathways are contributing to itch in psoriasis remains to be investigated.

In our random forest analyses, *CTSB* was the only gene common to the psoriasis whole-skin datasets and isolated MCs which was a significant indicator of the psoriasis condition. Furthermore, we observed an increase in cathepsin B expression in psoriasis skin, an increase in the density of cathepsin B+ MCs correlating to PASI, and cathepsin B protease release from MCs in response to SP activation. Cathepsin B is a protease with pro-inflammatory functions that is highly expressed in psoriasis plaques [91,116,117]. It has been suggested that this may contribute to aberrant keratinocyte growth and differentiation [118]. Furthermore, *CTSB* is considered to be an established itch-associated gene [35]. Cathepsin B localises to the secretory granules of primary human skin-derived MCs, and is required for the activation of pro-tryptase into active tryptase [119]. MCs have been suggested to contribute to psoriatic pruritus by releasing tryptase [54,120]. Because human tryptase is a PAR-2 ligand, the significant upregulation of *CTSB* in MCs in psoriasis lesions and the correlation of cathepsin B with PASI would suggest a contribution to promoting PAR-2 signalling in sensory nerve fibres. While the downregulation of cathepsin B in keratinocytes has been shown to reduce their proliferation and inflammatory response [90], the silencing of *CTSB* expression in MCs could become a means of controlling MC-induced itch in psoriasis.

Interestingly, neuronal loss and brain atrophy has been observed in mice lacking *CTSB*, suggesting a role for cathepsin B in neuronal maintenance [121]. However, whether this MC protease plays a role in promoting the increase and outgrowth of nerve fibres in psoriasis is yet to be investigated.

In summary, our findings suggest that bidirectional cutaneous cross-talk between MCs and nerve fibres regulates the growth, morphogenesis, and mediator/receptor expression of the latter. However, the altered microenvironment of neuropeptide, cytokine, and anti-microbial mediators in psoriasis promotes a unique psoriasis-specific itch signature in MCs. In this context, cathepsin B could serve as a biomarker and candidate for interventional therapy.

## Figures and Tables

**Table 1:** Summary of differential gene expression results from isolated mast cells and whole skin microarray datasets of psoriasis patients.

Study	Source	Technique	Total Genes (*n*.)	DEGs *p* < 0.05 (*n*.)	DEGs q < 0.05 (*n*.)	Upregulated q < 0.05 (*n*.)	Downregulated q < 0.05 (*n*.)
Isolated MCs	Skin derived MCs	RNA-seq	32,119	5924	249	192	57
GDS4602	Bulk skin	Microarray	22,190	14,315	6751	4234	2517
GSE78097	Bulk skin	Microarray	22,190	13,393	2935	1600	1335
GSE80047	Bulk skin	Microarray	21,915	14,436	13,747	7109	6638

Mast cells (MCs) were isolated from healthy donors (*n* = 7) and involved skin of psoriasis patients (*n* = 6, 2 donors pooled). RNA was extracted and analysed. Microarray data from three separate studies on bulk skin from healthy donors and psoriasis patients were downloaded from the Gene Expression Omnibus database. Two-sample comparison analysis was performed for each study comparing normal and involved samples. Differentially expressed genes (DEGs) were selected based on *p*-values adjusted for the false discovery rate (q < 0.05).

## Data Availability

In addition to the datasets used above [66,67,68], the hMC RNAseq dataset discussed in this publication has been deposited in NCBI’s Gene Expression Omnibus and can be found through the GEO series accession number GSE217060 at https://www.ncbi.nlm.nih.gov/geo/query/acc.cgi?acc=GSE217060, (Atmoko et al., 2023).

## Supplementary Materials

The following supporting information can be downloaded at: https://www.mdpi.com/article/10.3390/cells12172177/s1, Figure S1: Substance P density, spatial association with MCs and correlation to severity of psoriasis; Figure S2: Vasoactive intestinal peptide density, spatial association with MCs and correlation to severity of psoriasis; Figure S3: Mast cell isolation and gating strategy; Figure S4: Colocalisation of cathepsin B and tryptase in mast cells in healthy and psoriasis skin; Figure S5: Single channel images of composite images shown in other figures; Table S1: Spatial association of MCs and nerve fibres in normal and lesional psoriasis skin; Table S2: Immunohistochemistry and MC isolation subject demographics; Table S3: MC isolation subject itch characteristics; Table S4: List of differentially expressed genes by mast cells isolated from involved psoriatic skin compared to normal skin; Table S5: List of significant ontology terms across different ontology databases from gene set enrichment analysis (GSEA); Table S6: Complete list of shared differentially expressed genes (DEGs) shared across different datasets; Table S7: Enriched canonical pathways, upstream regulators and causal networks identified in isolated MCs by IPA; Table S8: Enriched canonical pathways, upstream regulators and causal networks identified in published microarray datasets by IPA; Table S9: List of itch-associated genes from published literature; Table S10: Expression of itch-associated genes in mast cells isolated from involved psoriatic skin and their expression in datasets GDS4602, GSE80047, and GSE7809.

## Abbreviations

CGRP	Calcitonin Gene-Related Peptide
DRG	Dorsal Root Ganglion
hMCs	Human Mast Cells
MCs	Mast Cells
SP	Substance P
VIP	Vasoactive Intestinal Peptide
DEGs	Differentially Expressed Genes
PASI	Psoriasis Area Severity Index
