# Supplementary material for: Human Mast Cells Upregulate Cathepsin B, a Novel Marker of Itch in Psoriasis
Source: Cells. 2023 Aug 30;12(17):2177. doi: 10.3390/cells12172177 (PMC10486964; doi:10.3390/cells12172177)
Supplement: Supplementary file 1 [file cells-12-02177-s001.zip › cells-2480642-supplementary.pdf]

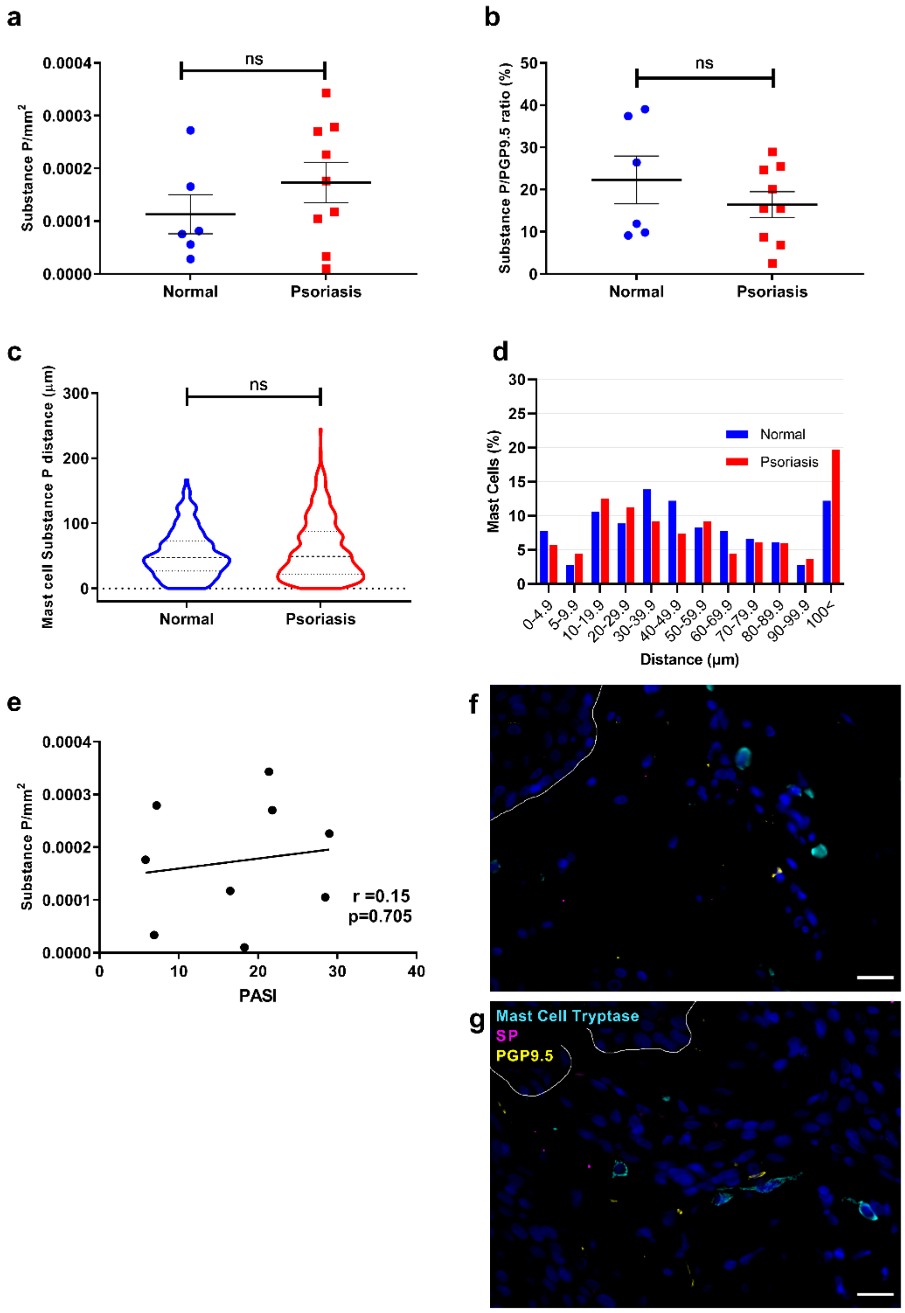

Supplementary Figure S1. Substance P density, spatial association with MCs and correlation to severity of psoriasis. Immunohistochemistry was used to identify MCs (tryptase staining), nerve fibres (PGP 9.5 staining) and substance P (SP staining) in normal skin (n = 6) and involved psoriasis skin (n=9) in 5µm paraffin sections. SP density **(a)** and the ratio of SP positive neuro fibres **(b)** are not significantly different in involved psoriasis skin compared to normal skin. Distance between MCs and SP positive nerve fibres is not significantly different in the skin of psoriasis and healthy individuals **(c)**. Frequency distribution of MCs and SP distance **(d)**. Pearson correlation analysis for SP density and severity index (PASI) **(e)**. **(f,g)** Representative photomicrographs of normal (f) and involved (g) skin show MC tryptase (cyan) PGP9.5 (yellow) and SP (magenta). Scale bar 20µm. Unpaired t-test (a,b), Mann-Whitney U test (c), ns = not significant.

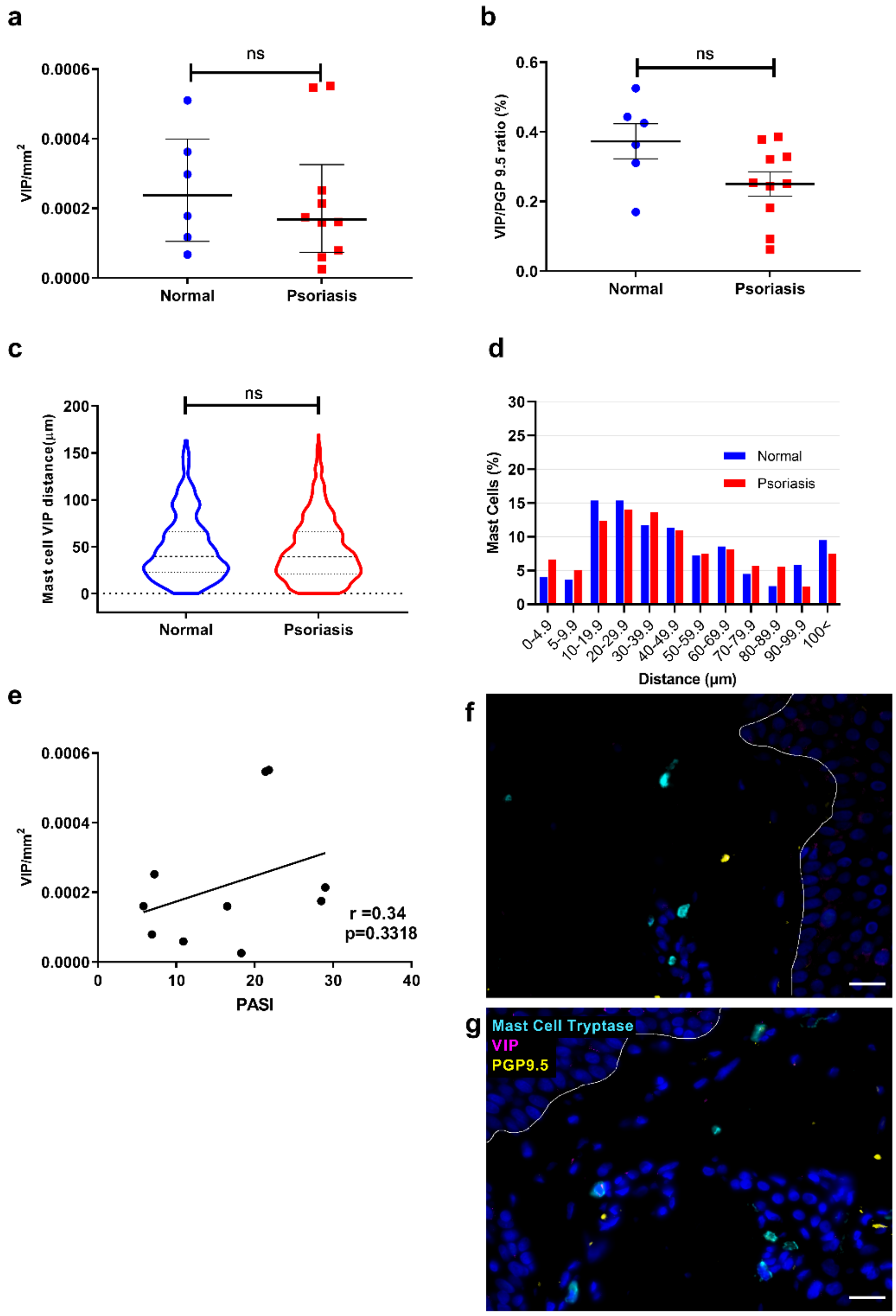

Supplementary Figure S2. Vasoactive intestinal peptide density, spatial association with MCs and correlation to severity of psoriasis. Immunohistochemistry was used to identify MCs (tryptase staining), nerve fibres (PGP 9.5 staining) and Vasoactive intestinal peptide (VIP staining) in normal skin (n = 6) and involved psoriasis skin (n=10) in 5µm paraffin sections. VIP density **(a)** and the ratio of VIP positive neuro fibres **(b)** are not significantly different in involved psoriasis skin compared to normal skin. The distance between MCs and VIP positive nerve fibres is not significantly different in the skin of psoriasis and healthy individuals **(c)**. Frequency distribution of MCs and VIP distance **(d)**. Pearson correlation analysis for VIP density and severity index (PASI) **(e)**. **(f,g)** Representative photomicrographs of normal (f) and involved (g) skin show MC tryptase (cyan) PGP9.5 (yellow) and SP (magenta). Scale bar 20µm. Mann-Whitney U test (a, c). Unpaired t-test (b), ns = not significant.

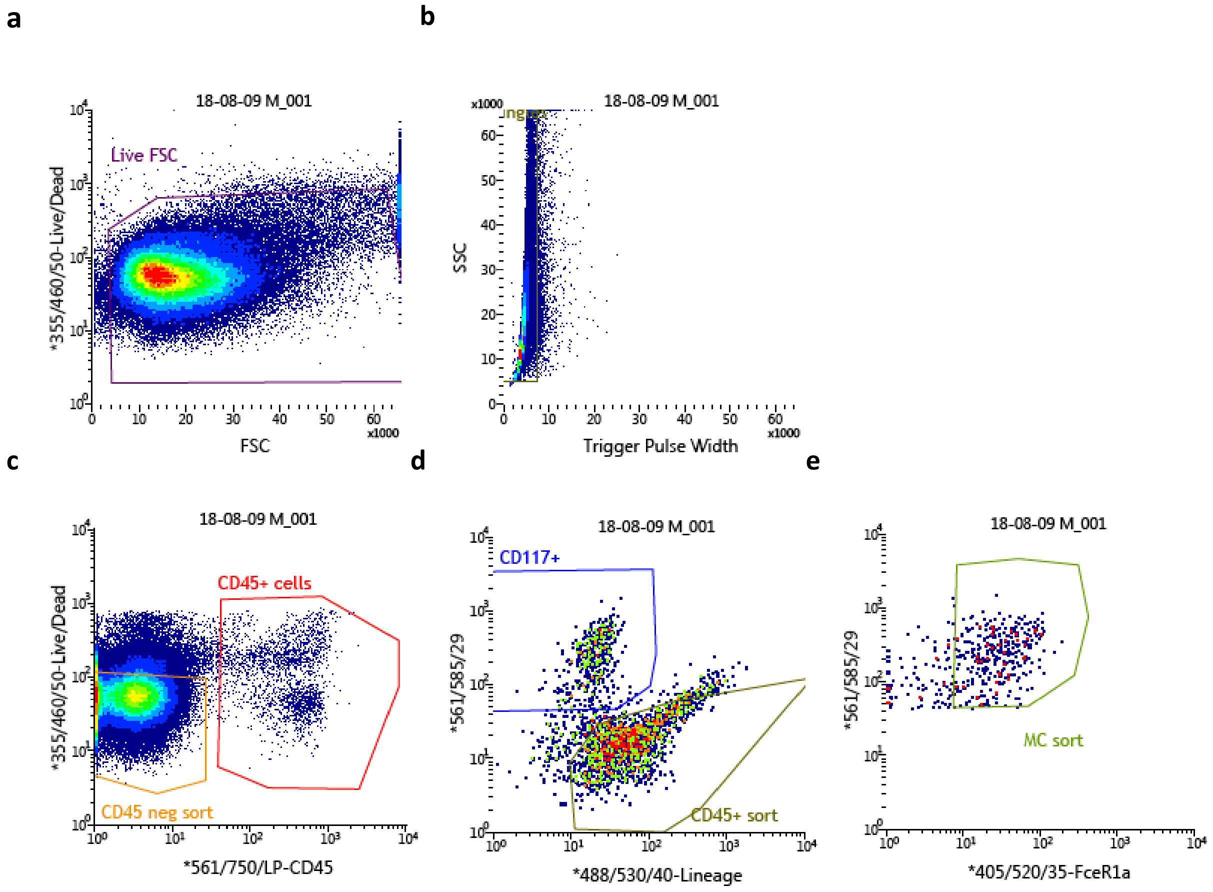

Supplementary Figure S3. Mast cell isolation and gating strategy. **(a)** Exclusion of dead cells by Live/Dead staining. **(b)** Exclusion of doublets by trigger pulse width. **(c)** Cells divided into two gated populations, CD45+ and CD45- cells for sorting. (**d)** CD45+ cells divided into Lineage+ cells for CD45+ sorting, and Lineage- CD117 high cells. **(e)** Lineage- CD117 high FcεRIα+ cells gated for MC sorting.

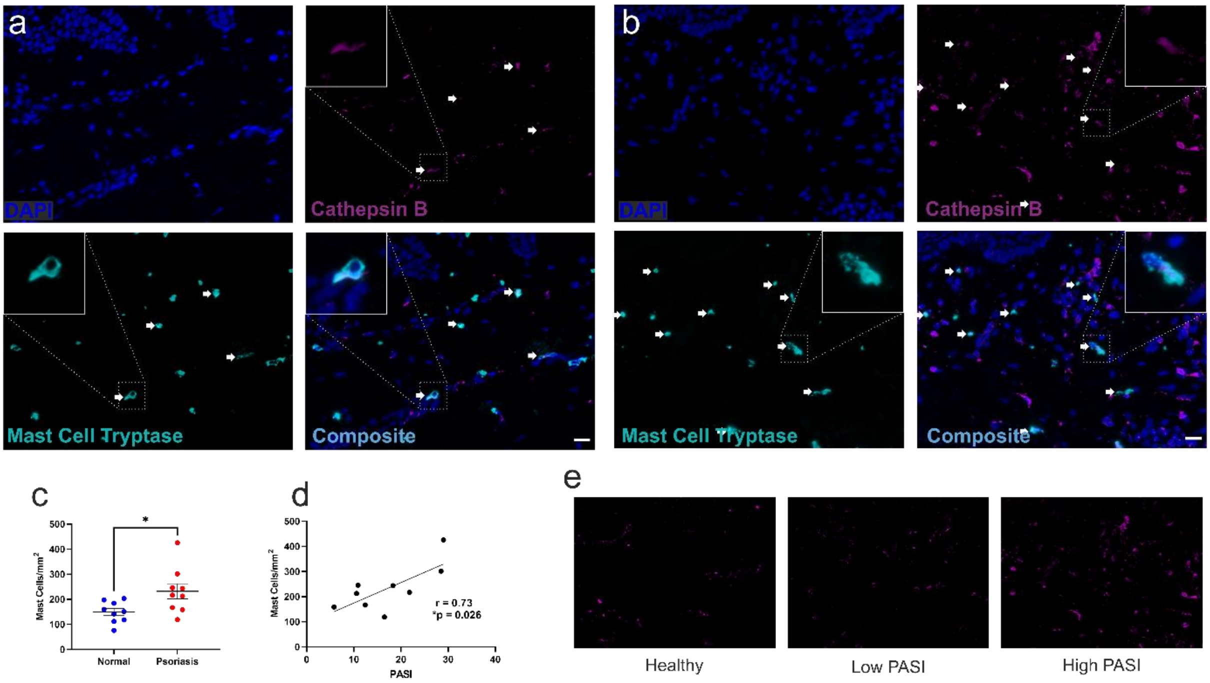

Supplementary Figure S4. Colocalisation of cathepsin B and tryptase in mast cells in healthy and psoriasis skin. (**a**) DAPI (blue), cathepsin B (magenta) and MC tryptase (cyan) immunofluorescence in skin from a healthy volunteer showing MCs containing cathepsin B (arrows). Upper left inlay shows enlarged section with cathepsin stained MC. (**b**) DAPI (blue), cathepsin B (magenta) and MC tryptase (cyan) immunofluorescence in involved skin from a psoriasis patient showing MC containing cathepsin B (arrows). Upper right inlay shows enlarged section with cathepsin stained MC. (**c**) Number of MCs per area in normal and involved psoriasis skin from patient samples used for cathepsin B staining. Data are mean±SEM of n=9 donors in each group (* = $p < 0.05$ (unpaired t-test). (**d**) Number of MCs correlated with psoriasis severity index (PASI) ((* = $p < 0.05$ (Pearson correlation)). (**e**) Cathepsin staining in healthy, low PASI, and high PASI individuals showing greater acellular cathepsin staining with high PASI.

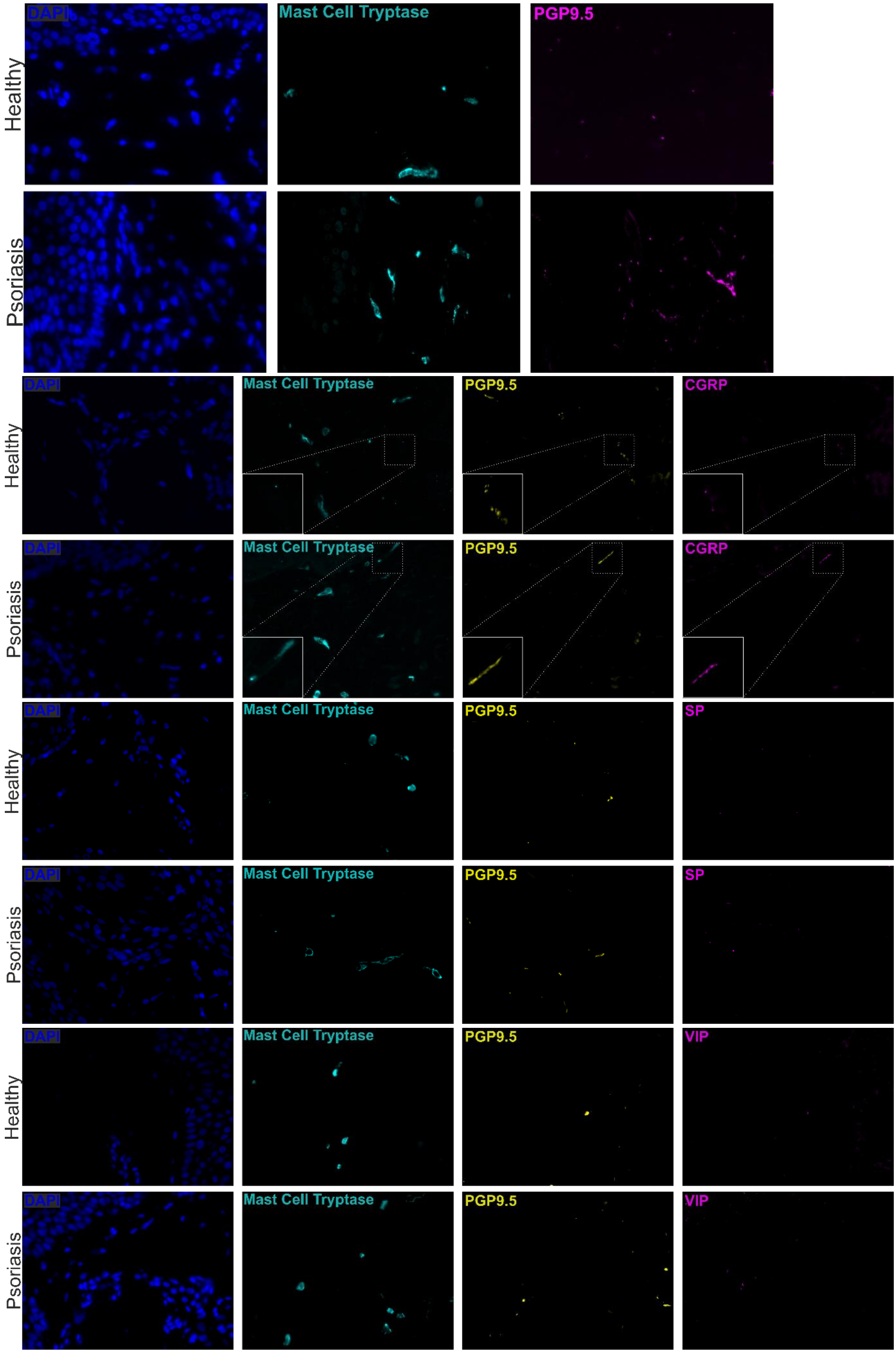
DAPI
Mast Cell Tryptase
PGP9.5
Healthy
Psoriasis
CGRP
SP
VIP

Supplementary figure S5. Single channel images of composite images shown in other figures. Single channel images of composite images from figure 1 showing DAPI (blue), mast cell tryptase (cyan) and PGP9.5 (magenta), from figure 2 showing DAPI (blue), mast cell tryptase (cyan), PGP9.5 (yellow) and CGRP (magenta), from supplementary figure 1 showing DAPI (blue), mast cell tryptase (cyan), PGP9.5 (yellow) and SP{ (magenta) and from supplementary figure 2 showing DAPI (blue), mast cell tryptase (cyan), PGP9.5 (yellow) and VIP (magenta). All images are at same scale as labelled in original figures.

Supplementary Table S1. Spatial association of MCs and nerve fibres in normal and lesional psoriasis skin. Dual immunohistochemistry was used to identify dermal MCs (tryptase) and nerve fibres (PGP 9.5) in normal (n = 8) and involved psoriasis skin (n=11) in 5μm paraffin sections. Ten areas of interest (AOI) per tissue section were analysed and shortest distance between MCs and neuro fibre was measured. MCs showed significantly increased proximity to nerve fibres in involved psoriasis dermis compared to normal skin (****p<0.0001, see also Figure 1a-h).

a

Subject	Group	MC Density (MC/mm^2)	PGP9.5 Density ($PGP9.5/mm_2$)	PASI
1	Normal	110.67	0.003183	N/A
2	Normal	195.77	0.002010	N/A
3	Normal	152.60	0.001214	N/A
4	Normal	158.49	0.002671	N/A
5	Normal	188.72	0.000838	N/A
6	Normal	135.15	0.000443	N/A
7	Normal	105.62	0.000117	N/A
8	Normal	89.56	0.002552	N/A
9	Psoriasis	159.96	0.004140	16.5
10	Psoriasis	292.73	0.004867	10.6
11	Psoriasis	225.45	0.007866	21.4
12	Psoriasis	430.23	0.004930	10.9
13	Psoriasis	250.25	0.002672	5.8
14	Psoriasis	551.69	0.008711	29
15	Psoriasis	298.37	0.005481	18.3
16	Psoriasis	498.27	0.007731	28.5
17	Psoriasis	157.86	0.004598	6.9
18	Psoriasis	377.13	0.007368	21.8
19	Psoriasis	193.14	0.003752	7.2

b

MC/PGP9.5	Healthy		Lesional	
mean±SEM(μm)	35.5±1.65		22.2±0.66	
Distance (μm)	No. MCs	%	No. MCs	%
**0-4.9**	44	**11.8%**	198	**19.9%**
**5-9.9**	37	**9.9%**	141	**14.2%**
**10-19.9**	69	**18.5%**	240	**24.1%**
**20-29.9**	49	**13.2%**	156	**15.7%**
**30-39.9**	42	**11.3%**	92	**9.3%**
**40-49.9**	44	**11.8%**	67	**6.7%**
**50-59.9**	26	**7.0%**	30	**3.0%**
**60-69.9**	15	**4.0%**	34	**3.4%**
**70-79.9**	7	**1.9%**	15	**1.5%**
**80-89.9**	9	**2.4%**	8	**0.8%**
**90-99.9**	8	**2.2%**	7	**0.7%**
**>100**	22	**5.9%**	6	**0.6%**
N	**372**		**994**	

c

MC/PGP9.5	Healthy								
Distance (μm)	1	2	3	4	5	6	7	8	N
**0-4.9**	10	9	5	3	6	5	6	0	44
**5-9.9**	4	5	6	7	6	7	1	1	37
**10-19.9**	7	12	11	11	11	7	7	3	69
**20-29.9**	7	11	7	3	10	2	0	9	49
**30-39.9**	3	4	5	4	11	5	4	6	42
**40-49.9**	3	6	6	6	9	7	3	4	44
**50-59.9**	2	6	4	3	5	4	2	0	26
**60-69.9**	2	1	3	5	2	1	0	1	15
**70-79.9**	0	0	1	0	0	5	0	1	7
**80-89.9**	0	2	0	2	0	0	3	2	9
**90-99.9**	1	1	1	2	0	1	2	0	8
**>100**	3	6	2	5	0	1	3	2	22
MCs/Subject	42	63	51	51	60	45	31	29	

d

MC/PGP9.5	Lesional											
Distance (μm)	9	10	11	12	13	14	15	16	17	18	19	N
**0-4.9**	4	26	15	16	5	48	11	35	2	24	12	198
**5-9.9**	5	17	8	17	8	28	9	21	6	17	5	141
**10-19.9**	18	20	12	26	13	46	17	39	10	32	7	240
**20-29.9**	5	12	14	26	13	15	17	19	8	14	13	156
**30-39.9**	5	8	6	17	11	8	5	10	7	12	3	92
**40-49.9**	4	5	9	10	8	9	5	8	4	1	4	67
**50-59.9**	3	2	2	7	5	2	2	2	2	0	3	30
**60-69.9**	3	3	3	11	8	0	2	0	2	0	2	34
**70-79.9**	0	1	2	2	4	1	1	1	1	1	1	15
**80-89.9**	1	0	0	0	5	1	0	0	0	0	1	8
**90-99.9**	1	0	1	0	3	0	0	0	1	0	1	7
**>100**	0	0	2	2	2	0	0	0	0	0	0	6
MCs/Subject	49	94	74	134	85	158	69	135	43	101	52	

Supplementary Table S2. Immunohistochemistry and MC isolation subject demographics.

	Neuronal Immunohistochemistry			Mast cell isolation			Cathepsin Immunohistochemistry		
	**Psoriasis**	**Healthy**	**p value**	**Psoriasis**	**Healthy**	**p value**	**Psoriasis**	**Healthy**	**p value**
**N**	11	8		7	7		9	9	
**Age (mean ± SD)**	48 ± 18	48 ± 17	>0.9999	57 ± 21	46 ± 24	0.3794	53 ± 20	44 ± 15	0.2962
**Male:female**	05:06	04:04	>0.9999	02:05	05:02	0.29	05:04	03:06	0.6372

Significance values calculated using unpaired t-test (Age) or Fisher exact (Male:Female), significance set at $p < 0.05$

Supplementary Table S3. MC isolation subject itch characteristics.

	PASI	Itch rating	Worst itch in 24 hours rating
**Lesional 1**	8.2	5	5
**Lesional 2a (pooled)**	7.5	0	0
**Lesional 2b (pooled)**	5.8	0	2
**Lesional 3**	18.3	6	10
**Lesional 4**	28.5	3	3
**Lesional 5**	10.9	9	9
**Lesional 6**	12.4	1	6

Supplementary Table S4. List of differentially expressed genes by mast cells isolated from involved psoriatic skin compared to normal skin

Significantly upregulated genes

Gene symbol	Log2FC	p value	q value
DDI2	5.18	4.66E-05	0.0245
LMOD2	4.45	2.01E-05	0.0158
PRDM2	4.42	1.62E-06	0.0074
DCAF4L1	4.32	2.95E-05	0.0179
HS1BP3	4.22	1.26E-06	0.0072
RAD51D	4.21	5.81E-06	0.0093
LTA	4.16	0.000177	0.0373
ILDR2	4.07	0.000223	0.0422
BPTF	4.02	0.00025	0.0432
NAT14	3.99	1.07E-05	0.0132
NCMAP	3.98	3.69E-06	0.0080
IFI44L	3.93	1.57E-05	0.0153
CNNM2	3.92	0.000166	0.0357
AC090114.2	3.87	0.000278	0.0438
PRDM4	3.84	4.22E-05	0.0226
HDAC9	3.83	2.26E-05	0.0158
PIGN	3.83	2.45E-05	0.0161
IFNAR1	3.81	0.000101	0.0301
RPS6KL1	3.79	0.000277	0.0438
CXCL12	3.76	6.18E-06	0.0093
UQCRB	3.73	0.00025	0.0432
CCDC113	3.71	1.34E-06	0.0072
MS4A2	3.71	2.19E-05	0.0158
NSUN5P1	3.64	1.98E-06	0.0080
TLR4	3.59	0.000351	0.0483
TMEM17	3.57	6.91E-07	0.0072
AP5M1	3.56	0.000269	0.0438
RAB11FIP5	3.56	9.41E-05	0.0301
FAM46B	3.56	0.000373	0.0491
AC009078.2	3.53	0.000157	0.0355
CLK4	3.53	0.000243	0.0427
RTL1	3.52	9.28E-05	0.0301
PTEN	3.51	0.000182	0.0379
IFT57	3.48	1.29E-06	0.0072
GTPBP10	3.48	0.00031	0.0465
PACRG-AS3	3.48	2.94E-06	0.0080
ARHGAP20	3.47	0.000121	0.0327
NHLH2	3.47	0.000107	0.0301
RANBP2	3.47	2.92E-05	0.0179
FAM83E	3.47	2.04E-05	0.0158
KRTAP4-4	3.46	0.000271	0.0438
CYP2U1	3.45	4.01E-05	0.0219
ELAC2	3.45	7.06E-05	0.0283
ZNF486	3.44	0.000187	0.0379
RBM22	3.44	6.3E-06	0.0093
RPAP2	3.43	0.000374	0.0491
GLDN	3.43	0.000352	0.0483
AL008638.3	3.43	0.000339	0.0482
POTEC	3.42	0.000138	0.0331
PHYKPL	3.42	8.23E-05	0.0294
CASC17	3.40	5.81E-06	0.0093
MALAT1	3.39	0.000234	0.0425
AC007424.1	3.38	6.04E-05	0.0250
OR10Z1	3.37	0.000124	0.0327
UBXN4	3.36	0.000119	0.0325
RCC2P3	3.35	2.75E-05	0.0173
SUSD2	3.35	3.38E-05	0.0201
LRP2	3.35	0.000229	0.0424
PRKCQ-AS1	3.34	0.000299	0.0462
SLC35B2	3.32	0.00032	0.0472
GDAP2	3.31	9.68E-05	0.0301
GPR3	3.31	7.14E-05	0.0283
ABCA10	3.31	0.000342	0.0482
AL160286.3	3.30	1.72E-05	0.0155
ACSM2A	3.29	2.42E-05	0.0161
ZNF501	3.29	0.000189	0.0380
TCF21	3.29	1.91E-05	0.0158
CLDN12	3.28	9.19E-05	0.0301
RTF1	3.27	0.00024	0.0427
SULT1C2	3.27	9.91E-05	0.0301
PTP4A3	3.25	0.000304	0.0462
AC073592.3	3.25	5.06E-05	0.0247
AC048341.2	3.24	0.000215	0.0413
ZNF723	3.21	0.000102	0.0301
TACC2	3.20	0.000165	0.0357
LINC01634	3.19	0.000165	0.0357
AC106782.2	3.19	0.000247	0.0432
AC140479.2	3.19	5.6E-05	0.0247
CACNA2D3-AS1	3.19	0.000127	0.0328
ATG10	3.18	5.97E-06	0.0093
HEATR4	3.18	0.000194	0.0387
AL359851.1	3.17	7.59E-05	0.0287
AC023830.3	3.17	1.28E-05	0.0137
RDH8	3.16	0.000136	0.0331
MXRA5	3.15	0.000118	0.0325
TMPO-AS1	3.15	5.59E-05	0.0247
PITPNC1	3.15	5.81E-06	0.0093
ADNP	3.14	0.000167	0.0357
CFAP97	3.14	0.000107	0.0301
IGFBP2	3.13	5.38E-05	0.0247
POM121C	3.13	0.000317	0.0470
LRRC77P	3.11	0.000343	0.0482
AC015818.9	3.09	9.7E-06	0.0129
GDF3	3.09	5.37E-05	0.0247
PPP1R26P1	3.09	0.000119	0.0325
SRSF6	3.08	0.00013	0.0329
RAB5B	3.08	1.22E-05	0.0137
HIST1H4I	3.08	0.000308	0.0464
AC118758.3	3.07	0.000181	0.0379
JAKMIP3	3.05	0.000143	0.0337
DDX18	3.05	3.46E-05	0.0202
TNK2-AS1	3.04	0.000365	0.0487
ASCC1	3.03	0.000285	0.0446
ABI3	3.03	3.83E-05	0.0219
RHBDD2	3.02	0.000104	0.0301
RPUSD2	3.01	0.000208	0.0403
ZNF354A	2.99	0.000139	0.0331
CNGB1	2.99	9.13E-05	0.0301
PIK3AP1	2.97	9.55E-05	0.0301
PI4KAP1	2.97	3.75E-06	0.0080
TIAM1	2.96	0.000124	0.0327
AC145676.1	2.96	9.66E-05	0.0301
XPO4	2.95	0.000278	0.0438
TNFRSF10D	2.94	0.000275	0.0438
EIF5AL1	2.93	0.000359	0.0486
LINC01756	2.91	0.000106	0.0301
CTSB	2.90	0.000106	0.0301
LINC02363	2.88	8.52E-05	0.0301
VPS8	2.88	0.000341	0.0482
AL731577.1	2.88	0.000207	0.0403
TACR1	2.86	0.000255	0.0433
NDUFV3	2.83	1.58E-05	0.0153
PPM1G	2.82	1.27E-05	0.0137
PDF	2.82	0.000124	0.0327
CCNJ	2.82	5.39E-05	0.0247
NIPA1	2.82	0.000235	0.0425
TOMM22	2.82	0.000152	0.0347
COPE	2.80	0.000233	0.0425
ATF4	2.79	0.000133	0.0330
OR2J1	2.79	3.7E-06	0.0080
ARL14EP	2.76	3.73E-06	0.0080
AC009108.4	2.76	6.39E-06	0.0093
CBWD2	2.74	6.04E-05	0.0250
AC068189.1	2.74	2.12E-05	0.0158
IKBIP	2.72	0.000259	0.0436
GAPDHP75	2.71	4.03E-05	0.0219
PWAR5	2.70	6.07E-05	0.0250
PEG10	2.69	0.000326	0.0476
FABP5	2.69	0.000341	0.0482
CDC42BPG	2.68	9.96E-05	0.0301
NEDD4L	2.67	9.91E-05	0.0301
CADM3	2.67	0.000129	0.0328
SLC12A5	2.65	0.000346	0.0482
RHOJ	2.64	0.000308	0.0464
NUTM2A	2.64	0.000324	0.0475
ORAI1	2.64	0.000303	0.0462
AL022100.1	2.63	0.000115	0.0323
INSL3	2.63	0.000346	0.0482
SLC25A1	2.62	0.000375	0.0491
FABP5P3	2.62	0.000222	0.0422
EIF2AK3	2.62	0.000105	0.0301
FBXL13	2.58	1.11E-06	0.0072
AC006484.1	2.58	0.000134	0.0330
ELOA3	2.57	3.91E-05	0.0219
RIMBP2	2.56	2.28E-05	0.0158
GLIS2	2.56	0.000359	0.0486
SULT4A1	2.56	0.000346	0.0482
AL671511.1	2.55	0.000291	0.0454
GABRB3	2.51	0.000184	0.0379
KLC3	2.48	0.000387	0.0500
AP002370.1	2.45	0.00027	0.0438
CNTNAP5	2.45	1.01E-05	0.0129
AC010978.1	2.43	0.000103	0.0301
CYP4F8	2.39	0.000265	0.0438
LINC00298	2.37	9.07E-05	0.0301
GCFC2	2.35	1.74E-05	0.0155
TRMT11	2.35	0.000106	0.0301
HSPB8	2.35	0.000144	0.0337
AL133355.1	2.34	5.57E-05	0.0247
LINC02137	2.33	0.000134	0.0330
MAP1S	2.32	0.000267	0.0438
AC022762.2	2.31	0.000139	0.0331
EFHB	2.28	0.000187	0.0379
HAS2	2.28	0.000196	0.0388
OR4N2	2.26	0.000166	0.0357
NEIL2	2.22	2.33E-06	0.0080
NUDT9	2.20	7.81E-05	0.0292
AL603840.1	2.20	0.000331	0.0481
RLBP1	2.19	0.000236	0.0425
TFIP11	2.17	0.000243	0.0427
AL355303.1	2.15	0.000385	0.0500
PVALB	2.06	7.43E-05	0.0285
FAM89A	2.04	0.00023	0.0424
SLC25A6P5	1.98	1.85E-05	0.0158
AL807752.1	1.98	5.24E-05	0.0247
AL359697.1	1.96	3.48E-06	0.0080
FAM20A	1.93	0.000138	0.0331
PPFIA2	1.88	0.000338	0.0482
C6	1.88	1.12E-05	0.0134
AP000343.1	1.78	0.000352	0.0483
AC131235.4	1.74	0.000239	0.0427
RSF1-IT1	1.49	0.000223	0.0422

Significantly downregulated genes

Gene symbol	Log2FC	p value	q value
NQO2	-1.77	0.000388	0.0500
SHC1	-1.77	5.33E-05	0.0247
DPY19L2P3	-2.02	0.000208	0.0403
NYAP2	-2.05	1.52E-05	0.0153
PPM1F	-2.07	0.000356	0.0486
FCER2	-2.08	0.000363	0.0487
WFIKKN1	-2.26	0.00016	0.0357
ZNF627	-2.27	0.000148	0.0342
ATP1A4	-2.29	0.000166	0.0357
C12orf65	-2.30	0.000386	0.0500
TOX2	-2.38	0.000366	0.0487
SEH1L	-2.44	0.000243	0.0427
AC133919.2	-2.46	5.46E-05	0.0247
AC011468.3	-2.48	0.000371	0.0491
AC083805.1	-2.48	0.000168	0.0357
HEATR3	-2.48	0.000252	0.0433
ZBTB48	-2.57	0.000271	0.0438
AC133785.1	-2.65	0.000185	0.0379
CENPV	-2.68	0.000186	0.0379
SLC9A3R2	-2.69	8.09E-05	0.0294
PTDSS2	-2.73	0.000225	0.0422
AC074117.1	-2.76	0.00027	0.0438
TNFRSF13C	-2.82	0.000273	0.0438
AC012513.3	-2.85	0.000203	0.0399
MTCO1P9	-2.92	0.000318	0.0470
TMC2	-2.92	9.65E-05	0.0301
GACAT3	-2.99	0.000102	0.0301
HSFX4	-3.01	2.12E-05	0.0158
MIR194-2HG	-3.07	7.91E-05	0.0292
AC087500.2	-3.08	0.000314	0.0469
LMNB2	-3.13	0.000257	0.0435
FAM136A	-3.17	5.33E-05	0.0247
AC092296.3	-3.19	3.57E-07	0.0072
MKRN2OS	-3.19	0.000163	0.0357
AC093627.5	-3.20	0.000347	0.0482
AC082651.1	-3.24	0.000263	0.0438
YY2	-3.24	2.03E-05	0.0158
AC104123.1	-3.46	2.51E-05	0.0161
LINC02403	-3.54	0.000304	0.0462
AP002784.1	-3.62	0.000152	0.0347
MAGEB3	-3.62	0.000127	0.0328
TOMM20P1	-3.70	5.16E-05	0.0247
AL158166.2	-3.79	9.79E-06	0.0129
AL356583.3	-3.83	0.000361	0.0487
TMEM167B	-3.89	1.62E-05	0.0153
AC091948.1	-3.92	2.32E-05	0.0158
SLC35D1	-4.10	0.00023	0.0424
VDAC1P5	-4.18	7.22E-05	0.0283
TUSC2	-4.39	0.000145	0.0337
BUB1P1	-4.41	5.71E-05	0.0247
FRAT1	-4.55	6.69E-05	0.0272
ADGRE1	-4.60	0.000293	0.0454
ZNF683	-4.64	0.000128	0.0328
JADE3	-4.78	0.000254	0.0433
AC027176.2	-4.81	7.45E-05	0.0285
PTENP1-AS	-5.17	8.21E-05	0.0294
PDCD1LG2	-7.20	5.77E-05	0.0247

Supplementary Table S5. List of significant ontology terms across different ontology databases from gene set enrichment analysis (GSEA)

Ontology DB	ID	Description	Set Size	Enrichment Score	Normal Enrichment Score	p value	FDR p adjust
GO	GO:0001525	angiogenesis	465	0.393134823	1.496018605	2.77E-06	0.022269116
	GO:0007389	pattern specification process	421	0.389278851	1.477678076	1.19E-05	0.033684235
	GO:0034702	ion channel complex	281	0.411722289	1.540100953	1.94E-05	0.033684235
	GO:0010975	regulation of neuron projection development	482	0.372872381	1.419529338	2.53E-05	0.033684235
	GO:0090596	sensory organ morphogenesis	249	0.416241802	1.551386667	2.68E-05	0.033684235
	GO:0016741	transferase activity, transferring one-carbon groups	206	0.434353778	1.606311366	2.83E-05	0.033684235
	GO:0008168	methyltransferase activity	197	0.436844599	1.609289131	3.18E-05	0.033684235
	GO:0009952	anterior/posterior pattern specification	204	0.430182524	1.589109094	3.35E-05	0.033684235
	GO:0003002	regionalization	330	0.398298857	1.500966353	4.66E-05	0.034663656
	GO:0046660	female sex differentiation	111	0.494816972	1.737359093	5.03E-05	0.034663656
	GO:0048814	regulation of dendrite morphogenesis	91	0.512226958	1.75124446	5.08E-05	0.034663656
	GO:1902495	transmembrane transporter complex	301	0.40263697	1.514100333	5.18E-05	0.034663656
	GO:0008585	female gonad development	93	0.503280622	1.724897644	6.29E-05	0.038900005
	GO:0046545	development of primary female sexual characteristics	98	0.493830467	1.70758214	8.90E-05	0.042197272
	GO:0015693	magnesium ion transport	17	0.754962807	1.924433624	9.42E-05	0.042197272
	GO:0061001	regulation of dendritic spine morphogenesis	46	0.599365935	1.86343298	9.50E-05	0.042197272
	GO:0001654	eye development	353	0.385708953	1.455272325	0.00010962	0.042197272
	GO:0016782	transferase activity, transferring sulfur-containing groups	63	0.549884063	1.809155608	0.00011166	0.042197272
	GO:0150063	visual system development	357	0.382169876	1.441672211	0.00011201	0.042197272
	GO:0016180	snRNA processing	34	-0.554513098	-2.122626272	0.00011322	0.042197272
	GO:0019933	cAMP-mediated signaling	175	0.432152244	1.578588395	0.00011443	0.042197272
	GO:1990351	transporter complex	308	0.39818474	1.498190214	0.00011554	0.042197272
	GO:0034472	snRNA 3'-end processing	29	-0.575846119	-2.094308634	0.00014275	0.049871013
KEGG	hsa04015	Rap1 signaling pathway	194	0.433561546	1.575940228	0.00011675	0.020506651
	hsa04623	Cytosolic DNA-sensing pathway	51	-0.469505669	-1.919639974	0.00012279	0.020506651
REACTOME	R-HSA-76061	RNA Polymerase III Transcription Initiation From Type 1 Promoter	25	-0.700324679	-2.468287874	3.05E-06	0.002181753
	R-HSA-76066	RNA Polymerase III Transcription Initiation From Type 2 Promoter	25	-0.700324679	-2.468287874	3.05E-06	0.002181753
	R-HSA-2219530	Constitutive Signaling by Aberrant PI3K in Cancer	69	0.544817443	1.79980008	4.66E-05	0.018887428
	R-HSA-5223345	Miscellaneous transport and binding events	22	0.70947703	1.942006209	5.28E-05	0.018887428

Supplementary Table S6. Complete list of shared differentially expressed genes (DEGs) shared across different datasets.

Gene Symbol	Description	Isolated MCs	GDS4602	GSE78097	GSE80047
ABI3	ABI Family Member 3	3.03	0.18	0.00	0.00
ADGRE1	Adhesion G Protein-Coupled Receptor E1	-4.60	0.00	0.00	0.26
ADNP	Activity Dependent Neuroprotector Homeobox	3.14	0.00	0.00	0.09
AP5M1	Adaptor Related Protein Complex 5 Subunit Mu 1	3.56	0.00	0.00	0.09
ARL14EP	ADP Ribosylation Factor Like GTPase 14 Effector Protein	2.76	-0.34	0.00	0.00
ASCC1	Activating Signal Cointegrator 1 Complex Subunit 1	3.03	0.00	0.00	0.14
ATF4	Activating Transcription Factor 4	2.79	0.27	0.40	0.00
ATG10	Autophagy Related 10	3.18	0.00	0.00	-0.06
ATP1A4	ATPase Na+/K+ Transporting Subunit Alpha 4	-2.29	0.00	0.00	0.06
BPTF	Bromodomain PHD Finger Transcription Factor	4.02	0.00	0.00	-0.04
C12orf65	Mitochondrial Translation Release Factor In Rescue	-2.30	0.00	0.00	-0.15
C6	Complement C6	1.88	-0.27	0.00	0.00
CADM3	Cell Adhesion Molecule 3	2.67	0.00	0.00	-0.23
CASC17	Cancer Susceptibility 17	3.40	0.00	0.00	0.06
CBWD2	COBW Domain Containing 2	2.74	0.00	0.00	0.21
CCDC113	Coiled-Coil Domain Containing 113	3.71	-0.60	0.00	-0.13
CCNJ	Cyclin J	2.82	0.00	0.00	0.14
CDC42BPG	CDC42 Binding Protein Kinase Gamma	2.68	0.00	0.00	-0.44
CENPV	Centromere Protein V	-2.68	0.00	0.00	-0.16
CFAP97	Cilia And Flagella Associated Protein 97	3.14	0.00	0.00	0.07
CLDN12	Claudin 12	3.28	0.25	0.58	0.15
CLK4	CDC Like Kinase 4	3.53	0.00	0.00	-0.09
CNNM2	Cyclin And CBS Domain Divalent Metal Cation Transport Mediator 2	3.92	0.00	0.00	-0.19
CNTNAP5	Contactin Associated Protein Family Member 5	2.45	0.00	0.00	0.04
COPE	COPI Coat Complex Subunit Epsilon	2.80	0.00	0.00	0.06
CTSB	Cathepsin B	2.90	0.00	0.00	0.08
CYP4F8	Cytochrome P450 Family 4 Subfamily F Member 8	2.39	-0.82	0.00	0.00
DCAF4L1	DDB1 And CUL4 Associated Factor 4 Like 1	4.32	0.05	0.00	0.12
DDI2	DNA Damage Inducible 1 Homolog 2	5.18	0.00	0.00	-0.11
ELAC2	ElaC Ribonuclease Z 2	3.45	0.00	0.00	0.10
FABP5	Fatty Acid Binding Protein 5	2.69	1.46	1.03	0.19
FAM136A	Family With Sequence Similarity 136 Member A	-3.17	0.00	0.00	-0.02
FAM20A	FAM20A Golgi Associated Secretory Pathway Pseudokinase	1.93	0.00	0.00	0.09
FAM46B	Terminal Nucleotidyltransferase 5B	3.56	0.28	0.00	-0.14
FAM83E	Family With Sequence Similarity 83 Member E	3.47	0.07	0.00	-0.11
FBXL13	F-Box And Leucine Rich Repeat Protein 13	2.58	0.00	0.00	-0.16
FRAT1	FRAT Regulator Of WNT Signaling Pathway 1	-4.55	0.00	0.00	0.12
GDAP2	Ganglioside Induced Differentiation Associated Protein 2	3.31	0.00	0.00	0.13
GDF3	Growth Differentiation Factor 3	3.09	0.07	0.00	0.00
GLIS2	GLIS Family Zinc Finger 2	2.56	-0.63	-1.04	-0.12
GTPBP10	GTP Binding Protein 10	3.48	0.08	0.00	0.09
HAS2	Hyaluronan Synthase 2	2.28	0.00	0.00	0.31
HDAC9	Histone Deacetylase 9	3.83	0.00	0.00	-0.10
HEATR3	HEAT Repeat Containing 3	-2.48	0.00	0.00	0.36
HEATR4	HEAT Repeat Containing 4	3.18	0.00	0.00	-0.13
HIST1H4I	H4 Clustered Histone 9	3.08	0.16	0.00	0.00
HSPB8	Heat Shock Protein Family B (Small) Member 8	2.35	0.00	0.00	0.20
IFI44L	Interferon Induced Protein 44 Like	3.93	1.82	2.67	0.81
IGFBP2	Insulin Like Growth Factor Binding Protein 2	3.13	0.00	0.00	-0.25
ILDR2	Immunoglobulin Like Domain Containing Receptor 2	4.07	0.00	0.00	0.11
JADE3	Jade Family PHD Finger 3	-4.78	0.00	0.00	0.05
JAKMIP3	Janus Kinase And Microtubule Interacting Protein 3	3.05	0.00	0.00	-0.16
KLC3	Kinesin Light Chain 3	2.48	0.00	0.00	-0.24
LINC00298	Long Intergenic Non-Protein Coding RNA 298	2.37	0.00	0.00	0.09
LMNB2	Lamin B2	-3.13	0.93	2.75	0.23
LMOD2	Leiomodin 2	4.45	0.15	0.00	0.00
LRP2	LDL Receptor Related Protein 2	3.35	0.00	0.00	0.08
LTA	Lymphotoxin Alpha	4.16	0.12	0.00	0.00
MAGEB3	MAGE Family Member B3	-3.62	0.06	0.00	0.05
MALAT1	Metastasis Associated Lung Adenocarcinoma Transcript 1	3.39	0.00	0.00	-0.10
MXRA5	Matrix Remodeling Associated 5	3.15	-0.41	-0.54	-0.08

Log2FC: > 1; 0; < -1

q ≥ 0.05

**NAT14**	N-Acetyltransferase 14 (Putative)	3.99	0.15	-0.42	-0.16
**NDUFV3**	NADH:Ubiquinone Oxidoreductase Subunit V3	2.83	0.00	0.00	-0.09
**NEDD4L**	NEDD4 Like E3 Ubiquitin Protein Ligase	2.67	0.00	0.00	-0.07
**NEIL2**	Nei Like DNA Glycosylase 2	2.22	0.00	0.00	-0.02
**NIPA1**	NIPA Magnesium Transporter 1	2.82	0.00	0.00	0.13
**NQO2**	N-Ribosyldihydronicotinamide:Quinone Reductase 2	-1.77	0.00	0.00	-0.14
**NSUN5P1**	NSUN5 Pseudogene 1	3.64	0.00	0.00	-0.11
**NUDT9**	Nudix Hydrolase 9	2.20	0.00	0.78	0.09
**NYAP2**	Neuronal Tyrosine-Phosphorylated Phosphoinositide-3-Kinase Adaptor 2	-2.05	-0.05	0.00	0.00
**ORAI1**	ORAI Calcium Release-Activated Calcium Modulator 1	2.64	-0.38	-1.34	-0.15
**PACRG-AS3**	PACRG Antisense RNA 3	3.48	0.08	0.00	-0.07
**PDCD1LG2**	Programmed Cell Death 1 Ligand 2	-7.20	0.00	0.00	0.09
**PEG10**	Paternally Expressed 10	2.69	0.00	0.00	-0.15
**PHYKPL**	5-Phosphohydroxy-L-Lysine Phospho-Lyase	3.42	0.00	0.00	-0.09
**PIGN**	Phosphatidylinositol Glycan Anchor Biosynthesis Class N	3.83	0.00	0.00	0.17
**PIK3AP1**	Phosphoinositide-3-Kinase Adaptor Protein 1	2.97	0.00	0.00	0.16
**PITPNC1**	Phosphatidylinositol Transfer Protein Cytoplasmic 1	3.15	0.00	0.00	0.13
**POM121C**	POM121 Transmembrane Nucleoporin C	3.13	0.15	-0.40	0.00
**PPFIA2**	PTPRF Interacting Protein Alpha 2	1.88	0.00	0.00	-0.09
**PPM1F**	Protein Phosphatase, Mg2+/Mn2+ Dependent 1F	-2.07	0.00	0.00	0.13
**PPM1G**	Protein Phosphatase, Mg2+/Mn2+ Dependent 1G	2.82	0.29	0.00	-0.07
**PRDM2**	PR/SET Domain 2	4.42	0.00	0.00	-0.14
**PRDM4**	PR/SET Domain 4	3.84	0.00	0.00	0.05
**PRKCQ-AS1**	PRKCQ Antisense RNA 1	3.34	0.00	0.00	0.49
**PTDSS2**	Phosphatidylserine Synthase 2	-2.73	0.21	0.85	0.00
**PTP4A3**	Protein Tyrosine Phosphatase 4A3	3.25	0.00	0.00	-0.03
**PWAR5**	Prader Willi/Angelman Region RNA 5	2.70	-0.44	-1.46	-0.46
**RAB11FIP5**	RAB11 Family Interacting Protein 5	3.56	-0.25	-0.76	-0.22
**RAB5B**	RAB5B, Member RAS Oncogene Family	3.08	-0.39	-0.58	0.09
**RAD51D**	RAD51 Paralog D	4.21	0.00	0.00	0.12
**RHOJ**	Ras Homolog Family Member J	2.64	0.00	0.00	-0.18
**RIMBP2**	RIMS Binding Protein 2	2.56	0.00	0.00	0.06
**RLBP1**	Retinaldehyde Binding Protein 1	2.19	0.23	0.00	0.00
**RPAP2**	RNA Polymerase II Associated Protein 2	3.43	0.00	0.00	0.08
**RPS6KL1**	Ribosomal Protein S6 Kinase Like 1	3.79	0.17	0.00	0.00
**RPUSD2**	RNA Pseudouridine Synthase Domain Containing 2	3.01	0.15	-0.61	-0.09
**SEH1L**	SEH1 Like Nucleoporin	-2.44	0.00	0.00	0.11
**SHC1**	SHC Adaptor Protein 1	-1.77	0.00	0.00	0.18
**SLC12A5**	Solute Carrier Family 12 Member 5	2.65	0.00	0.00	0.10
**SLC25A1**	Solute Carrier Family 25 Member 1	2.62	-0.13	0.00	0.00
**SLC35B2**	Solute Carrier Family 35 Member B2	3.32	0.00	0.00	-0.21
**SLC35D1**	Solute Carrier Family 35 Member D1	-4.10	0.00	0.00	0.09
**SRSF6**	Serine And Arginine Rich Splicing Factor 6	3.08	0.00	0.00	0.13
**SUSD2**	Sushi Domain Containing 2	3.35	0.00	0.00	-0.14
**TACC2**	Transforming Acidic Coiled-Coil Containing Protein 2	3.20	0.00	0.00	-0.08
**TACR1**	Tachykinin Receptor 1	2.86	0.00	0.00	-0.12
**TIAM1**	TIAM Rac1 Associated GEF 1	2.96	0.00	0.00	0.13
**TMC2**	Transmembrane Channel Like 2	-2.92	0.13	0.00	0.07
**TMEM167B**	Transmembrane Protein 167B	-3.89	-0.60	-0.37	-0.21
**TMPO-AS1**	TMPO Antisense RNA 1	3.15	0.22	0.00	0.19
**TNFRSF10D**	TNF Receptor Superfamily Member 10d	2.94	0.00	0.00	0.07
**TNFRSF13C**	TNF Receptor Superfamily Member 13C	-2.82	0.10	0.00	0.06
**TNK2-AS1**	TNK2 Antisense RNA 1	3.04	0.00	0.00	0.06
**TOMM22**	Translocase Of Outer Mitochondrial Membrane 22	2.82	0.00	0.00	-0.05
**TOX2**	TOX High Mobility Group Box Family Member 2	-2.38	-0.43	0.00	-0.08
**TRMT11**	TRNA Methyltransferase 11 Homolog	2.35	-0.11	0.56	0.00
**TUSC2**	Tumor Suppressor 2, Mitochondrial Calcium Regulator	-4.39	0.00	0.00	-0.07
**UBXN4**	UBX Domain Protein 4	3.36	0.00	0.00	-0.06
**UQCRB**	Ubiquinol-Cytochrome C Reductase Binding Protein	3.73	0.00	0.00	-0.04
**XPO4**	Exportin 4	2.95	0.00	0.00	0.13
**ZBTB48**	Zinc Finger And BTB Domain Containing 48	-2.57	-0.15	0.00	-0.07
**ZNF354A**	Zinc Finger Protein 354A	2.99	0.00	0.69	0.30
**ZNF486**	Zinc Finger Protein 486	3.44	-0.14	-0.61	-0.15
**ZNF501**	Zinc Finger Protein 501	3.29	-0.30	-1.34	-0.11
**ZNF627**	Zinc Finger Protein 627	-2.27	0.00	0.00	0.13
**ZNF683**	Zinc Finger Protein 683	-4.64	0.24	0.00	0.00

Supplementary Table S7. Enriched canonical pathways, upstream regulators and causal networks identified in isolated MCs by IPA.

**Isolated MCs**			
Canonical pathway	z-score	Fisher exact test p value	B-H p adjusted
CLEAR Signaling Pathway	1.633	**1.82E-05**	0.616
Autophagy	2.236 (biased)	**0.0207**	0.616
PI3K Signaling in B Lymphocytes	1 (biased)	**0.0207**	0.616
Upstream regulators	z-score	p value overlap	B-H p adjusted
IL1B	1.792	**0.0218**	0.236
NFkB complex	0.677 (bias-corrected)	**0.0117**	0.259
Causal network analysis	z-score	Network bias-corrected p value	B-H p adjusted
IFN	2.023	**2.00E-04**	0.0958
IL-12 cytokine families	3.024 (biased)	**7.90E-03**	0.166

Supplementary Table S8. Enriched canonical pathways, upstream regulators and causal networks identified in published microarray datasets by IPA.

GDS4602			
Canonical pathway	z-score	p value overlap	B-H p adjusted
CLEAR Signaling Pathway	0.258	**4.78E-02**	0.3
Autophagy	0.7	**8.09E-03**	0.116
Upstream regulators	z-score	p value overlap	B-H p adjusted
IL1B	6.234	**1.22E-05**	**1.69E-03**
NFkB complex	5.239	**6.10E-05**	**4.66E-03**

GSE80047			
Canonical pathway	z-score	p value overlap	B-H p adjusted
CLEAR Signaling Pathway	-1.414	**2.66E-04**	**1.56E-03**
Autophagy	1.26	**2.81E-03**	**0.0102**
Upstream regulators	z-score	p value overlap	B-H p adjusted
IL1B	7.961	**3.94E-22**	**2.45E-19**
NFkB complex	7.164	**4.67E-11**	**3.84E-09**

GSE78097			
Canonical pathway	z-score	p value overlap	B-H p adjusted
CLEAR Signaling Pathway	0.649	**2.40E-02**	0.236
Autophagy	1.061	**8.42E-03**	0.25
Upstream regulators	z-score	p value overlap	B-H p adjusted
IL1B	4.893	**7.33E-05**	**5.68E-03**
NFkB complex	2.995	**0.0133**	0.18
Causal network analysis	z-score	p value overlap	B-H p adjusted
IL-12 cytokine families	4.538	**1.24E-08**	**2.54E-07**

Supplementary Table S9. List of itch-associated genes from published literature.

Category	Gene	Species used in study	Reference
Amine receptor	*HRH1*	Human, Mouse	(Amatya et al., 2010; Darsow et al., 1997; Hägermark et al., 1978; Hagermark & Strandberg, 1977; Inagaki et al., 1999; Oetjen et al., 2017; Rossbach et al., 2011)
Amine receptor	*HRH2*	Human, Mouse	(Amatya et al., 2010; Darsow et al., 1997; Hägermark et al., 1978; Hagermark & Strandberg, 1977; Inagaki et al., 1999; Nattkemper et al., 2018; Oetjen et al., 2017)
Amine receptor	*HRH3*	Human, Mouse	(Amatya et al., 2010; Darsow et al., 1997; Hägermark et al., 1978; Hagermark & Strandberg, 1977; Nattkemper et al., 2018; Oetjen et al., 2017; Rossbach et al., 2011)
Amine receptor	*HRH4*	Human, Mouse	(Amatya et al., 2010; Darsow et al., 1997; Hägermark et al., 1978; Hagermark & Strandberg, 1977; Nattkemper et al., 2018; Oetjen et al., 2017; Rossbach et al., 2009, 2011)
Amine-processing enzyme	*HDC*	Human	(Amatya et al., 2010; Darsow et al., 1997; Hägermark et al., 1978; Hagermark & Strandberg, 1977; Oetjen et al., 2017)
Calcium-binding protein	*S100A14*	Human	(Nattkemper et al., 2018)
Calcium-binding protein	*S100A2*	Human	(Nattkemper et al., 2018)
Calcium-binding protein	*S100A7*	Human	(Nattkemper et al., 2018)
Calcium-binding protein	*S100A9*	Human	(Nattkemper et al., 2018)
Calcium-binding protein	*S100G*	Human	(Nattkemper et al., 2018)
Calcium-binding protein	*S100P*	Human	(Nattkemper et al., 2018)
Cannabinoid receptor	*CNR1*	Human	(Nattkemper et al., 2018)
Cannabinoid receptor	*CNR2*	Human	(Nattkemper et al., 2018)
Cell adhesion molecule	*AOC3*	Human	(Madej et al., 2007)
Cell adhesion molecule	*CADM1*	Mouse	(Furuno et al., 2012; Steinhoff et al., 2006)
Cell adhesion molecule	*NECTIN3*	Mouse	(Furuno et al., 2012; Steinhoff et al., 2006)
Cell adhesion molecule	*SELE*	Human	(Nakamura et al., 2003)
Chemokine	*CCL1*	Human	(Nattkemper et al., 2018)
Chemokine	*CCL14*	Human	(Nattkemper et al., 2018)
Chemokine	*CCL17*	Human	(Nattkemper et al., 2018)
Chemokine	*CCL18*	Human	(Nattkemper et al., 2018)
Chemokine	*CCL2*	Human	(Nattkemper et al., 2018)
Chemokine	*CCL20*	Human	(Nattkemper et al., 2018)
Chemokine	*CCL26*	Human	(Nattkemper et al., 2018)
Chemokine	*CCL27*	Human	(Nattkemper et al., 2018)
Chemokine	*CCL3*	Human	(Nattkemper et al., 2018)
Chemokine	*CCL4*	Human	(Nattkemper et al., 2018)
Chemokine	*CCL7*	Human	(Nattkemper et al., 2018)
Chemokine	*CCL8*	Human	(Nattkemper et al., 2018)
Chemokine	*CXCL1*	Human	(Nattkemper et al., 2018)
Chemokine	*CXCL10*	Human	(Nattkemper et al., 2018)
Chemokine	*CXCL11*	Human	(Nattkemper et al., 2018)
Chemokine	*CXCL3*	Human	(Nattkemper et al., 2018)

Chemokine	*CXCL8*	Human	(Nattkemper et al., 2018)
Chemokine receptor	*CXCR1*	Human	(Nattkemper et al., 2018)
Chemokine receptor	*CXCR3*	Human	(Nattkemper et al., 2018)
Cytokine	*IFNG*	Human	(Nattkemper et al., 2018)
Cytokine	*IL10*	Human	(Nattkemper et al., 2018)
Cytokine	*IL13*	Human, Mouse	(Oetjen et al., 2017; Oh et al., 2013; Zheng et al., 2009)
Cytokine	*IL17A*	Human	(Nattkemper et al., 2018)
Cytokine	*IL17F*	Human	(Nattkemper et al., 2018)
Cytokine	*IL19*	Human	(Nattkemper et al., 2018)
Cytokine	*IL2*	Human	(Darsow et al., 1997; Nakamura et al., 2003)
Cytokine	*IL20*	Human	(Nattkemper et al., 2018)
Cytokine	*IL22*	Human	(Nattkemper et al., 2018)
Cytokine	*IL23A*	Human	(Nattkemper et al., 2018)
Cytokine	*IL26*	Human	(Nattkemper et al., 2018)
Cytokine	*IL31*	Human, Mouse	(Andoh et al., 2017; Cevikbas et al., 2014; Dillon et al., 2004; Nattkemper et al., 2018; Oetjen et al., 2017; Sonkoly et al., 2006)
Cytokine	*IL36A*	Human	(Nattkemper et al., 2018)
Cytokine	*IL36G*	Human	(Nattkemper et al., 2018)
Cytokine	*IL4*	Human, Mouse	(Chan et al., 2001; Nattkemper et al., 2018; Oetjen et al., 2017)
Cytokine	*IL6*	Human	(Konda et al., 2015; Nattkemper et al., 2018)
Cytokine	*IL7*	Human	(Nattkemper et al., 2018)
Cytokine	*IL9*	Human	(Nattkemper et al., 2018)
Cytokine	*TNF*	Human, Mouse	(Kakurai et al., 2006; Miao et al., 2018; Nattkemper et al., 2018)
Cytokine	*TSLP*	Human, Mouse	(Wilson et al., 2013)
Cytokine receptor	*CRLF2*	Human, Mouse	(Wilson et al., 2013)
Cytokine receptor	*IL13RA1*	Human, Mouse	(Oetjen et al., 2017; Oh et al., 2013; Zheng et al., 2009)
Cytokine receptor	*IL13RA2*	Human, Mouse	(Oetjen et al., 2017; Oh et al., 2013; Zheng et al., 2009)
Cytokine receptor	*IL2RA*	Human	(Darsow et al., 1997; Nakamura et al., 2003)
Cytokine receptor	*IL2RB*	Human	(Darsow et al., 1997; Nakamura et al., 2003)
Cytokine receptor	*IL31RA*	Human, Mouse	(Andoh et al., 2017; Cevikbas et al., 2014; Dillon et al., 2004; Oetjen et al., 2017; Sonkoly et al., 2006)
Cytokine receptor	*IL4R*	Human, Mouse	(Chan et al., 2001; Oetjen et al., 2017)
Cytokine receptor	*IL6R*	Human	(Konda et al., 2015)
Cytokine receptor	*TNFRSF1A*	Mouse	(Kakurai et al., 2006; Miao et al., 2018)
Cytokine receptor	*TNFRSF1B*	Mouse	(Kakurai et al., 2006; Miao et al., 2018)
Endopeptidase	*ECEL1*	Human	(Nattkemper et al., 2018)
Hormone	*SST*	Mouse	(Stantcheva et al., 2016)
Ion channel	*CHRNA9*	Human	(Nattkemper et al., 2018)

Ion channel	*CHRNB3*	Human	(Nattkemper et al., 2018)
Ion channel	*P2RX3*	Mouse, Rat	(Schüttenhelm et al., 2015; Shiratori-Hayashi et al., 2019)
Ion channel	*PIEZO2*	Mouse	(Feng et al., 2018)
Ion channel	*TRPA1*	Human, Mouse	(Cevikbas et al., 2014; Nattkemper et al., 2018; Oh et al., 2013; Wilson et al., 2011, 2013)
Ion channel	*TRPM8*	Human	(Nattkemper et al., 2018)
Ion channel	*TRPV1*	Human, Mouse	(Cevikbas et al., 2014; Costa et al., 2008; Imamachi et al., 2009; Min et al., 2014; Nattkemper et al., 2018; Rogoz et al., 2014)
Ion channel	*TRPV2*	Human, Rat	(Nattkemper et al., 2018; Stokes et al., 2004)
Ion channel	*TRPV3*	Human, Mouse	(Cui et al., 2018; Nattkemper et al., 2018; Yoshioka et al., 2009; Zhao et al., 2020)
Ion channel	*TRPV4*	Mouse	(Luo et al., 2018)
Kinin autacoid	*KNG1*	Human	(Hosogi et al., 2006)
Kinin autacoid receptor	*BDKRB1*	Human	(Hosogi et al., 2006)
Kinin autacoid receptor	*BDKRB2*	Human	(Hosogi et al., 2006)
Lipid precursor processor	*LTA4H*	Mouse	(Andoh et al., 2011a, 2017; Andoh & Kuraishi, 1998)
Lipid precursor processor	*PTGDS*	Mouse	(Sugimoto et al., 2007)
Lipid precursor processor	*PTGES*	Human	(Fjellner & Hagermark, 1979; Hägermark et al., 1977; Hagermark & Strandberg, 1977; Neisius et al., 2002)
Lipid precursor processor	*PTGES2*	Human	(Fjellner & Hagermark, 1979; Hägermark et al., 1977; Hagermark & Strandberg, 1977; Neisius et al., 2002)
Lipid precursor processor	*PTGES3*	Human	(Fjellner & Hagermark, 1979; Hägermark et al., 1977; Hagermark & Strandberg, 1977; Neisius et al., 2002)
Lipid precursor processor	*PTGS1*	Human	(Hägermark et al., 1977)
Lipid precursor processor	*PTGS2*	Human	(Hägermark et al., 1977)
Lipid receptor	*LTB4R*	Mouse	(Andoh et al., 2011a, 2017; Andoh & Kuraishi, 1998)
Lipid receptor	*LTB4R2*	Mouse	(Andoh et al., 2011a, 2017; Andoh & Kuraishi, 1998)
Lipid receptor	*PTGER1*	Human	(Fjellner & Hagermark, 1979; Hägermark et al., 1977; Hagermark & Strandberg, 1977; Neisius et al., 2002)
Lipid receptor	*PTGER2*	Human	(Fjellner & Hagermark, 1979; Hägermark et al., 1977; Hagermark & Strandberg, 1977; Neisius et al., 2002)
Lipid receptor	*PTGER3*	Human	(Fjellner & Hagermark, 1979; Hägermark et al., 1977; Hagermark & Strandberg, 1977; Neisius et al., 2002)
Lipid receptor	*PTGER4*	Human	(Fjellner & Hagermark, 1979; Hägermark et al., 1977; Hagermark & Strandberg, 1977; Neisius et al., 2002)
Lipid receptor	*TBXA2R*	Mouse	(Andoh et al., 2007)
Mas-related G protein-coupled receptor	*MRGPRD*	Human, Mouse	(Liu et al., 2012; Nattkemper et al., 2018; Qu et al., 2014)
Mas-related G protein-coupled receptor	*MRGPRX1*	Human, Mouse	(Liu et al., 2009, 2011; Reddy et al., 2015; Sikand et al., 2011; Wilson et al., 2011)
Mas-related G-protein coupled receptor	*MRGPRX2*	Human, Mouse	(Azimi et al., 2016; Liu et al., 2011; Meixiong et al., 2019a; Nattkemper et al., 2018)
Mas-related G protein-coupled receptor	*MRGPRX3*	Human	(Nattkemper et al., 2018)
Mas-related G protein-coupled receptor	*MRGPRX4*	Human, Mouse	(Meixiong et al., 2019b; Yu et al., 2019)
Muscarinic receptor	*CHRM1*	Human	(Nattkemper et al., 2018)
Muscarinic receptor	*CHRM3*	Human	(Nattkemper et al., 2018)
Nerve fibre marker	*UCHL1*	Human, Mouse, Rat	(Kakurai et al., 2006; Nakamura et al., 2003; Schüttenhelm et al., 2015)
Neuropeptide	*NGF*	Human, Mouse	(Kou et al., 2012; Nakamura et al., 2003; Takano et al., 2005)
Neuropeptide	*NMB*	Mouse	(Wan et al., 2017)

Neuropeptide	*NMU*	Mouse	(Mizukawa et al., 2016)
Neuropeptide	*NPY*	Human, Mouse	(Bourane et al., 2015; Gao et al., 2018; Reich et al., 2007)
Neuropeptide	*VIP*	Human	(Fjellner & Hagermark, 1981)
Neuropeptide	*CALCA*	Mouse, Rat	(Kakurai et al., 2006; Rogoz et al., 2014; Schüttenhelm et al., 2015)
Neuropeptide precursor	*GRP*	Mouse	(Alemi et al., 2013; Andoh et al., 2011b; Wan et al., 2017)
Neuropeptide precursor	*NPPA*	Human, Mouse	(Shimizu et al., 2014; Solinski et al., 2019)
Neuropeptide precursor	*NPPB*	Human, Mouse	(Shimizu et al., 2014; Solinski et al., 2019)
Neuropeptide precursor	*NPPC*	Human, Mouse	(Shimizu et al., 2014; Solinski et al., 2019)
Neuropeptide precursor	*PNOC*	Mouse	(Andoh et al., 2004)
Neuropeptide precursor	*TAC1*	Human, Mouse, Rat	(Amatya et al., 2010; Andoh et al., 1998; Azimi et al., 2017; Hägermark et al., 1978; Nakamura et al., 2003; Nattkemper et al., 2018; Schüttenhelm et al., 2015)
Neuropeptide receptor	*CALCRL*	Mouse, Rat	(Kakurai et al., 2006; Rogoz et al., 2014; Schüttenhelm et al., 2015)
Neuropeptide receptor	*GPBAR1*	Mouse	(Alemi et al., 2013; Andoh et al., 2011b; Wan et al., 2017)
Neuropeptide receptor	*GRPR*	Mouse	(Andoh et al., 2011b; Wan et al., 2017)
Neuropeptide receptor	*NGFR*	Human, Mouse	(Kou et al., 2012; Nakamura et al., 2003; Takano et al., 2005)
Neuropeptide receptor	*NMBR*	Mouse	(Wan et al., 2017)
Neuropeptide receptor	*NPR1*	Human, Mouse	(Shimizu et al., 2014; Solinski et al., 2019)
Neuropeptide receptor	*NPR2*	Human, Mouse	(Shimizu et al., 2014; Solinski et al., 2019)
Neuropeptide receptor	*NPR3*	Human, Mouse	(Shimizu et al., 2014; Solinski et al., 2019)
Neuropeptide receptor	*NPY1R*	Human, Mouse	(Bourane et al., 2015; Gao et al., 2018; Reich et al., 2007)
Neuropeptide receptor	*NPY2R*	Human, Mouse	(Bourane et al., 2015; Gao et al., 2018; Reich et al., 2007)
Neuropeptide receptor	*NPY4R*	Human, Mouse	(Bourane et al., 2015; Gao et al., 2018; Reich et al., 2007)
Neuropeptide receptor	*NPY5R*	Human, Mouse	(Bourane et al., 2015; Gao et al., 2018; Reich et al., 2007)
Neuropeptide receptor	*RAMP1*	Mouse, Rat	(Kakurai et al., 2006; McLatchie et al., 1998; Rogoz et al., 2014; Schüttenhelm et al., 2015)
Neuropeptide receptor	*TACR1*	Human, Mouse, Rat	(Amatya et al., 2010; Andoh et al., 1998; Hägermark et al., 1978; Nakamura et al., 2003; Nattkemper et al., 2018; Schüttenhelm et al., 2015)
Neuropeptide receptor	*VIPR1*	Human	(Fjellner & Hagermark, 1981)
Neuropeptide receptor	*VIPR2*	Human	(Fjellner & Hagermark, 1981)
Neurotransmitter precursor processor	*CHAT*	Human	(Nattkemper et al., 2018)
Neurotransmitter precursor processor	*DDC*	Human, Mouse, Rat	(Balaskas et al., 1998; Fjellner & Hagermark, 1979; Hosogi et al., 2006; Nojima & Carstens, 2003; Thomsen et al., 2001; Weisshaar et al., 1999; Yamaguchi et al., 1999)
Neurotransmitter precursor processor	*TPH1*	Human, Mouse, Rat	(Balaskas et al., 1998; Fjellner & Hagermark, 1979; Hosogi et al., 2006; Nojima & Carstens, 2003; Thomsen et al., 2001; Weisshaar et al., 1999; Yamaguchi et al., 1999)
Neurotransmitter precursor processor	*TPH2*	Human, Mouse, Rat	(Balaskas et al., 1998; Fjellner & Hagermark, 1979; Hosogi et al., 2006; Nojima & Carstens, 2003; Thomsen et al., 2001; Weisshaar et al., 1999; Yamaguchi et al., 1999)
Neurotransmitter receptor	*GABRA1*	Human	(Nigam et al., 2010)
Neurotransmitter receptor	*GABRA2*	Human	(Nigam et al., 2010)
Neurotransmitter receptor	*GABRA3*	Human	(Nigam et al., 2010)
Neurotransmitter receptor	*GABRA4*	Human	(Nigam et al., 2010)
Neurotransmitter receptor	*GABRA5*	Human	(Nigam et al., 2010)
Neurotransmitter receptor	*GABRA6*	Human	(Nigam et al., 2010)

Neurotransmitter receptor	*HTR2A*	Human, Mouse, Rat	(Fjellner & Hagermark, 1979; Hosogi et al., 2006; Nojima & Carstens, 2003; Thomsen et al., 2001; Yamaguchi et al., 1999)
Neurotransmitter receptor	*HTR2B*	Human, Mouse, Rat	(Fjellner & Hagermark, 1979; Hosogi et al., 2006; Nojima & Carstens, 2003; Thomsen et al., 2001; Yamaguchi et al., 1999)
Neurotransmitter receptor	*HTR2C*	Human, Mouse, Rat	(Fjellner & Hagermark, 1979; Hosogi et al., 2006; Nojima & Carstens, 2003; Thomsen et al., 2001; Yamaguchi et al., 1999)
Neurotransmitter receptor	*HTR3A*	Human, Rat	(Balaskas et al., 1998; Fjellner & Hagermark, 1979; Hosogi et al., 2006; Thomsen et al., 2001; Weisshaar et al., 1999)
Neurotransmitter receptor	*HTR3B*	Human, Rat	(Balaskas et al., 1998; Fjellner & Hagermark, 1979; Hosogi et al., 2006; Nattkemper et al., 2018; Thomsen et al., 2001; Weisshaar et al., 1999)
Neurotransmitter receptor	*HTR3C*	Human, Rat	(Balaskas et al., 1998; Fjellner & Hagermark, 1979; Hosogi et al., 2006; Nattkemper et al., 2018; Thomsen et al., 2001; Weisshaar et al., 1999)
Neurotransmitter receptor	*HTR7*	Human	(Nattkemper et al., 2018)
Neurotrophic factor	*NRTN*	Mouse	(Heuckeroth et al., 1999; Sakai et al., 2017)
Neurotrophic factor	*NTF4*	Human	(Grewe et al., 2000)
Neurotrophic factor receptor	*GFRA1*	Mouse	(Heuckeroth et al., 1999; Sakai et al., 2017)
Neurotrophic factor receptor	*GFRA2*	Mouse	(Heuckeroth et al., 1999; Sakai et al., 2017)
Neurotrophic factor receptor	*GFRA3*	Mouse	(Heuckeroth et al., 1999; Sakai et al., 2017)
Neurotrophic factor receptor	*GFRA4*	Mouse	(Heuckeroth et al., 1999; Sakai et al., 2017)
Neurotrophic factor receptor	*NTRK1*	Human, Mouse	(Nakamura et al., 2003; Stantcheva et al., 2016)
Neurotrophic factor receptor	*NTRK2*	Human	(Grewe et al., 2000)
Monooxygenase	*TBXAS1*	Mouse	(Andoh et al., 2007)
Opioid precursor	*PDYN*	Human	(Taneda et al., 2011)
Opioid precursor	*PENK*	Human	(Sikand et al., 2011)
Opioid receptor	*OPRK1*	Human	(Nattkemper et al., 2018; Taneda et al., 2011)
Opioid receptor	*OPRL1*	Mouse	(Andoh et al., 2004)
Phospholipase	*PLA2G4B*	Human	(Nattkemper et al., 2018)
Phospholipase	*PLA2G4D*	Human	(Nattkemper et al., 2018)
Phospholipase	*PLA2G4E*	Human	(Nattkemper et al., 2018)
Phospholipase	*PLCB3*	Mouse	(Imamachi et al., 2009)
Phospholipase	*PLCG1*	Human	(Nattkemper et al., 2018)
Phospholipid receptor	*PTAFR*	Mouse	(Solinski et al., 2019)
Potassium channel	*KCNA1*	Mouse	(Lee et al., 2012)
Potassium channel	*KCNA2*	Mouse	(Lee et al., 2012)
Potassium channel	*KCNK2*	Mouse	(Lee et al., 2012)
Potassium channel	*KCNK4*	Mouse	(Lee et al., 2012)
Preproprotein	*POMC*	Human	(Nattkemper et al., 2018)
Protease	*CMA1*	Mouse	(Imada et al., 2002; Terakawa et al., 2008)
Protease	*CTSB*	Human	(Nattkemper et al., 2018)
Protease	*CTSF*	Human	(Nattkemper et al., 2018)
Protease	*CTSG*	Human	(Nattkemper et al., 2018)
Protease	*CTSH*	Human	(Nattkemper et al., 2018)

Protease	*CTSS*	Human	(Nattkemper et al., 2018; Reddy et al., 2015)
Protease	*CTSZ*	Human	(Nattkemper et al., 2018)
Protease	*ELANE*	Human	(Nattkemper et al., 2018)
Protease	*KLK13*	Human	(Nattkemper et al., 2018)
Protease	*KLK14*	Human, Rat	(Nattkemper et al., 2018; Oikonomopoulou et al., 2006; Stefansson et al., 2008; Steinhoff et al., 2003)
Protease	*KLK5*	Human, Rat	(Nattkemper et al., 2018; Oikonomopoulou et al., 2006; Stefansson et al., 2008; Steinhoff et al., 2003)
Protease	*KLK6*	Human, Rat	(Nattkemper et al., 2018; Oikonomopoulou et al., 2006; Steinhoff et al., 2003)
Protease	*KLK7*	Human, Mouse	(Ekholm & Egelrud, 1999; Guo et al., 2020; Hansson et al., 2002)
Protease	*KLK8*	Human	(Nattkemper et al., 2018)
Protease	*MMP9*	Human	(Takai et al., 2017)
Protease	*PRSS1*	Human, Mouse	(Costa et al., 2008; Thomsen et al., 2002)
Protease	*TPSAB1*	Human, Mouse	(Nattkemper et al., 2018; Steinhoff et al., 2003; Tsujii et al., 2009; Ui et al., 2006; Zhu et al., 2015)
Protein kinase	*MAPK1*	Mouse	(Chen et al., 2016; Zhang et al., 2014)
Protein kinase	*RET*	Mouse	(Stantcheva et al., 2016)
Protein kinase	*ROCK1*	Mouse	(Hashimoto et al., 2004)
Protein kinase	*ROCK2*	Mouse	(Hashimoto et al., 2004)
Proteinase activated receptor	*F2R*	Mouse	(Tsujii et al., 2008)
Proteinase activated receptor	*F2RL1*	Human, Mouse	(Costa et al., 2008; Nattkemper et al., 2018; Steinhoff et al., 2003; Tsujii et al., 2008, 2009)
Proteinase activated receptor	*F2RL2*	Human	(Nattkemper et al., 2018)
Proteinase activated receptor	*F2RL3*	Mouse	(Nattkemper et al., 2018; Patricio et al., 2015; Tsujii et al., 2008)
Semaphorin	*SEMA3A*	Human	(Kou et al., 2012)
Sodium channel	*SCN11A*	Human	(Nattkemper et al., 2018)
Sodium channel	*SCN3A*	Human	(Nattkemper et al., 2018)
Sodium channel	*SCN9A*	Human	(Nattkemper et al., 2018)
Toll-like receptor	*TLR4*	Mouse	(Min et al., 2014)
Toll-like receptor	*TLR7*	Mouse	(Liu et al., 2010)
Tyrosine kinase	*JAK1*	Human, Mouse	(Oetjen et al., 2017)
Vasoconstrictor	*EDN1*	Human	(Katugampola et al., 2000; Nattkemper et al., 2018; Wenzel et al., 1998)
Vasoconstrictor	*EDN2*	Human	(Katugampola et al., 2000; Wenzel et al., 1998)
Vasoconstrictor	*EDN3*	Human	(Katugampola et al., 2000; Wenzel et al., 1998)
Vasoconstrictor receptor	*EDNRA*	Human	(Nattkemper et al., 2018)

Alemi, F., Kwon, E., Poole, D. P., Lieu, T., Lyo, V., Cattaruzza, F., et al. (2013). The TGR5 receptor mediates bile acid-induced itch and analgesia. J. Clin. Invest. 123, 1513–1530. doi:10.1172/JCI64551.

Amatya, B., Nordlind, K., and Wahlgren, C. F. (2010). Responses to intradermal injections of substance P in psoriasis patients with pruritus. Skin Pharmacol. Physiol. 23, 133–138. doi:10.1159/000270385.

Andoh, T., Harada, A., and Kuraishi, Y. (2017). Involvement of leukotriene B4 released from keratinocytes in itch-associated response to intradermal interleukin-31 in mice. Acta Derm. Venereol. 97, 922–927. doi:10.2340/00015555-2697.

Andoh, T., Haza, S., Saito, A., and Kuraishi, Y. (2011a). Involvement of leukotriene B4 in spontaneous itch-related behaviour in NC mice with atopic dermatitis-like skin lesions. Exp. Dermatol. 20, 894–898. doi:10.1111/j.1600-0625.2011.01346.x.

Andoh, T., and Kuraishi, Y. (1998). Intradermal leukotriene B4, but not prostaglandin E2, induces itch-associated responses in mice. Eur. J. Pharmacol. 353, 93–96. doi:10.1016/s0014-2999(98)00440-3.

Andoh, T., Kuwazono, T., Lee, J.-B., and Kuraishi, Y. (2011b). Gastrin-releasing peptide induces itch-related responses through mast cell degranulation in mice. Peptides 32, 2098–2103. doi:10.1016/j.peptides.2011.09.003.

Andoh, T., Nagasawa, T., Satoh, M., and Kuraishi, Y. (1998). Substance P induction of itch-associated response mediated by cutaneous NK1 tachykinin receptors in mice. J. Pharmacol. Exp. Ther. 286, 1140–1145. Available at: http://www.ncbi.nlm.nih.gov/pubmed/9732370.

Andoh, T., Nishikawa, Y., Yamaguchi-Miyamoto, T., Nojima, H., Narumiya, S., and Kuraishi, Y. (2007). Thromboxane A2 induces itch-associated responses through TP receptors in the skin in mice. J. Invest. Dermatol. 127, 2042–2047. doi:10.1038/sj.jid.5700810.

Andoh, T., Yageta, Y., Takeshima, H., and Kuraishi, Y. (2004). Intradermal Nociceptin Elicits Itch-Associated Responses Through Leukotriene B4 in Mice. J. Invest. Dermatol. 123, 196–201. doi:10.1111/J.0022-202X.2004.22704.X.

Azimi, E., Reddy, V. B., Pereira, P. J. S., Talbot, S., Woolf, C. J., and Lerner, E. A. (2017). Substance P activates Mas-related G protein–coupled receptors to induce itch. J. Allergy Clin. Immunol. 140, 447-453.e3. doi:10.1016/j.jaci.2016.12.980.

Azimi, E., Reddy, V. B., Shade, K. T. C., Anthony, R. M., Talbot, S., Pereira, P. J. S., et al. (2016). Dual action of neurokinin-1 antagonists on Mas-related GPCRs. JCI Insight 1, 89362. doi:10.1172/JCI.INSIGHT.89362.

Balaskas, E. V, Bamihas, G. I., Karamouzis, M., Voyiatzis, G., and Tourkantonis, A. (1998). Histamine and serotonin in uremic pruritus: Effect of ondansetron in CAPD-pruritic patients. Nephron 78, 395–402. doi:10.1159/000044967.

Bourane, S., Duan, B., Koch, S. C., Dalet, A., Britz, O., Garcia-Campmany, L., et al. (2015). Gate control of mechanical itch by a subpopulation of spinal cord interneurons. Science (80-.). 350, 550–554. doi:10.1126/SCIENCE.AAC8653/SUPPL_FILE/BOURANE.SM.PDF.

Cevikbas, F., Wang, X., Akiyama, T., Kempkes, C., Savinko, T., Antal, A., et al. (2014). A sensory neuron-expressed IL-31 receptor mediates T helper cell-dependent itch: Involvement of TRPV1 and TRPA1. J. Allergy Clin. Immunol. 133. doi:10.1016/j.jaci.2013.10.048.

Chan, L. S., Robinson, N., and Xu, L. (2001). Expression of Interleukin-4 in the Epidermis of Transgenic Mice Results in a Pruritic Inflammatory Skin Disease: An Experimental Animal Model to Study Atopic Dermatitis. J. Invest. Dermatol. 117, 977–983. doi:10.1046/J.0022-202X.2001.01484.X.

Chen, Y., Jiang, S., Liu, Y., Xiong, J., Liang, J., and Ji, W. (2016). Role of ERK1/2 activation on itch sensation induced by bradykinin B1 activation in inflamed skin. Exp. Ther. Med. 12, 627–632. doi:10.3892/etm.2016.3426.

Costa, R., Marotta, D. M., Manjavachi, M. N., Fernandes, E. S., Lima-Garcia, J. F., Paszcuk, A. F., et al. (2008). Evidence for the role of neurogenic inflammation components in trypsin-elicited scratching behaviour in mice. Br. J. Pharmacol. 154, 1094–1103. doi:10.1038/BJP.2008.172.

Cui, T. T., Wang, G. X., Wei, N. N., and Wang, K. W. (2017). A pivotal role for the activation of TRPV3 channel in itch sensations induced by the natural skin sensitizer carvacrol. Acta Pharmacol. Sin. 2018 393 39, 331–335. doi:10.1038/aps.2017.152.

Darsow, U., Scharein, E., Bromm, B., and Ring, J. (1997). Skin testing of the pruritogenic activity of histamine and cytokines (interleukin-2 and tumour necrosis factor-alpha) at the dermal-epidermal junction. Br. J. Dermatol. 137, 415–417. Available at: http://www.ncbi.nlm.nih.gov/pubmed/9349340.

Dillon, S. R., Sprecher, C., Hammond, A., Bilsborough, J., Rosenfeld-Franklin, M., Presnell, S. R., et al. (2004). Interleukin 31, a cytokine produced by activated T cells, induces dermatitis in mice. Nat. Immunol. 5, 752–60. doi:10.1038/ni1084.

Ekholm, E., and Egelrud, T. (1999). Stratum corneum chymotryptic enzyme in psoriasis. Arch. Dermatol. Res. 291, 195–200. doi:10.1007/s004030050393.

Feng, J., Luo, J., Yang, P., Du, J., Kim, B. S., and Hu, H. (2018). Piezo2 channel–Merkel cell signaling modulates the conversion of touch to itch. Science (80-.). 360, 530–533. doi:10.1126/SCIENCE.AAR5703/SUPPL_FILE/AAR5703S2.MP4.

Fjellner, B., and Hagermark, O. (1979). Pruritus in polycythemia vera: treatment with aspirin and possibility of platelet involvement. Acta Derm. Venereol. 59, 505–512.

Fjellner, B., and Hagermark, O. (1981). Studies on pruritogenic and histamine-releasing effects of some putative peptide neurotransmitters. Acta Derm. Venereol. 61, 245–250. doi:10.2340/0001555561245250.

Furuno, T., Hagiyama, M., Sekimura, M., Okamoto, K., Suzuki, R., Ito, A., et al. (2012). Cell adhesion molecule 1 (CADM1) on mast cells promotes interaction with dorsal root ganglion neurites by heterophilic binding to nectin-3. J. Neuroimmunol. doi:10.1016/j.jneuroim.2012.05.016.

Gao, T., Ma, H., Xu, B., Bergman, J., Larhammar, D., and Lagerström, M. C. (2018). The Neuropeptide Y System Regulates Both Mechanical and Histaminergic Itch. J. Invest. Dermatol. 138, 2405–2411. doi:10.1016/J.JID.2018.05.008.

Grewe, M., Vogelsang, K., Ruzicka, T., Stege, H., and Krutmann, J. (2000). Neurotrophin-4 production by human epidermal keratinocytes: Increased expression in atopic dermatitis. J. Invest. Dermatol. 114, 1108–1112. doi:10.1046/j.1523-1747.2000.00974.x.

Guo, C. J., Mack, M. R., Oetjen, L. K., Trier, A. M., Council, M. L., Pavel, A. B., et al. (2020). Kallikrein 7 Promotes Atopic Dermatitis-Associated Itch Independently of Skin Inflammation. J. Invest. Dermatol. 140, 1244-1252.e4. doi:10.1016/j.jid.2019.10.022.

Hagermark, O., Hokfelt, T., and Pernow, B. (1978). Flare and itch induced by substance P in human skin. J. Invest. Dermatol. 71, 233–235. doi:10.1111/1523-1747.EP12515092.

Hagermark, O., and Strandberg, K. (1977). Pruritogenic activity of prostaglandin E2. Acta Derm. Venereol. 57, 37–43.

Hägermark, Ö., Strandberg, K., and Hamberg, M. (1977). Potentiation Of Itch And Flare Responses In Human Skin By Prostaglandins E2 And H2 and A Prostaglandin Endoperoxide Analog. J. Invest. Dermatol. 69, 527–530. doi:10.1111/1523-1747.EP12687966.

Hansson, L., Bäckman, A., Ny, A., Edlund, M., Ekholm, E., Hammarström, B. E., et al. (2002). Epidermal overexpression of stratum corneum chymotryptic enzyme in mice: A model for chronic itchy dermatitis. J. Invest. Dermatol. 118, 444–449. doi:10.1046/j.0022-202x.2001.01684.x.

Hashimoto, T., Ohata, H., and Momose, K. (2004). Itch-scratch responses induced by lysophosphatidic acid in mice. Pharmacology 72, 51–56. doi:10.1159/000078632.

Heuckeroth, R. O., Enomoto, H., Grider, J. R., Golden, J. P., Hanke, J. A., Jackman, A., et al. (1999). Gene targeting reveals a critical role for neurturin in the development and maintenance of enteric, sensory, and parasympathetic neurons. Neuron 22, 253–263. doi:10.1016/S0896-6273(00)81087-9.

Hosogi, M., Schmelz, M., Miyachi, Y., and Ikoma, A. (2006). Bradykinin is a potent pruritogen in atopic dermatitis: A switch from pain to itch. Pain 126, 16–23. doi:10.1016/J.PAIN.2006.06.003.

Imada, T., Komorita, N., Kobayashi, F., Naito, K., Yoshikawa, T., Miyazaki, M., et al. (2002). Therapeutic potential of a specific chymase inhibitor in atopic dermatitis. in Japanese Journal of Pharmacology (Jpn J Pharmacol), 214–217. doi:10.1254/jjp.90.214.

Imamachi, N., Goon, H. P., Lee, H., Anderson, D. J., Simon, M. I., Basbaum, A. I., et al. (2009). TRPV1-expressing primary afferents generate behavioral responses to pruritogens via multiple mechanisms. Proc. Natl. Acad. Sci. U. S. A. 106, 11330–11335. doi:10.1073/pnas.0905605106.

Inagaki, N., Nakamura, N., Nagao, M., Musoh, K., Kawasaki, H., and Nagai, H. (1999). Participation of histamine H1 and H2 receptors in passive cutaneous anaphylaxis-induced scratching behavior in ICR mice. Eur. J. Pharmacol. 367, 361–371. doi:10.1016/S0014-2999(98)00974-1.

Kakurai, M., Monteforte, R., Suto, H., Tsai, M., Nakae, S., and Galli, S. J. (2006). Mast Cell-Derived Tumor Necrosis Factor Can Promote Nerve Fiber Elongation in the Skin during Contact Hypersensitivity in Mice. Am. J. Pathol. 169, 1713–1721. doi:10.2353/AJPATH.2006.060602.

Katugampola, R., Church, M. K., and Clough, G. F. (2000). The Neurogenic Vasodilator Response to Endothelin-1: A Study in Human Skin In Vivo . Exp. Physiol. 85, 839–846. doi:10.1111/j.1469-445x.2000.02089.x.

Konda, D., Chandrashekar, L., Rajappa, M., Kattimani, S., Thappa, D. M., and Ananthanarayanan, P. H. (2015). Serotonin and interleukin-6: Association with pruritus severity, sleep quality and depression severity in Prurigo Nodularis. Asian J. Psychiatr. 17, 24–28. doi:10.1016/J.AJP.2015.07.010.

Kou, K., Nakamura, F., Aihara, M., Chen, H., Seto, K., Komuri-Yamaguchi, J., et al. (2012). Decreased expression of semaphorin-3A, a neurite-collapsing factor, is associated with itch in psoriatic skin. Acta Derm. Venereol. 92, 521–528. doi:10.2340/00015555-1350.

Lee, J., Kim, T., Hong, J., Woo, J., Min, H., Hwang, E., et al. (2012). Imiquimod enhances excitability of dorsal root ganglion neurons by inhibiting background (K 2P) and voltage-gated (K v1.1 and K v1.2) potassium channels. Mol. Pain 8. doi:10.1186/1744-8069-8-2.

Liu, Q., Sikand, P., Ma, C., Tang, Z., Han, L., Li, Z., et al. (2012). Mechanisms of Itch Evoked by β-Alanine. J. Neurosci. 32, 14532–14537. doi:10.1523/JNEUROSCI.3509-12.2012.

Liu, Q., Tang, Z., Surdenikova, L., Kim, S., Patel, K. N., Kim, A., et al. (2009). Sensory neuron-specific GPCR Mrgprs are itch receptors mediating chloroquine-induced pruritus. Cell 139, 1353–65. doi:10.1016/j.cell.2009.11.034.

Liu, Q., Weng, H.-J., Patel, K. N., Tang, Z., Bai, H., Steinhoff, M., et al. (2011). The distinct roles of two GPCRs, MrgprC11 and PAR2, in itch and hyperalgesia. Sci. Signal. 4, ra45. doi:10.1126/scisignal.2001925.

Liu, T., Xu, Z.-Z., Park, C.-K., Berta, T., and Ji, R.-R. (2010). Toll-like receptor 7 mediates pruritus. Nat. Neurosci. 13, 1460–1462. doi:10.1038/nn.2683.

Luo, J., Feng, J., Yu, G., Yang, P., Mack, M. R., Du, J., et al. (2018). Transient receptor potential vanilloid 4-expressing macrophages and keratinocytes contribute differentially to allergic and nonallergic chronic itch. J. Allergy Clin. Immunol. 141, 608----619.e7. doi:10.1016/j.jaci.2017.05.051.

Madej, A., Reich, A., Orda, A., and Szepietowski, J. C. (2007). Vascular adhesion protein-1 (VAP-1) is overexpressed in psoriatic patients. J. Eur. Acad. Dermatology Venereol. 21, 72–78. doi:10.1111/j.1468-3083.2006.01869.x.

McLatchie, L. M., Fraser, N. J., Main, M. J., Wise, A., Brown, J., Thompson, N., et al. (1998). RAMPs regulate the transport and ligand specificity of the calcitonin-receptor-like receptor. Nature 393, 333–339. doi:10.1038/30666.

Meixiong, J., Anderson, M., Limjunyawong, N., Sabbagh, M. F., Hu, E., Mack, M. R., et al. (2019a). Activation of Mast-Cell-Expressed Mas-Related G-Protein-Coupled Receptors Drives Non-histaminergic Itch. Immunity 50, 1163-1171.e5. doi:10.1016/j.immuni.2019.03.013.

Meixiong, J., Vasavda, C., Snyder, S. H., and Dong, X. (2019b). MRGPRX4 is a G protein-coupled receptor activated by bile acids that may contribute to cholestatic pruritus. Proc. Natl. Acad. Sci. U. S. A. 116, 10525–10530. doi:10.1073/PNAS.1903316116/SUPPL_FILE/PNAS.1903316116.SAPP.PDF.

Miao, X., Huang, Y., Liu, T. T., Guo, R., Wang, B., Wang, X. L., et al. (2018). TNF-α/TNFR1 Signaling is Required for the Full Expression of Acute and Chronic Itch in Mice via Peripheral and Central Mechanisms. Neurosci. Bull. 34, 42–53. doi:10.1007/S12264-017-0124-3/METRICS.

Min, H., Lee, H., Lim, H., Jang, Y. H., Chung, S. J., Lee, C. J., et al. (2014). TLR4 enhances histamine-mediated pruritus by potentiating TRPV1 activity. Mol. Brain 7, 59. doi:10.1186/s13041-014-0059-9.

Mizukawa, Y., Doi, T., Yamazaki, Y., Kudo, A., and Shiohara, T. (2016). Epidermal Neuromedin U Attenuates IgE-Mediated Allergic Skin Inflammation. PLoS One 11, e0160122. doi:10.1371/journal.pone.0160122.

Nakamura, M., Toyoda, M., and Morohashi, M. (2003). Pruritogenic mediators in psoriasis vulgaris: comparative evaluation of itch-associated cutaneous factors. Br. J. Dermatol. 149, 718–730. Available at: http://www.ncbi.nlm.nih.gov/pubmed/14616362.

Nattkemper, L. A., Tey, H. L., Valdes-Rodriguez, R., Lee, H., Mollanazar, N. K., Albornoz, C., et al. (2018). The Genetics of Chronic Itch: Gene Expression in the Skin of Patients with Atopic Dermatitis and Psoriasis with Severe Itch. J. Invest. Dermatol. 138, 1311–1317. doi:10.1016/j.jid.2017.12.029.

Neisius, U., Olssonb, R., Rukwied, R., Lischetzki, G., and Schmelz, M. (2002). Prostaglandin E2 induces vasodilation and pruritus, but no protein extravasation in atopic dermatitis and controls. J. Am. Acad. Dermatol. 47, 28–32. doi:10.1067/MJD.2002.120462.

Nigam, R., El-Nour, H., Amatya, B., and Nordlind, K. (2010). GABA and GABAA receptor expression on immune cells in psoriasis: A pathophysiological role. Arch. Dermatol. Res. 302, 507–515. doi:10.1007/s00403-010-1052-5.

Nojima, H., and Carstens, E. (2003). 5-Hydroxytryptamine (5-HT)2 receptor involvement in acute 5-HT-evoked scratching but not in allergic pruritus induced by dinitrofluorobenzene in rats. J. Pharmacol. Exp. Ther. 306, 245–252. doi:10.1124/jpet.103.049239.

Oetjen, L. K., Mack, M. R., Feng, J., Whelan, T. M., Niu, H., Guo, C. J., et al. (2017). Sensory Neurons Co-opt Classical Immune Signaling Pathways to Mediate Chronic Itch. Cell. doi:10.1016/j.cell.2017.08.006.

Oh, M.-H., Oh, S. Y., Lu, J., Lou, H., Myers, A. C., Zhu, Z., et al. (2013). TRPA1-dependent pruritus in IL-13-induced chronic atopic dermatitis. J. Immunol. 191, 5371–5382. doi:10.4049/jimmunol.1300300.

Oikonomopoulou, K., Hansen, K. K., Saifeddine, M., Vergnolle, N., Tea, I., Blaber, M., et al. (2006). Kallikrein-mediated cell signalling: Targeting proteinase-activated receptors (PARs). in Biological Chemistry (Biol Chem), 817–824. doi:10.1515/BC.2006.104.

Patricio, E. S., Costa, R., Figueiredo, C. P., Gers-Barlag, K., Bicca, M. A., Manjavachi, M. N., et al. (2015). Mechanisms Underlying the Scratching Behavior Induced by the Activation of Proteinase-Activated Receptor-4 in Mice. J. Invest. Dermatol. 135, 2484–2491.

Qu, L., Fan, N., Ma, C., Wang, T., Han, L., Fu, K., et al. (2014). Enhanced excitability of MRGPRA3- and MRGPRD-positive nociceptors in a model of inflammatory itch and pain. Brain 137, 1039–50. doi:10.1093/brain/awu007.

Reddy, V. B., Sun, S., Azimi, E., Elmariah, S. B., Dong, X., and Lerner, E. A. (2015). Redefining the concept of protease-activated receptors: cathepsin S evokes itch via activation of Mrgprs. Nat. Commun. 6, 7864. doi:10.1038/ncomms8864.

Reich, A., Orda, A., Wiśnicka, B., and Szepietowski, J. C. (2007). Plasma neuropeptides and perception of pruritus in psoriasis. Acta Derm. Venereol. 87, 299–304. doi:10.2340/00015555-0265.

Rogoz, K., Andersen, H. H., Lagerström, M. C., and Kullander, K. (2014). Multimodal use of calcitonin gene-related peptide and substance P in itch and acute pain uncovered by the elimination of vesicular glutamate transporter 2 from transient receptor potential cation channel subfamily v member 1 neurons. J. Neurosci. 34, 14055–14068. doi:10.1523/JNEUROSCI.1722-14.2014.

Rossbach, K., Nassenstein, C., Gschwandtner, M., Schnell, D., Sander, K., Seifert, R., et al. (2011). Histamine H1, H3 and H4 receptors are involved in pruritus. Neuroscience 190, 89–102. doi:10.1016/J.NEUROSCIENCE.2011.06.002.

Rossbach, K., Wendorff, S., Sander, K., Stark, H., Gutzmer, R., Werfel, T., et al. (2009). Histamine H4 receptor antagonism reduces hapten-induced scratching behaviour but not inflammation. Exp. Dermatol. 18, 57–63. doi:10.1111/j.1600-0625.2008.00762.x.

Sakai, K., Sanders, K. M., Youssef, M. R., Yanushefski, K. M., Jensen, L. E., Yosipovitch, G., et al. (2017). Role of neurturin in spontaneous itch and increased nonpeptidergic intraepidermal fiber density in a mouse model of psoriasis. Pain 158, 2196–2202. doi:10.1097/j.pain.0000000000001025.

Schüttenhelm, B. N., Duraku, L. S., Dijkstra, J. F., Walbeehm, E. T., and Holstege, J. C. (2015). Differential Changes in the Peptidergic and the Non-Peptidergic Skin Innervation in Rat Models for Inflammation, Dry Skin Itch, and Dermatitis. J. Invest. Dermatol. 135, 2049–2057. doi:10.1038/JID.2015.137.

Shimizu, Y., Sonoda, A., Nogi, C., Ogushi, Y., Kanda, R., Yamaguchi, S., et al. (2014). B-type (brain) natriuretic peptide and pruritus in hemodialysis patients. Int. J. Nephrol. Renovasc. Dis. 7, 329–335. doi:10.2147/IJNRD.S65929.

Shiratori-Hayashi, M., Hasegawa, A., Toyonaga, H., Andoh, T., Nakahara, T., Kido-Nakahara, M., et al. (2019). Role of P2X3 receptors in scratching behavior in mouse models. J. Allergy Clin. Immunol. 143, 1252-1254.e8. doi:10.1016/j.jaci.2018.10.053.

Sikand, P., Dong, X., and LaMotte, R. H. (2011). BAM8-22 peptide produces itch and nociceptive sensations in humans independent of histamine release. J. Neurosci. 31, 7563–7. doi:10.1523/JNEUROSCI.1192-11.2011.

Solinski, H. J., Kriegbaum, M. C., Tseng, P. Y., Earnest, T. W., Gu, X., Barik, A., et al. (2019). Nppb Neurons Are Sensors of Mast Cell-Induced Itch. Cell Rep. 26, 3561-3573.e4. doi:10.1016/j.celrep.2019.02.089.

Sonkoly, E., Muller, A., Lauerma, A. I., Pivarcsi, A., Soto, H., Kemeny, L., et al. (2006). IL-31: a new link between T cells and pruritus in atopic skin inflammation. J. Allergy Clin. Immunol. 117, 411–417. doi:10.1016/j.jaci.2005.10.033.

Stantcheva, K. K., Iovino, L., Dhandapani, R., Martinez, C., Castaldi, L., Nocchi, L., et al. (2016). A subpopulation of itch-sensing neurons marked by Ret and somatostatin expression. EMBO Rep. 17, 585–600. doi:10.15252/embr.201540983.

Stefansson, K., Brattsand, M., Roosterman, D., Kempkes, C., Bocheva, G., Steinhoff, M., et al. (2008). Activation of proteinase-activated receptor-2 by human kallikrein-related peptidases. J. Invest. Dermatol. 128, 18–25. doi:10.1038/sj.jid.5700965.

Steinhoff, M., Bienenstock, J., Schmelz, M., Maurer, M., Wei, E., and Bíró, T. (2006). Neurophysiological, neuroimmunological, and neuroendocrine basis of pruritus. J. Invest. Dermatol. 126, 1705–1718. doi:10.1038/sj.jid.5700231.

Steinhoff, M., Neisius, U., Ikoma, A., Fartasch, M., Heyer, G., Skov, P. S., et al. (2003). Proteinase-activated receptor-2 mediates itch: a novel pathway for pruritus in human skin. J. Neurosci. 23, 6176–6180. doi:23/15/6176 [pii].

Stokes, A. J., Shimoda, L. M. N., Koblan-Huberson, M., Adra, C. N., and Turner, H. (2004). A TRPV2-PKA signaling module for transduction of physical stimuli in mast cells. J. Exp. Med. 200, 137–147. doi:10.1084/jem.20032082.

Sugimoto, M., Arai, I., Futaki, N., Honma, Y., Sakurai, T., Hashimoto, Y., et al. (2007). Putative mechanism of the itch-scratch circle: Repeated scratching decreases the cutaneous level of prostaglandin D2, a mediator that inhibits itching. Prostaglandins Leukot. Essent. Fat. Acids 76, 93–101. doi:10.1016/j.plefa.2006.11.007.

Takai, Y., Hiramoto, K., Nishimura, Y., and Ooi, K. (2017). Relationship between biochemical factors and skin symptoms in chronic venous disease. Arch. Dermatol. Res. 309, 253–258. doi:10.1007/s00403-017-1721-8.

Takano, N., Sakurai, T., and Kurachi, M. (2005). Effects of Anti-Nerve Growth Factor Antibody on Symptoms in the NC/Nga Mouse, an Atopic Dermatitis Model. J. Pharmacol. Sci. 99, 277–286. doi:10.1254/jphs.fp0050564.

Taneda, K., Tominaga, M., Negi, O., Tengara, S., Kamo, A., Ogawa, H., et al. (2011). Evaluation of epidermal nerve density and opioid receptor levels in psoriatic itch. Br. J. Dermatol. 165, 277–284. doi:10.1111/j.1365-2133.2011.10347.x.

Terakawa, M., Fujieda, Y., Tomimori, Y., Muto, T., Tanaka, T., Maruoka, H., et al. (2008). Oral chymase inhibitor SUN13834 ameliorates skin inflammation as well as pruritus in mouse model for atopic dermatitis. Eur. J. Pharmacol. 601, 186–191. doi:10.1016/j.ejphar.2008.10.040.

Thomsen, J. S., Petersen, M. B., Benfeldt, E., Jensen, S. B., and Serup, J. (2001). Scratch induction in the rat by intradermal serotonin: A model for pruritus. Acta Derm. Venereol. 81, 250–254. doi:10.1080/00015550152572868.

Thomsen, J. S., Sonne, M., Benfeldt, E., Jensen, S. B., Serup, J., and Menne, T. (2002). Experimental itch in sodium lauryl sulphate-inflamed and normal skin in humans: A randomized, double-blind, placebo-controlled study of histamine and other inducers of itch. Br. J. Dermatol. 146, 792–800. doi:10.1046/j.1365-2133.2002.04722.x.

Tsujii, K., Andoh, T., Lee, J.-B., and Kuraishi, Y. (2008). Activation of Proteinase-Activated Receptors Induces Itch-Associated Response Through Histamine-Dependent and -Independent Pathways in Mice. J. Pharmacol. Sci. 108, 385–388. doi:10.1254/jphs.08200SC.

Tsujii, K., Andoh, T., Ui, H., Lee, J.-B., and Kuraishi, Y. (2009). Involvement of Tryptase and Proteinase-Activated Receptor-2 in Spontaneous Itch-Associated Response in Mice With Atopy-like Dermatitis. J. Pharmacol. Sci. 109, 388–395. Available at: http://www.ncbi.nlm.nih.gov/pubmed/19270428.

Ui, H., Andoh, T., Lee, J.-B., Nojima, H., and Kuraishi, Y. (2006). Potent pruritogenic action of tryptase mediated by PAR-2 receptor and its involvement in anti-pruritic effect of nafamostat mesilate in mice. Eur. J. Pharmacol. 530, 172–178. doi:10.1016/j.ejphar.2005.11.021.

Wan, L., Jin, H., Liu, X. Y., Jeffry, J., Barry, D. M., Shen, K. F., et al. (2017). Distinct roles of NMB and GRP in itch transmission. Sci. Reports 2017 71 7, 1–16. doi:10.1038/s41598-017-15756-0.

Weisshaar, E., Ziethen, B., Röhl, F. W., and Gollnick, H. (1999). The antipruritic effect of a 5-HT3 receptor antagonist (tropisetron) is dependent on mast cell depletion - An experimental study. Exp. Dermatol. 8, 254–260. doi:10.1111/j.1600-0625.1999.tb00379.x.

Wenzel, R. R., Zbinden, S., Noll, G., Meier, B., and Lüscher, T. F. (1998). Endothelin-1 induces vasodilation in human skin by nociceptor fibres and release of nitric oxide. Br. J. Clin. Pharmacol. 45, 441–446. doi:10.1046/j.1365-2125.1998.00703.x.

Wilson, S. R., Gerhold, K. A., Bifolck-Fisher, A., Liu, Q., Patel, K. N., Dong, X., et al. (2011). TRPA1 is required for histamine-independent, Mas-related G protein-coupled receptor-mediated itch. Nat. Neurosci. 14, 595–602. doi:10.1038/nn.2789.

Wilson, S. R., Thé, L., Batia, L. M., Beattie, K., Katibah, G. E., McClain, S. P., et al. (2013). The epithelial cell-derived atopic dermatitis cytokine TSLP activates neurons to induce itch. Cell 155, 285–295. doi:10.1016/j.cell.2013.08.057.

Yamaguchi, T., Nagasawa, T., Satoh, M., and Kuraishi, Y. (1999). Itch-associated response induced by intradermal serotonin through 5-HT2 receptors in mice. Neurosci. Res. 35, 77–83. doi:10.1016/S0168-0102(99)00070-X.

Yoshioka, T., Imura, K., Asakawa, M., Suzuki, M., Oshima, I., Hirasawa, T., et al. (2009). Impact of the Gly573Ser substitution in TRPV3 on the development of allergic and pruritic dermatitis in mice. J. Invest. Dermatol. 129, 714–722. doi:10.1038/jid.2008.245.

Yu, H., Zhao, T., Liu, S., Wu, Q., Johnson, O., Wu, Z., et al. (2019). MRGPRX4 is a bile acid receptor for human cholestatic itch. Elife 8. doi:10.7554/ELIFE.48431.

Zhang, L., Jiang, G. Y., Song, N. J., Huang, Y., Chen, J. Y., Wang, Q. X., et al. (2014). Extracellular signal-regulated kinase (ERK) activation is required for itch sensation in the spinal cord. Mol. Brain 7. doi:10.1186/1756-6606-7-25.

Zhao, J., Munanairi, A., Liu, X. Y., Zhang, J., Hu, L., Hu, M., et al. (2020). PAR2 Mediates Itch via TRPV3 Signaling in Keratinocytes. J. Invest. Dermatol. 140, 1524–1532. doi:10.1016/J.JID.2020.01.012.

Zheng, T., Oh, M. H., Oh, S. Y., Schroeder, J. T., Glick, A. B., and Zhu, Z. (2009). Transgenic Expression of Interleukin-13 in the Skin Induces a Pruritic Dermatitis and Skin Remodeling. J. Invest. Dermatol. 129, 742–751. doi:10.1038/JID.2008.295.

Zhu, Y., Pan, W. H., Wang, X. R., Liu, Y., Chen, M., Xu, X. G., et al. (2015). Tryptase and protease-activated receptor-2 stimulate scratching behavior in a murine model of ovalbumin-induced atopic-like dermatitis. Int. Immunopharmacol. 28, 507–512. doi:10.1016/j.intimp.2015.04.047.

Supplementary Table S10. Expression of itch-associated genes in mast cells isolated from involved psoriatic skin and their expression in datasets GDS4602, GSE80047, and GSE7809. Values marked in red indicate genes with p value adjusted for false discovery rate (q) < 0.05. NA is not applicable/not available.

		Isolated MCs			GDS4602			GSE78097			GSE80047		
**Category**	**Gene Symbol**	***log2FC***	***p value***	***q value***	***log2FC***	***p value***	***q value***	***log2FC***	***p value***	***q value***	***log2FC***	***p value***	***q value***
Cell adhesion molecule	***AOC3***	1.948225	0.062511	0.289318	-0.70843	1.22E-12	7.02E-12	-1.10886	0.024022	0.083327	-0.233802	1.00E-06	3.99E-06
Kinin autacoid receptor	***BDKRB1***	0.657231	0.212259	0.464428	0.363231	7.43E-14	4.67E-13	1.546137	8.47E-05	0.000811	0.251533	7.61E-12	9.26E-11
Kinin autacoid receptor	***BDKRB2***	0.134842	0.891286	0.946325	0.471043	2.11E-13	1.28E-12	0.938355	0.002655	0.013445	0.053201	0.016798	0.028577
Cell adhesion molecule	***CADM1***	0.047191	0.963785	0.983646	NA	NA	NA	NA	NA	NA	-0.011507	0.41113	0.479864
Neuropeptide	***CALCA***	0.223845	0.850124	0.924505	NA	NA	NA	NA	NA	NA	0.003467	0.796637	0.833929
Neuropeptide receptor	***CALCRL***	1.710243	0.112717	0.372069	NA	NA	NA	NA	NA	NA	0.017539	0.624532	0.683377
Chemokine	***CCL1***	NA	NA	NA	0.139733	1.08E-05	3.14E-05	0.048454	0.483861	0.832112	0.004041	0.873201	0.897529
Chemokine	***CCL14***	-2.59412	0.009749	0.157004	NA	NA	NA	NA	NA	NA	NA	NA	NA
Chemokine	***CCL15-CCL14///CCL14***	NA	NA	NA	-0.94391	2.39E-16	1.83E-15	-0.56949	0.155357	0.348335	NA	NA	NA
Chemokine	***CCL15-CCL14///CCL15///CCL14***	NA	NA	NA	NA	NA	NA	NA	NA	NA	-0.135182	2.73E-05	8.17E-05
Chemokine	***CCL17***	0.0489	0.936155	0.9682	0.29395	9.39E-08	3.5E-07	0.392604	0.57046	0.90092	0.072991	0.124019	0.169634
Chemokine	***CCL18***	0.699165	0.16898	0.428651	NA	NA	NA	NA	NA	NA	1.021609	1.96E-15	6.05E-14
Chemokine	***CCL2***	1.107776	0.295525	0.554585	1.25902	9.79E-19	9.15E-18	1.151915	0.000709	0.004629	0.388019	2.04E-09	1.42E-08
Chemokine	***CCL20***	1.836001	0.041572	0.255626	2.968398	1.80E-44	2.25E-42	6.102654	1.31E-08	1.04E-06	1.234453	5.87E-19	5.45E-17
Cytokine	***CCL26***	NA	NA	NA	-0.02477	0.560825	0.626521	-0.10884	0.880008	1	-0.196297	7.51E-05	0.000205
Chemokine	***CCL27***	-1.31221	0.043234	0.259009	-2.94934	9.11E-39	5.81E-37	-2.51549	4.18E-06	7.52E-05	-0.387256	4.15E-10	3.37E-09
Chemokine	***CCL3***	1.682304	0.028739	0.222687	NA	NA	NA	NA	NA	NA	NA	NA	NA
Chemokine	***CCL3L3///CCL3L1///CCL3***	NA	NA	NA	0.97173	1.03E-13	6.44E-13	2.903965	6.94E-06	0.000112	0.585391	1.10E-17	6.49E-16
Chemokine	***CCL4***	1.134742	0.101704	0.352912	0.87589	2.26E-23	3.17E-22	2.716475	2.12E-05	0.000266	0.381216	5.39E-19	5.11E-17
Chemokine	***CCL7***	NA	NA	NA	0.403647	1.53E-19	1.54E-18	0.697782	0.002132	0.011303	0.38793	1.57E-17	8.81E-16
Chemokine	***CCL8***	0.453516	0.369958	0.601318	1.007746	1.04E-15	7.52E-15	2.810235	3.79E-05	0.000424	0.677027	8.04E-15	2.15E-13
Neurotransmitter precursor processor	***CHAT***	-0.03846	0.966679	0.985519	0.044586	0.174257	0.229743	0	1	1	0.05623	0.003181	0.006291
Muscarinic receptor	***CHRM1***	-0.65146	0.605151	0.77957	-0.26429	1.97E-09	8.64E-09	-0.97572	0.001601	0.008927	-0.208856	4.73E-06	1.64E-05
Muscarinic receptor	***CHRM3***	1.982842	0.069583	0.298701	NA	NA	NA	NA	NA	NA	-0.211707	1.09E-05	3.52E-05
Ion channel	***CHRNA9***	1.033663	0.228359	0.482965	2.006807	2.41E-29	5.9E-28	6.82556	8.45E-14	1.06E-10	1.040059	3.61E-13	6.17E-12
Ion channel	***CHRNB3***	0.911333	0.31242	0.571331	NA	NA	NA	0	1	1	0.083708	6.13E-07	2.55E-06
Protease	***CMA1***	NA	NA	NA	-0.3095	0.006717	0.012487	-0.11585	0.753192	1	-0.194676	0.000732	0.001649
Cannabinoid receptor	***CNR1***	1.675035	0.058643	0.284238	NA	NA	NA	NA	NA	NA	0.077538	0.000417	0.000985
Cannabinoid receptor	***CNR2***	2.012549	0.085555	0.324844	0.052999	0.143528	0.193633	0	1	1	0.124703	1.75E-08	1E-07
Cytokine receptor	***CRLF2***	NA	NA	NA	0.185443	2.59E-07	9.16E-07	0.263441	0.234112	0.486344	0.287131	6.27E-14	1.3E-12
Protease	***CTSB***	2.899804	0.000106	0.030081	NA	NA	NA	NA	NA	NA	0.083676	1.26E-06	4.91E-06
Protease	***CTSF***	1.835881	0.076549	0.309877	-0.57996	2.35E-24	3.61E-23	-1.66688	3.27E-05	0.000377	-0.319692	1.91E-17	1.05E-15
Protease	***CTSG***	-0.1474	0.853086	0.925719	-0.43349	1.72E-06	5.52E-06	-0.35284	0.329823	0.638156	-0.286953	1.21E-06	4.71E-06
Protease	***CTSH***	1.137557	0.316245	0.574724	-0.11566	0.059012	0.088146	-0.31091	0.158581	0.354191	-0.03896	0.043235	0.066621
Protease	***CTSS***	-0.49838	0.597606	0.774177	NA	NA	NA	NA	NA	NA	0.305551	1.45E-12	2.11E-11
Protease	***CTSZ***	0.831505	0.302866	0.561729	NA	NA	NA	NA	NA	NA	-0.02679	0.182618	0.238432
Chemokine	***CXCL1***	0.501782	0.400558	0.624638	2.53738	4.31E-28	9.23E-27	6.014307	4.97E-07	1.44E-05	1.333685	9.18E-20	1.2E-17
Chemokine	***CXCL10***	1.404747	0.055614	0.27934	2.818208	2.24E-30	6.09E-29	6.349982	1.84E-09	2.41E-07	1.417416	8.79E-16	2.97E-14
Chemokine	***CXCL11***	1.152918	0.05232	0.275008	NA	NA	NA	NA	NA	NA	0.511822	1.40E-11	1.59E-10
Chemokine	***CXCL3***	-0.8307	0.448935	0.662103	0.286704	2.35E-14	1.53E-13	3.034148	3.45E-08	1.97E-06	0.171358	4.24E-05	0.000122
Chemokine	***CXCL8***	0.430815	0.473225	0.682122	NA	NA	NA	NA	NA	NA	0.133603	5.02E-05	0.000142

Chemokine receptor	*CXCR1*	1.010806	0.218667	0.471447	0.118489	0.000101	0.00026	-0.00067	0.491508	0.832112	0.068876	0.001182	0.002551
Chemokine receptor	*CXCR3*	1.417543	0.163383	0.424884	NA	NA	NA	NA	NA	NA	0.077162	8.77E-06	2.89E-05
Neurotransmitter precursor processor	*DDC*	0.690746	0.259681	0.516531	NA	NA	NA	NA	NA	NA	-0.080731	0.131604	0.178704
Endopeptidase	*ECEL1*	1.492537	0.030504	0.22794	0.139428	0.00128	0.002726	0	1	1	0.06373	6.02E-06	2.05E-05
Vasoconstrictor	*EDN1*	0.402145	0.79734	0.894667	NA	NA	NA	NA	NA	NA	-0.031962	0.176865	0.231652
Vasoconstrictor	*EDN2*	-0.82357	0.380599	0.608415	0.125332	0.000619	0.001391	-0.02977	0.205927	0.437193	-0.042226	0.080746	0.115864
Vasoconstrictor	*EDN3*	0.959144	0.138915	0.404173	NA	NA	NA	NA	NA	NA	-0.097653	0.019369	0.032452
Vasoconstrictor receptor	*EDNRA*	0.69453	0.523898	0.722108	NA	NA	NA	NA	NA	NA	-0.095074	0.001752	0.003651
Protease	*ELANE*	-0.85935	0.12785	0.391739	-0.38933	0.002523	0.005081	-0.17725	0.633393	0.979607	-0.080384	0.043455	0.066932
Proteinase activated receptor	*F2R*	-1.94496	0.024148	0.207228	NA	NA	NA	NA	NA	NA	-0.033641	0.114345	0.15793
Proteinase activated receptor	*F2RL1*	1.245995	0.013911	0.173042	NA	NA	NA	NA	NA	NA	0.074976	0.235853	0.298219
Proteinase activated receptor	*F2RL2*	3.047573	0.001113	0.07715	NA	NA	NA	NA	NA	NA	0.14281	0.000259	0.000639
Proteinase activated receptor	*F2RL3*	1.951297	0.008419	0.148714	0.080535	7.29E-05	0.000191	0.002738	0.398657	0.729912	-0.005197	0.863375	0.89001
Neurotransmitter receptor	*GABRA1*	1.511628	0.126321	0.390863	NA	NA	NA	0	1	1	0.079024	0.001254	0.002691
Neurotransmitter receptor	*GABRA2*	1.305152	0.332379	0.587267	NA	NA	NA	NA	NA	NA	-0.017341	0.320416	0.387651
Neurotransmitter receptor	*GABRA3*	0.313064	0.658845	0.812804	0.017468	0.435468	0.505756	0.033122	0.276883	0.556528	0.02221	0.265825	0.329761
Neurotransmitter receptor	*GABRA4*	1.535099	0.0521	0.274768	NA	NA	NA	NA	NA	NA	0.388673	1.10E-09	8.12E-09
Neurotransmitter receptor	*GABRA5*	0.038397	0.954812	0.978366	NA	NA	NA	NA	NA	NA	0.042233	0.007118	0.013092
Neurotransmitter receptor	*GABRA6*	1.700464	0.005103	0.125058	0.067065	0.002644	0.005304	0	1	1	0.028398	0.084819	0.121024
Neurotrophic factor receptor	*GFRA1*	2.623059	0.028995	0.223645	NA	NA	NA	NA	NA	NA	-0.290977	2.88E-08	1.56E-07
Neurotrophic factor receptor	*GFRA2*	1.43731	0.153788	0.41824	NA	NA	NA	NA	NA	NA	-0.046385	0.064065	0.094328
Neurotrophic factor receptor	*GFRA3*	-0.01369	0.979983	0.989979	NA	NA	NA	NA	NA	NA	-0.121104	0.012846	0.022389
Neurotrophic factor receptor	*GFRA4*	0.606424	0.349308	0.597553	NA	NA	NA	0	1	1	-0.001022	0.938653	0.951171
Neuropeptide receptor	*GPBAR1*	-0.15174	0.89822	0.950325	0.043998	0.191141	0.248871	0.007938	0.457097	0.809461	0.022977	0.467641	0.535692
Neuropeptide	*GRP*	0.606435	0.382847	0.610238	-0.07326	0.085026	0.12197	-0.02366	0.146557	0.333763	-0.008848	0.862237	0.889053
Neuropeptide receptor	*GRPR*	NA	NA	NA	-0.05651	0.10305	0.14467	0	1	1	0.04918	0.011096	0.019624
Amine-processing enzyme	*HDC*	0.186993	0.823656	0.909647	-0.4051	4.79E-08	1.83E-07	2.082389	0.000414	0.002994	0.139637	0.036416	0.057109
Amine receptor	*HRH1*	2.651148	0.028066	0.220257	NA	NA	NA	NA	NA	NA	0.065779	0.068981	0.100828
Amine receptor	*HRH2*	-0.31711	0.712076	0.845394	0.313113	7.59E-11	3.76E-10	-0.00097	0.919974	1	0.024421	0.327454	0.395276
Amine receptor	*HRH3*	-1.59087	0.07448	0.305864	NA	NA	NA	0	1	1	0.046966	0.000327	0.00079
Amine receptor	*HRH4*	1.948989	0.138745	0.403971	NA	NA	NA	0	1	1	0.108591	2.64E-09	1.79E-08
Neurotransmitter receptor	*HTR2A*	1.965816	0.044448	0.261766	NA	NA	NA	NA	NA	NA	-0.150005	4.30E-05	0.000123
Neurotransmitter receptor	*HTR2B*	1.732327	0.127005	0.391137	0.036102	0.547575	0.613497	-0.02097	0.915589	1	0.238513	3.03E-06	1.09E-05
Neurotransmitter receptor	*HTR2C*	1.303776	0.139256	0.40427	NA	NA	NA	0	1	1	0.035807	0.004124	0.00797
Neurotransmitter receptor	*HTR3A*	2.746894	0.008607	0.150244	NA	NA	NA	NA	NA	NA	0.321569	3.82E-10	3.12E-09
Neurotransmitter receptor	*HTR3B*	2.151091	0.005266	0.126976	0.130776	0.000114	0.000292	0.042946	0.497874	0.832112	0.164736	3.02E-08	1.63E-07
Neurotransmitter receptor	*HTR3C*	-1.65238	0.106972	0.361731	0.072216	0.022221	0.0368	0	1	1	0.028235	0.08495	0.121172
Neurotransmitter receptor	*HTR7*	0.242115	0.794008	0.892456	NA	NA	NA	NA	NA	NA	0.089605	1.11E-05	3.58E-05
Neurotransmitter receptor	*HTR7P1///HTR7*	NA	NA	NA	0.073282	0.00301	0.00599	0	1	1	-0.116356	0.002203	0.004498
Cytokine	*IFNG*	NA	NA	NA	0.479719	2.24E-26	4.09E-25	0.622923	0.047862	0.147201	0.458377	6.60E-18	4.21E-16
Cytokine	*IL10*	0.261366	0.642564	0.803624	0.111987	1.13E-05	3.28E-05	0.000131	0.512569	0.832112	0.142814	7.42E-08	3.7E-07
Cytokine	*IL13*	1.687907	0.064054	0.291907	0.167366	4.49E-05	0.00012	0.021877	0.486561	0.832112	-0.084367	0.000982	0.002158
Cytokine receptor	*IL13RA1*	3.307116	0.002169	0.093875	NA	NA	NA	NA	NA	NA	0.046435	0.038672	0.06032
Cytokine receptor	*IL13RA2*	NA	NA	NA	-0.02023	0.62059	0.682471	0.361828	0.186841	0.403815	-0.026293	0.69743	0.747978
Cytokine	*IL17A*	1.235261	0.20236	0.455363	NA	NA	NA	NA	NA	NA	0.294933	3.16E-19	3.27E-17
Cytokine	*IL17F*	0.472743	0.369432	0.601318	0.302845	1.07E-08	4.34E-08	0.670358	0.120551	0.306235	0.288279	4.03E-09	2.63E-08

Cytokine	***IL19***	0.128025	0.904318	0.95348	1.912114	4.33E-25	7.11E-24	5.072069	5.95E-05	0.000612	0.995973	5.07E-24	3.36E-21
Cytokine	***IL2***	1.152907	0.052325	0.275008	NA	NA	NA	0	1	1	0.043512	0.010633	0.018875
Cytokine	***IL20***	0.172588	0.853297	0.925728	1.092522	4.12E-29	9.67E-28	2.128719	0.004345	0.020126	0.485335	1.06E-21	2.9E-19
Cytokine	***IL22***	0.175301	0.762838	0.87672	NA	NA	NA	NA	NA	NA	0.14635	1.73E-12	2.47E-11
Cytokine	***IL23A***	1.695361	0.015433	0.178366	NA	NA	NA	NA	NA	NA	0.165154	3.13E-11	3.28E-10
Cytokine	***IL23A///TRBV19***	NA	NA	NA	0.420768	1.26E-20	1.4E-19	-0.00311	0.956381	1	0.17868	4.06E-09	2.64E-08
Cytokine	***IL26***	NA	NA	NA	0.400025	5.92E-22	7.39E-21	4.988873	1.90E-10	3.63E-08	0.749544	1.04E-20	1.91E-18
Cytokine receptor	***IL2RA***	0.184743	0.895331	0.948769	NA	NA	NA	NA	NA	NA	0.141256	1.39E-09	1.01E-08
Cytokine receptor	***IL2RB***	4.162673	0.002488	0.098035	0.194622	0.002054	0.004209	-1.32503	0.007382	0.031176	0.054057	0.130754	0.177692
Cytokine	***IL31***	-0.67511	0.348807	0.597553	NA	NA	NA	NA	NA	NA	NA	NA	NA
Cytokine receptor	***IL31RA***	0.614046	0.247212	0.504043	NA	NA	NA	NA	NA	NA	0.041572	0.026954	0.043569
Cytokine	***IL36A***	-0.08958	0.915581	0.95844	1.940991	2.69E-29	6.46E-28	5.438729	3.32E-06	6.29E-05	0.525378	3.80E-17	1.91E-15
Cytokine	***IL36G***	-0.44902	0.684316	0.828122	5.183424	1.13E-83	4.06E-80	4.815537	7.40E-11	1.86E-08	1.084133	7.67E-17	3.47E-15
Cytokine	***IL4***	NA	NA	NA	NA	NA	NA	NA	NA	NA	-0.004945	0.802056	0.838361
Cytokine receptor	***IL4R***	-1.19541	0.162265	0.423974	1.122413	5.10E-44	5.92E-42	2.057644	9.67E-07	2.41E-05	0.258318	4.96E-14	1.06E-12
Cytokine	***IL6***	0.384028	0.616279	0.787284	0.454902	6.23E-15	4.24E-14	0.25129	0.407343	0.741484	0.24837	1.02E-08	6.12E-08
Cytokine receptor	***IL6R***	-0.63937	0.2349	0.490574	NA	NA	NA	NA	NA	NA	0.015423	0.518902	0.584965
Cytokine	***IL7***	0.840512	0.127248	0.391137	-0.34567	0.000227	0.000552	-0.8983	8.32E-06	0.00013	0.045707	0.28379	0.348672
Cytokine	***IL9***	NA	NA	NA	0.03015	0.096346	0.136429	0.000123	0.512569	0.832112	0.00403	0.876511	0.90017
Tyrosine kinase	***JAK1***	2.451033	0.005061	0.124806	NA	NA	NA	NA	NA	NA	-0.190959	1.91E-15	5.93E-14
Voltage gated potassium channel	***KCNA1***	1.08914	0.298314	0.557211	NA	NA	NA	NA	NA	NA	-0.152932	0.004648	0.008886
Voltage gated potassium channel	***KCNA2***	-1.74753	0.014542	0.174152	NA	NA	NA	NA	NA	NA	0.021783	0.442497	0.511058
Voltage gated Potassium channel	***KCNK2***	0.891809	0.387463	0.61415	-0.58418	5.31E-08	2.02E-07	-1.90293	0.006497	0.027928	-0.199236	0.004679	0.008938
Potassium channel	***KCNK4***	-0.55744	0.422005	0.641995	0.125705	0.006595	0.012274	0	1	1	-0.038117	0.039607	0.061621
Protease	***KLK13***	0.448375	0.532023	0.728337	NA	NA	NA	NA	NA	NA	0.862158	1.63E-22	5.76E-20
Protease	***KLK14***	NA	NA	NA	0.177861	0.001316	0.002796	-0.13544	0.314838	0.615068	-0.072531	0.056777	0.084909
Protease	***KLK5***	2.227567	0.066977	0.29549	0.143399	0.126911	0.174045	0.068974	0.670032	1	0.210531	5.08E-05	0.000144
Protease	***KLK6***	-0.17397	0.822726	0.9094	3.202888	1.26E-31	3.88E-30	5.982933	6.51E-07	1.78E-05	1.348692	3.83E-14	8.44E-13
Protease	***KLK7***	-0.96658	0.411115	0.633551	NA	NA	NA	NA	NA	NA	0.236977	1.97E-10	1.71E-09
Protease	***KLK8***	1.972258	0.072615	0.303639	NA	NA	NA	NA	NA	NA	0.227031	4.42E-05	0.000127
Protease	***KLK9///KLK8***	NA	NA	NA	2.564622	1.09E-52	3.81E-50	4.752066	3.05E-11	8.6E-09	0.552355	5.15E-18	3.35E-16
Kinin autacoid	***KNG1***	1.480657	0.145105	0.409666	NA	NA	NA	0	1	1	-0.071903	2.00E-10	1.73E-09
Lipid precursor processor	***LOC101928830///LTA4H***	NA	NA	NA	0.074003	0.020633	0.034418	0.002526	0.975098	1	NA	NA	NA
Lipid precursor processor	***LTA4H***	2.804361	0.00593	0.131227	NA	NA	NA	NA	NA	NA	0.071721	1.25E-07	5.95E-07
Lipid receptor	***LTB4R***	2.394749	0.0214	0.198078	NA	NA	NA	NA	NA	NA	0.207296	2.23E-07	1.02E-06
Lipid receptor	***LTB4R2***	1.649014	0.080447	0.315365	0.61524	6.60E-25	1.06E-23	0.013349	0.069959	0.198694	0.019476	0.585217	0.647667
Protein kinase	***MAPK1***	1.513852	0.193617	0.447358	NA	NA	NA	NA	NA	NA	0.003817	0.808362	0.843784
Protease	***MMP9***	0.900806	0.164757	0.426505	1.5588	3.92E-40	3.04E-38	3.314864	1.53E-06	3.45E-05	0.355631	8.66E-09	5.26E-08
Mas-related G-protein coupled receptor	***MRGPRX1***	0.546196	0.504571	0.707391	0.004744	0.734198	0.783838	0	1	1	0.101148	6.00E-07	2.5E-06
Mas-related G-protein coupled receptor	***MRGPRX2***	0.279168	0.779569	0.88543	-0.16444	2.14E-05	5.99E-05	0	1	1	0.091641	3.18E-07	1.4E-06
Mas-related G-protein coupled receptor	***MRGPRX3***	NA	NA	NA	0.207642	1.66E-06	5.35E-06	0	1	1	0.021566	0.194989	0.252686
Mas-related G protein-coupled receptor	***MRGPRX4***	NA	NA	NA	0.016477	0.618388	0.680397	0	1	1	0.042729	0.022294	0.036824
Cell adhesion molecule	***NECTIN3***	1.786841	0.201462	0.454658	NA	NA	NA	NA	NA	NA	0.027379	0.555942	0.619866
Neuropeptide	***NGF***	1.021251	0.3194	0.577367	0.199495	1.70E-05	4.82E-05	0.004506	0.902732	1	-0.061536	0.025403	0.041335
Neuropeptide receptor	***NGFR***	1.217651	0.146909	0.411226	-0.12718	0.002491	0.005025	-0.7065	0.002359	0.012208	-0.048738	0.188125	0.244804
Neuropeptide	***NMB***	-1.00898	0.342966	0.595011	0.161619	0.00088	0.001926	0.60749	0.019994	0.071601	-0.108246	0.004539	0.008694

Neuropeptide receptor	***NMBR***	0.393098	0.428419	0.64575	0.010458	0.506604	0.574155	0	1	1	0.060117	0.064557	0.094957
Neuropeptide	***NMU***	-1.60177	0.108114	0.363936	0.471487	1.30E-08	5.24E-08	-0.1661	0.302775	0.596801	0.032481	0.406609	0.475523
Natriuretic peptide	***NPPA***	-1.66189	0.081866	0.317712	0.120414	0.003444	0.006768	-0.01316	0.415557	0.753109	-0.05611	0.12523	0.171013
Hormone	***NPPB***	0.247277	0.736727	0.861822	0.063626	0.232707	0.295187	7.70E-06	0.512569	0.832112	-0.180619	2.38E-08	1.31E-07
Preproprotein	***NPPC***	0.160518	0.822563	0.909278	0.000719	0.984036	0.988343	0	1	1	-0.09916	0.000382	0.000911
Natriuretic peptide receptor	***NPR1***	2.461709	0.064826	0.292982	NA	NA	NA	NA	NA	NA	-0.229078	1.98E-06	7.4E-06
Natriuretic peptide receptor	***NPR2***	-1.92061	0.105927	0.360132	NA	NA	NA	NA	NA	NA	0.249744	2.75E-09	1.86E-08
Natriuretic peptide receptors	***NPR3***	2.874786	0.014452	0.173963	NA	NA	NA	NA	NA	NA	-0.033352	0.551444	0.61554
Neuropeptide	***NPY***	NA	NA	NA	0.029686	0.306295	0.373383	0	1	1	0.094589	5.21E-05	0.000147
Neuropeptide receptor	***NPY1R***	1.389264	0.031281	0.230009	-0.83591	5.78E-16	4.28E-15	-0.32637	0.212781	0.449125	-0.006852	0.830697	0.862701
Neuropeptide receptor	***NPY2R***	1.936849	0.035854	0.242661	NA	NA	NA	NA	NA	NA	0.110256	3.60E-08	1.91E-07
Neuropeptide receptor	***NPY4R***	NA	NA	NA	NA	NA	NA	NA	NA	NA	-0.021808	0.230796	0.292482
Neuropeptide receptor	***NPY5R***	0.641674	0.574046	0.75758	-0.31783	2.30E-16	1.77E-15	-0.61194	0.06861	0.195578	-0.177388	0.00014	0.000364
Neurotrophic factor	***NRTN***	0.236686	0.856231	0.927377	-0.2743	0.000261	0.000628	-0.00172	0.08622	0.235059	0.083847	0.122622	0.167954
Neurotrophic factor	***NTF4***	0.497244	0.577098	0.759361	NA	NA	NA	NA	NA	NA	0.003721	0.907272	0.925603
Neurotrophic factor receptor	***NTRK1***	1.170401	0.235982	0.491649	0.150811	0.000449	0.001036	0	1	1	-0.01561	0.407998	0.476996
Neurotrophic factor receptor	***NTRK2***	1.242993	0.033819	0.237521	NA	NA	NA	NA	NA	NA	-0.208003	2.80E-07	1.25E-06
Opioid receptor	***OPRK1***	1.030102	0.063838	0.2916	NA	NA	NA	0	1	1	0.023027	0.148698	0.199029
Opioid receptor	***OPRL1***	NA	NA	NA	NA	NA	NA	NA	NA	NA	0.011634	0.368478	0.436898
Purinergic receptor	***P2RX3***	1.099234	0.225637	0.479966	0.094857	0.000579	0.001308	3.08E-05	0.512569	0.832112	-0.055445	0.012012	0.021059
Opioid precursor	***PDYN***	1.879638	0.018635	0.190056	0.132024	4.38E-05	0.000118	0	1	1	-0.010739	0.541239	0.606082
Opioid precursor	***PENK***	NA	NA	NA	NA	NA	NA	NA	NA	NA	0.098515	0.036135	0.056734
Ion channel	***PIEZO2***	1.852126	0.076067	0.309142	NA	NA	NA	NA	NA	NA	-0.022257	0.264664	0.328544
Phospholipase	***PLA2G4B***	0.186709	0.823488	0.909634	NA	NA	NA	NA	NA	NA	NA	NA	NA
Phospholipase	***PLA2G4B///JMJD7-PLA2G4B***	NA	NA	NA	NA	NA	NA	NA	NA	NA	0.417269	8.69E-18	5.39E-16
Phospholipase	***PLA2G4D***	-1.71066	0.088082	0.328764	0.710771	8.41E-35	3.54E-33	5.923484	1.92E-14	3.6E-11	0.888959	1.13E-29	4.13E-26
Phospholipase	***PLA2G4E***	0.378072	0.464821	0.674608	NA	NA	NA	NA	NA	NA	NA	NA	NA
Enzyme	***PLCB3***	0.090042	0.902892	0.952695	0.216994	8.68E-06	2.55E-05	0.343342	0.01741	0.063763	0.067829	8.36E-07	3.38E-06
Enzyme	***PLCG1***	2.60687	0.006618	0.136524	NA	NA	NA	NA	NA	NA	-0.070423	6.21E-08	3.14E-07
Preproprotein	***PNOC***	1.119744	0.185174	0.441405	0.157686	2.79E-07	9.85E-07	0.019438	0.434931	0.779876	0.190254	5.75E-14	1.2E-12
Preproprotein	***POMC***	-1.47353	0.142473	0.407533	0.158349	0.000393	0.000921	0.000877	0.363162	0.682418	-0.129011	4.09E-06	1.44E-05
Protease	***PRSS1***	-0.84994	0.47433	0.682918	NA	NA	NA	NA	NA	NA	0.014812	0.408512	0.477507
Phospholipid receptor	***PTAFR***	-1.24393	0.122025	0.38635	NA	NA	NA	NA	NA	NA	0.286357	3.77E-14	8.31E-13
Lipid precursor processor	***PTGDS***	-1.02887	0.264891	0.52243	NA	NA	NA	NA	NA	NA	0.034556	0.414631	0.483384
Lipid receptor	***PTGER1***	1.332353	0.049271	0.269197	NA	NA	NA	NA	NA	NA	0.037435	0.040178	0.062393
Lipid receptor	***PTGER2***	0.387773	0.829492	0.91299	-0.21199	0.0001	0.000258	-2.15588	1.93E-08	1.34E-06	0.060633	0.124692	0.170374
Lipid receptor	***PTGER3***	2.991676	0.009302	0.153832	NA	NA	NA	NA	NA	NA	0.175439	1.00E-06	3.99E-06
Lipid receptor	***PTGER4***	-1.17005	0.188169	0.442924	NA	NA	NA	NA	NA	NA	0.12092	4.87E-05	0.000138
Lipid precursor processor	***PTGES***	0.921421	0.117366	0.379671	NA	NA	NA	NA	NA	NA	0.046858	0.014775	0.025444
Lipid precursor processor	***PTGES2***	0.166283	0.851808	0.92497	0.433373	4.22E-22	5.31E-21	0.867672	8.90E-05	0.000844	0.026266	0.134925	0.182715
Lipid precursor processor	***PTGES3***	-2.04646	0.007421	0.141781	0.057834	0.001665	0.003466	-0.22581	0.001619	0.008998	0.005665	0.487177	0.554574
Lipid precursor processor	***PTGS1***	1.554054	0.057353	0.282311	NA	NA	NA	NA	NA	NA	-0.042929	0.092948	0.131331
Lipid precursor processor	***PTGS2***	-0.04321	0.965944	0.984974	NA	NA	NA	NA	NA	NA	0.274895	1.51E-07	7.12E-07
Neuropeptide receptor	***RAMP1***	0.491249	0.390609	0.616673	0.093915	0.005165	0.009804	0.038977	0.86382	1	-0.10431	0.000127	0.000334
Oncogene Protein kinase	***RET***	NA	NA	NA	NA	NA	NA	NA	NA	NA	0.081657	0.009486	0.017011
Protein kinase	***ROCK1***	-0.2527	0.688347	0.830079	NA	NA	NA	NA	NA	NA	0.023182	0.200724	0.259193

Protein kinase	***ROCK2***	1.991843	0.04129	0.25534	NA	NA	NA	NA	NA	NA	-0.053193	0.080598	0.115679
Calcium-binding protein	***S100A14***	-2.42515	0.044943	0.263137	0.636258	8.35E-29	1.91E-27	0.130551	0.128118	0.319771	-0.011924	0.530112	0.595618
Calcium binding protein	***S100A2***	0.678292	0.498935	0.702626	1.637603	1.44E-48	2.88E-46	0.87032	1.13E-05	0.000163	0.029012	0.024627	0.040222
Calcium binding protein	***S100A7***	NA	NA	NA	4.194487	1.29E-43	1.41E-41	2.507282	1.05E-06	2.59E-05	0.566343	3.58E-08	1.9E-07
Calcium binding protein	***S100A9***	NA	NA	NA	5.822775	1.58E-74	2.14E-71	8.202938	2.81E-14	4.53E-11	1.220865	5.43E-21	1.12E-18
Calcium-binding protein	***S100G***	NA	NA	NA	0.03276	0.201139	0.260099	0	1	1	0.095994	4.96E-05	0.000141
Calcium binding protein	***S100P***	1.157456	0.186108	0.441889	1.263062	8.06E-13	4.7E-12	2.03594	0.0013	0.007556	0.182192	0.002357	0.004789
Sodium channel	***SCN11A***	1.448908	0.144162	0.409209	NA	NA	NA	NA	NA	NA	-0.021274	0.394098	0.462943
Sodium channel	***SCN3A***	1.730374	0.101778	0.353013	NA	NA	NA	NA	NA	NA	0.228517	3.61E-06	1.28E-05
Sodium channel	***SCN9A***	2.591475	0.000735	0.064646	NA	NA	NA	NA	NA	NA	0.13348	0.00025	0.000618
Cell adhesion molecule	***SELE***	0.302558	0.671358	0.820313	1.517957	1.15E-17	9.81E-17	1.984328	0.000278	0.002148	0.354604	5.23E-07	2.21E-06
Semaphorin	***SEMA3A***	1.153962	0.19591	0.449932	NA	NA	NA	NA	NA	NA	-0.046767	0.024775	0.040419
Hormone	***SST***	NA	NA	NA	0.166071	0.000279	0.000666	0.003346	0.456931	0.80942	0.018427	0.761271	0.803547
Neuropeptide precursor	***TAC1***	0.677359	0.463992	0.673942	-0.41161	8.83E-08	3.3E-07	-3.60961	1.93E-06	4.21E-05	-0.125496	0.119846	0.164636
Neuropeptide receptor	***TACR1***	2.861333	0.000255	0.043294	NA	NA	NA	NA	NA	NA	-0.122631	4.44E-08	2.32E-07
Lipid receptor	***TBXA2R***	1.367008	0.064215	0.292048	NA	NA	NA	NA	NA	NA	0.087472	0.000241	0.000598
Monooxygenase	***TBXAS1***	-0.147	0.880109	0.939946	NA	NA	NA	NA	NA	NA	-0.000151	0.996608	0.997017
Toll-like receptor	***TLR4***	3.586356	0.000351	0.048303	NA	NA	NA	NA	NA	NA	0.046314	0.26022	0.323889
Toll-like receptor	***TLR7***	0.705429	0.497381	0.701402	NA	NA	NA	NA	NA	NA	0.292512	5.03E-07	2.13E-06
Cytokine	***TNF***	1.887683	0.047924	0.267249	0.342811	1.01E-14	6.78E-14	1.645224	0.001039	0.006376	0.22136	2.25E-07	1.02E-06
Cytokine receptor	***TNFRSF1A***	-1.16489	0.07863	0.312439	0.323594	1.69E-16	1.32E-15	0.012167	0.857356	1	0.008444	0.360378	0.428453
Cytokine receptor	***TNFRSF1B***	3.086932	0.020835	0.197454	0.397734	2.03E-12	1.15E-11	0.676837	0.006059	0.026419	0.058097	0.015316	0.026286
Neurotransmitter precursor processor	***TPH1***	0.473837	0.594354	0.771946	NA	NA	NA	NA	NA	NA	0.111535	0.017596	0.029804
Neurotransmitter precursor processor	***TPH2***	1.055258	0.232288	0.48791	-0.02962	0.156118	0.208583	0	1	1	0.044128	0.067269	0.098537
Protease	***TPSAB1***	0.609135	0.517191	0.717079	NA	NA	NA	NA	NA	NA	-0.113214	0.029341	0.047069
Ion channel	***TRPA1***	-0.47238	0.372892	0.603469	NA	NA	NA	0	1	1	0.040926	0.001801	0.003741
Ion channel	***TRPM8***	0.837212	0.402286	0.626151	NA	NA	NA	NA	NA	NA	-0.144634	1.26E-10	1.14E-09
Ion channel	***TRPV1***	-1.44675	0.078857	0.312728	NA	NA	NA	NA	NA	NA	0.21604	6.73E-12	8.28E-11
Ion channel	***TRPV2***	0.757091	0.367827	0.601318	NA	NA	NA	NA	NA	NA	-0.002348	0.885195	0.907356
Ion channel	***TRPV3***	-1.0022	0.093736	0.338057	NA	NA	NA	NA	NA	NA	0.087349	6.05E-05	0.000168
Ion channel	***TRPV4***	0.789619	0.333981	0.589184	0.04254	0.458748	0.528695	-0.0034	0.145591	0.333033	-0.144138	8.91E-09	5.39E-08
Cytokine	***TSLP***	-0.17176	0.885689	0.943314	0.389038	5.76E-08	2.19E-07	0.9582	0.003145	0.015495	0.39251	9.21E-10	6.93E-09
Nerve fibre marker	***UCHL1***	0.373584	0.577153	0.759402	NA	NA	NA	NA	NA	NA	-0.063184	0.10559	0.147155
Neuropeptide	***VIP***	NA	NA	NA	-0.09622	2.03E-05	5.7E-05	0.001154	0.453049	0.803932	-0.138538	0.023603	0.038773
Neuropeptide receptor	***VIPR1***	1.469427	0.167699	0.428595	0.068235	0.350225	0.419399	0.458606	0.030573	0.101257	0.136294	1.35E-06	5.2E-06
Neuropeptide receptor	***VIPR2***	0.817799	0.154462	0.419112	NA	NA	NA	NA	NA	NA	-0.038557	0.036934	0.057847
